# Dendrobine inhibits anaplastic thyroid cancer progression by targeting the JAK-STAT3 pathway

**DOI:** 10.3389/fonc.2026.1842670

**Published:** 2026-06-02

**Authors:** Yanhuan Shang, Dan Zhang

**Affiliations:** Department of Endocrinology and Metabolism, The Third Affiliated Hospital of Jinzhou Medical University, Jinzhou, Liaoning, China

**Keywords:** anaplastic thyroid carcinoma, dendrobine, invasion, JAK-STAT3 pathway, migration, proliferation

## Abstract

**Objective:**

This study aimed to investigate the antitumor effects of dendrobine on ATC cells and its mechanism targeting the JAK-STAT3 pathway.

**Methods:**

Human ATC cell lines (CAL-62 and 8505C) were treated with dendrobine (DEM). Detect cell viability, cell proliferation, cell apoptosis, cell migration and invasion. The expressions of E-cad, N-cad and Vim were detected by IF. JAK1, JAK2, STAT3 and their phosphorylated forms were quantified by WB. To probe pathway reversibility, cells were pretreated with IL-6 (20 ng/mL) or STAT3-siRNA before dendrobine exposure. *In vivo*, 6-week-old male BALB/c nude mice with 8505C xenografts were administered dendrobine (10 or 40 mg/kg/d) or saline for 21 d.

**Results:**

Dendrobine reduced the viability of CAL-62 and 8505C cells, with 5 μM dendrobine achieving the most pronounced inhibition (*P* < 0.001). Dendrobine treatment markedly suppressed cell proliferation (*P* < 0.05) and colony formation (*P* < 0.001), while increasing apoptosis (*P* < 0.01). Dendrobine also inhibited migration and invasion (*P* < 0.05), upregulated E-cad expression (*P* < 0.001), downregulated N-cad and Vim expression (*P* < 0.05), suggesting EMT inhibition. WB analysis confirmed that dendrobine suppressed phosphorylation of JAK1, JAK2, and STAT3 (*P* < 0.05). However, IL-6 pretreatment reversed these effects by restoring p-JAK1, p-JAK2, and p-STAT3 levels (*P* < 0.001) and partially rescuing cell proliferation and migration (*P* < 0.001). Conversely, STAT3-siRNA transfection enhanced dendrobine’s inhibitory effects on proliferation, invasion, and migration (*P* < 0.05) and further reduced JAK-STAT3 phosphorylation (*P* < 0.05). *In vivo*, Dendrobine reduced tumor volume and weight (*P* < 0.001), decreased IL-6 content in tumors (*P* < 0.05), improved liver function indices by reducing AST, ALT and ALP activities (*P* < 0.05), and exhibited no nephrotoxic effects.

**Conclusion:**

Dendrobine exerts antitumor effects in ATC by targeting the JAK-STAT3 pathway, providing a promising therapeutic candidate.

## 1 Introduction

Thyroid cancer is the most common endocrine malignancy. Among its subtypes, ATC is recognized as the rarest form, accounting for for less than 0.2% to 1%-2% of all thyroid cancers (1). ATC demonstrates poor prognosis and resistance to multiple therapeutic approaches, highlighting the urgent need for novel targeted therapies (2). Dysregulation of the JAK-signal transducer and activator of STAT3 pathway is frequently documented across diverse malignancies and underpins core malignant behaviors (3). In ATC, heightened activity within this axis may correlate with worsened prognosis. Studies have identified high expression levels of stem cell markers and p-STAT3 in ATC cells (4). Genomic profiling from The Cancer Genome Atlas reveals that upregulated JAK-STAT pathway activity correlates with reduced recurrence-free survival in patients with thyroid carcinoma, positioning this signaling axis as a viable target for anti-cancer interventions (5). Recent studies have begun to focus on the therapeutic potential of natural compounds targeting the JAK-STAT3 pathway in thyroid cancer (6). Dendrobine is an active alkaloid extracted from the traditional Chinese medicinal herb *Dendrobium*, demonstrating anti-inflammatory, neuroprotective, and potential antitumor effects (7). Existing experimental evidence confirms that dendrobine acts on the STAT3/FoxO pathway, effectively suppressing the progression of cellular senescence (8). However, its mechanism of action in thyroid cancer, particularly ATC, remains unclarified. As a natural plant-derived compound, dendrobine possesses safety advantages and may exhibit distinct mechanisms from conventional chemotherapeutic agents due to its unique chemical structure (9). This study systematically investigates the effects of dendrobine on ATC cells and its relationship with the JAK-STAT3 pathway, aiming to advance the understanding of natural antitumor drug mechanisms.

## 2 Materials and methods

### 2.1 Drugs and reagents

FBS (A5670801), RPMI-1640 medium (11875093), PBS (10010023), penicillin/streptomycin solution (15140148) and 0.25% trypsin digestion solution (25200056) were obtained from Gibco (Shanghai, China). Matrix-Gel™ Basement Membrane Matrix (C0371), crystal violet (Y268091), RIPA lysis buffer (P0013B), protease inhibitor (P1006), phosphatase inhibitor (P1045), hypersensitive ECL chemiluminescent substrate kit (P0018FS), 4% paraformaldehyde (P0099), HE staining kit (C0105S), eco-friendly dewaxing agent (ST975), Phalloidin-TRITC (915013-10-4), DAPI Staining Solution (C1006), BCA protein concentration assay kit (P0399S) and immunostaining permeabilization buffer (P0095) were procured from Beyotime (Shanghai, China). EdU assay kit was acquired from Ribobio (Guangzhou, China). Antibodies against JAK1 (ab278781), p-JAK2 (ab219728), p-STAT3 (ab32143), E-Cad (ab314063), p-JAK1 (ab138005), N-Cad (ab245117), STAT3 (ab68153), Vim (ab92547), JAK2 (ab32101), goat anti-rabbit secondary antibody (ab6721), human TNF-α kit (PT518), human IL-6 kit (PI330), mouse AST kit (ab263882), mouse ALT kit (ab282882) and mouse ALP kit (ab267583) were supplied by Abcam (Shanghai, China). Mouse Cr kit (SP15017) and mouse BUN kit (SP30153) were purchased from spbio (Wuhan, China).

### 2.2 Cell lines and cell culture

The human ATC cell line CAL-62 was acquired from the National Collection of Authenticated Cell Cultures (Shanghai, China). Anothernormal thyroid cell line 8505C was acquired from Gelatins (Shanghai, China). Human normal thyroid cell line NTHY-ORI 3–1 were supplied by Huatao Biotechnology (Shenzhen, China). All cells have short tandem repeat (STR) authentication and were cultured in RPMI-1640 medium with 10% FBS and 0.1 mg/ml penicillin/streptomycin solution. The cells were maintained at 37°C with 5% CO_2_. CAL-62 and 8505C cell lines, including siRNA-transfected CAL-62 and 8505C cells, were harvested after 24 h treatment with dendrobine (dissolved in DMSO) at specified concentrations for subsequent experiments. For IL-6 treatment, CAL-62 and 8505C cells were pre-treated with 20 ng/mL IL-6 for 1 h prior to 5 μM dendrobine administration. Each group consisted of 3 replicate wells.

### 2.3 CCK-8 assay

Cell viability was measured using CCK-8. 2000 cells/well were seeded on 96-well plates at 37°C and adhered to the wall overnight. Cells were treated with various concentrations of dendrobine (0, 1, 2, 3, 4, 5 μM) for 24 h (10, 11). The culture medium was then replaced with 100 µL of fresh medium containing 10% CCK-8 reagent, and the absorbance was measured to determine cell viability.

### 2.4 EdU assay

Cell proliferation was evaluated using the EdU assay. Cells were seeded in 24-well plates at a density of 1 × 10^4^ cells/well. Coverslips with and without the treatments were fixed with absolute ethanol for 10 min, rinsed 3 times with PBS, then permeabilized with permeabilization solution containing 0.3% Triton X-100 for 15 min. EdU determination was performed according to the manufacturer’s instructions.

### 2.5 Colony formation assay

Cells were placed in 6-well plates with an initial density of 2 × 10^4^ cells/well containing dendrobine for 7 d. After forming colonies, the plates were fixed with 4% paraformaldehyde for 15 min, and colored with 0.1% crystal violet for 20 min.

### 2.6 Annexin V-FITC/PI double staining

Cell apoptosis was detected by flow cytometry using Annexin V-FITC/PI double staining. Cells were seeded in 6-well plates at a density of 2 × 10^5^ cells/well. After treatment with dendrobine for 24 h, cells were collected and resuspended in 100 μL binding buffer, followed by incubation with 5 μL Annexin V-FITC and 5 μL PI in the dark at room temperature for 15 min. Subsequently, 400 μL binding buffer was added to each sample, and the stained cells were immediately analyzed by flow cytometry within 1 h.

### 2.7 Transwell assay

Cells were plated in the upper chamber of Transwell inserts coated with Matrigel using a serum-free medium at a density of 3 × 10^4^ cells. The lower chamber was loaded with RPMI-1640 medium supplemented with 10% FBS, along with varying concentrations of dendrobine. The culture dishes were incubated at 37°C with 5% CO_2_ for 48 h. Cells were fixed with 4% paraformaldehyde solution for 30 min and subsequently stained with 0.01% crystal violet for 20 min. Cells were observed under an Nikon ECLIPSE Ni-U microscope (Tokyo, Japan), and their numbers were quantified using ImageJ software.

### 2.8 Cell wound healing assay

Cells were cultured in 6-well plates at 2 × 10^5^ cells/well for 24 h. A scratch wound was created in the confluent cell monolayer using a 100 µL pipette tip. The culture medium was replaced with serum-free medium. After 48 h, the cells were washed twice with fresh medium to remove non-adherent cells, followed by microscopic observation and image acquisition. The wound healing rate was calculated as [(wound width at 0 h - wound width at 24 or 48 h)/wound width at 0 h] × 100%.

### 2.9 Immunofluorescence assay

Cells were seeded in 24-well plates on coverslips at a density of 5 × 10^4^ cells/well and cultured to 70-80% confluence. Then cells were fixed with 4% paraformaldehyde at room temperature for 30 min, followed by permeabilization and blocking with 0.1% Triton X-100 in 5% BSA for 20 min. The samples were incubated overnight at 4°C with primary antibodies against E-cadherin, N-cadherin, and Vimentin, then treated with Alexa-conjugated secondary antibodies at room temperature for 1 h. After staining with 10 µM phalloidin-TRITC for 30 min, nuclei were counterstained with DAPI. Fluorescence imaging was performed using an Olympus DP73 microscope (Tokyo, Japan).

### 2.10 Western blotting

WB was used to analyze the protein expression levels of JAK1, JAK2, STAT3, and their phosphorylated forms (p-JAK1, p-JAK2, p-STAT3). Cells treated with dendrobine, IL-6, or STAT3-siRNA were lysed. Total protein was extracted using RIPA buffer, quantified by BCA assay, and 20 µg/lane of protein was loaded for SDS-PAGE gel electrophoresis and transferred to nitrocellulose membrane. The blot was blocked with 5% non-fat milk and then incubated with primary antibodies overnight at 4°C and then incubated with the secondary antibody for 1.5 h on a room temperature. The expression levels of the proteins were detected using the ECL chemiluminescent substrate kit. GAPDH was used as the internal control. The signal of protein bands was quantified by ImageJ software.

### 2.11 siRNA transfection

**Figure 1:**
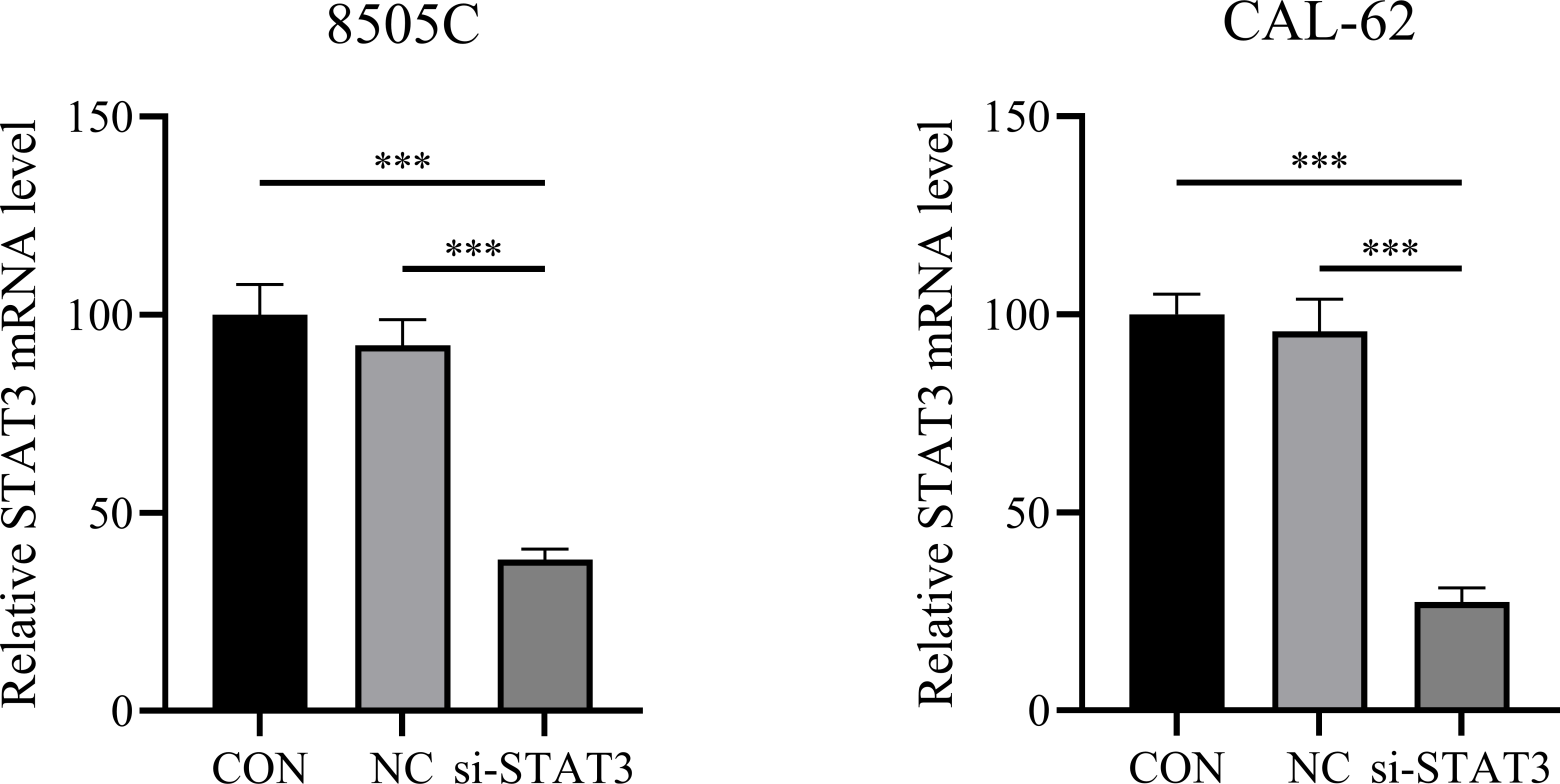
si-STAT3 transfection in 8050C cells and CAL-62 cells.

Beijing Tsingke Biotech Co., Ltd., China, synthesized the siRNA that targets STAT3 and the negative control siRNA. The siRNAs sequence were as follows. STAT3 sense: 5′-GGCTGGACAATATCATTGA-3′. STAT3 antisense: 5′-AUAAAGCCCAUGAUGUACCTT-3′. Negative control sense: 5′-UUCUCCGAACGUGUCACGUTT-3′. Negative control antisense: 5′-ACGUGACACGUUCGGAGAATT-3′. Cells were seeded in 6-well plates and allowed to reach 50-60% confluence before transfection. STAT3-siRNA and negative control siRNA were transfected into ATC cells using Lipofectamine 3000 transfection reagent according to the manufacturer’s protocol. Incubate at 37°C with 5% CO_2_ for 6 h, then replace the transfection medium with the complete growth medium. Cells were harvested 48 h post-transfection for subsequent experiments. The knockdown efficiency of STAT3 was verified by qPCR, which showed significant reduction in STAT3 mRNA levels compared to cells transfected with negative control siRNA or the untransfected cells (**Figure 1**). This confirmed the successful silencing of STAT3 expression in the transfected cells.

### 2.12 Xenograft tumor model

6-week-old male BALB/c nude mice were purchased from Beijing Vital River Laboratory Animal Technology Co., Ltd. All experimental procedures were conducted in accordance with relevant laboratory animal guidelines, and all animal experimental protocols were approved by the Medical Ethics Committee of the Third Affiliated Hospital of Jinzhou Medical University (Approval number: 2023 Lunyanpi 032). A xenograft tumor model was established by subcutaneously injecting 8505C cells (5×10^6^ cells in 100 μL PBS) into the right flank of mice. After tumor formation (approximately 100 mm^3^), the mice were randomly divided into 3 groups (*n* = 6): Control (intragastric administration of saline at a volume of 1 ml daily), Low-dose dendrobine (intragastric administration of 10 mg/kg/d dendrobium at a volume of 1 ml) (12, 13), and High-dose dendrobine (intragastric administration of 40 mg/kg/d dendrobium at a volume of 1 ml) (13). Dendrobine was first dissolved in DMSO and subsequently diluted in normal saline to mitigate the toxicity. Drug administration and data recording were conducted by the experimenters who not involved in group allocation. Tumor volume was measured twice per week. Tumor volume = length × width^2^ × 0.5. On day 21 post-treatment, all mice were euthanized via the CO_2_ inhalation method. The mice were placed in a sealed CO_2_ exposure chamber, and CO_2_ gas was introduced gradually until complete respiratory arrest was observed. Cervical dislocation was then performed to ensure euthanasia. Subsequently, the tumors were excised, weighed, and photographed. Tumor tissues were either fixed in 4% paraformaldehyde for histological analysis or snap-frozen in liquid nitrogen for further molecular studies. Additionally, heart, liver and kidney were collected for histopathological examination to evaluate potential systemic toxicity.

### 2.13 H&E staining

H&E staining was performed on tumor tissues and other organs (heart, liver, kidney). Excised tissues were fixed in 4% paraformaldehyde for 24 h, followed by dehydration in a graded ethanol series and clearing with eco-friendly dewaxing agent. The tissues were embedded in paraffin blocks and sectioned into 4 μm slices. After deparaffinization and rehydration, sections were stained with hematoxylin for 5 min and then counterstaining with eosin for 2 min. The stained sections were dehydrated again and mounted with neutral resin for microscopic examination.

### 2.14 ELISA

ELISA was used to detect the levels of inflammatory cytokines (IL-6, TNF-α) in tumor tissues and liver and kidney injury indicators (AST, ALT, ALP, creatinine, urea nitrogen) in mice serum. All experimental procedures were strictly conducted according to the manufacturer’s instructions.

### 2.15 Statistical analysis

Data are expressed as mean ± standard deviation (SD), and error bars in figures represent SD. Differences between two groups were evaluated using Student’s t-test, comparisons among multiple groups were conducted with one-way analysis of variance (ANOVA), followed by Tukey’s HSD *post hoc* test for pairwise comparisons. Statistical significance was defined as *P* < 0.05. Data analysis was performed with GraphPad Prism 9 software.

## 3 Results

### 3.1 Dendrobine dosage exploration

**Figure 2:**
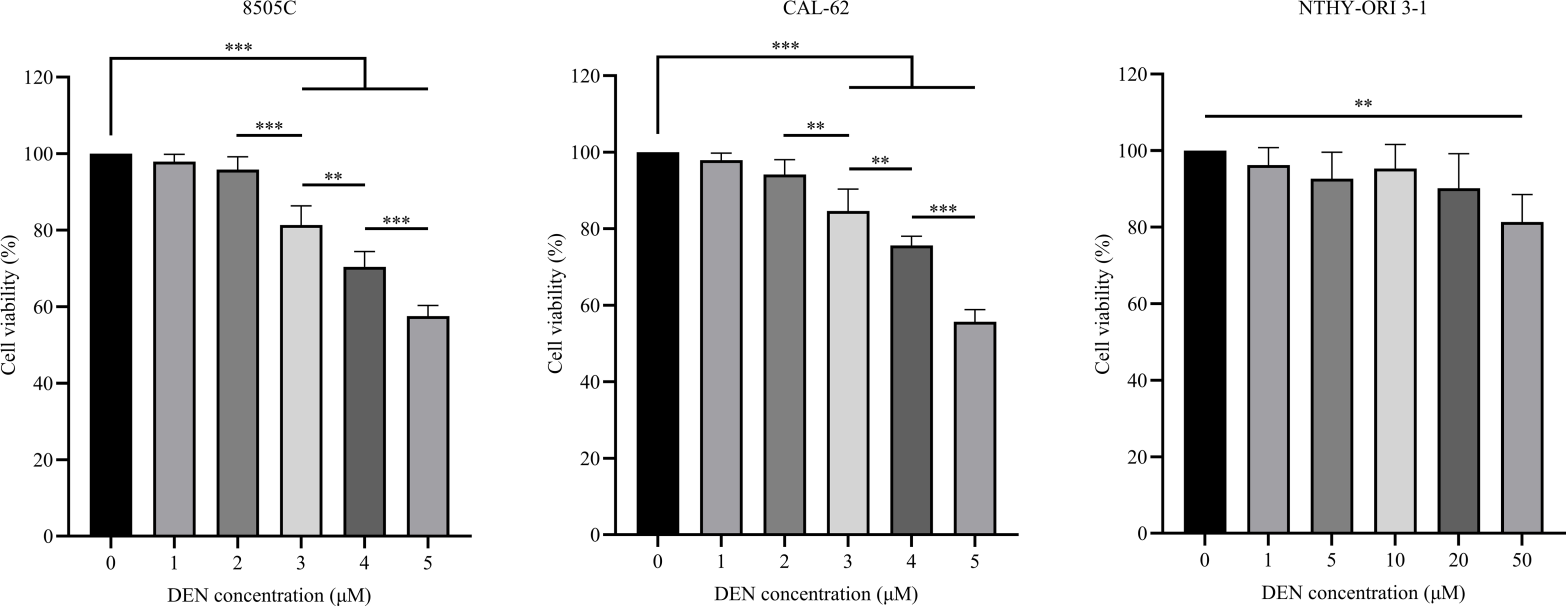
Cell viability of 8505C, CAL-62 and NTHY-ORI 3–1 cells after 24 h of dendrobine (DEN) treatment (*n* = 3). ^**^P < 0.01, ^***^P < 0.001. Same as the figure below.

To determine the safe and effective dosing concentrations for subsequent functional experiments, ATC cells were treated with DEN for 24 h. CCK-8 assays revealed a dose-dependent significant decrease in cell viability (*P* < 0.01, **Figure 2**). Specifically, DEN at 3 μM exhibited a significant inhibitory effect on the viability of both CAL-62 and 8505C cells (*P* < 0.05), and the inhibitory effect reached the most prominent level at the concentration of 5 μM (*P* < 0.001). Based on these results, 3 and 5 μM DEN concentrations were selected for subsequent experiments. Additionally, we treated normal human thyroid cells NTHY-ORI 3–1 with DEN, and the results showed that DEN exhibited cytotoxicity against NTHY-ORI 3–1 cells only when the concentration increased to 50 μM (*P* < 0.01).

### 3.2 Dendrobine significantly inhibits proliferation and induces apoptosis in ATC cells

**Figure 3:**
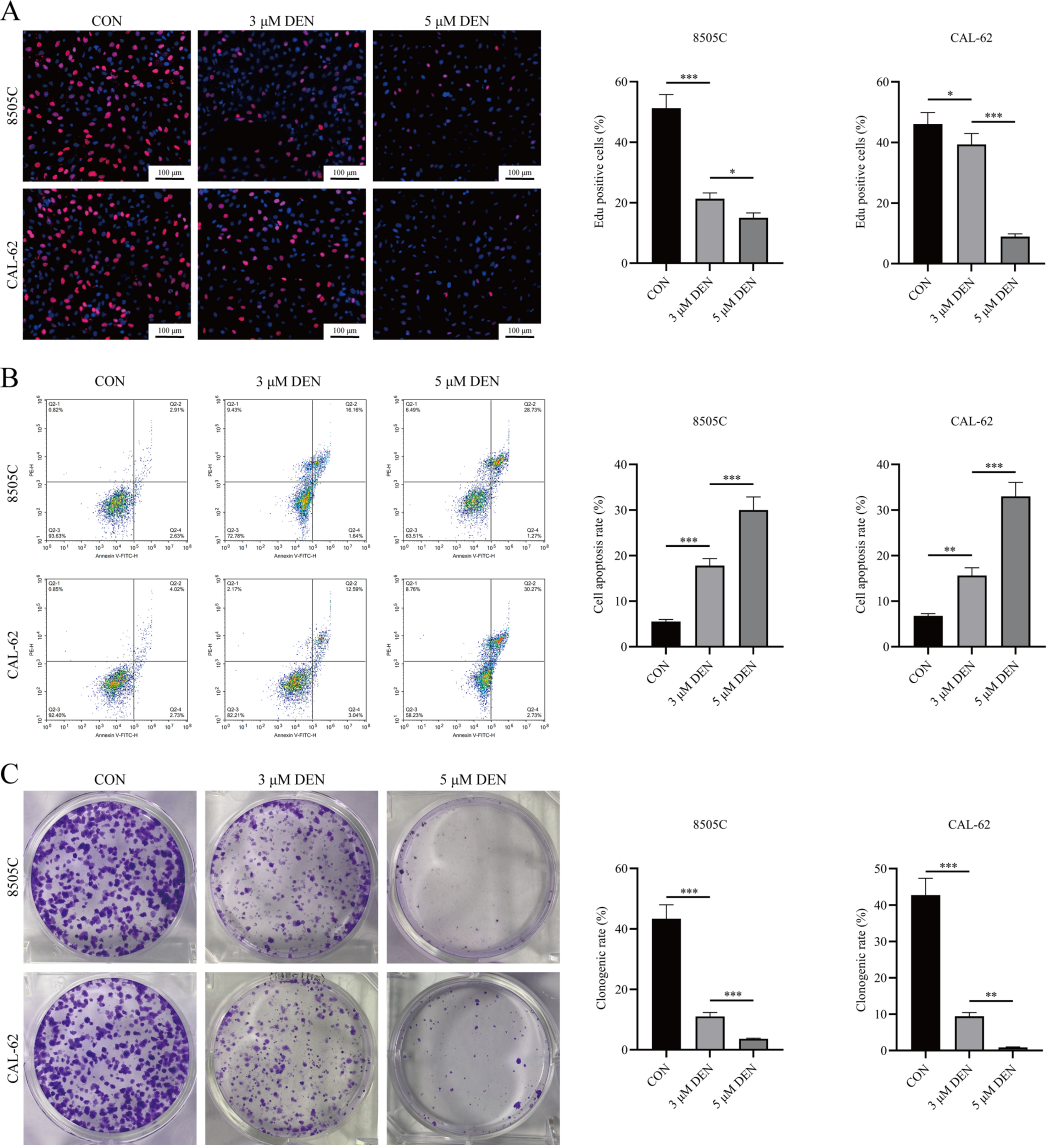
**(A)** EdU staining of 8505C and CAL-62 cells (EdU, red; DAPI, blue) (*n* = 3). **(B)** Flow cytometry analysis of 8505C and CAL-62 cells using FITC-Annexin V and PI double staining (*n* = 3). **(C)** Colony formation assay of 8505C and CAL-62 cells (*n* = 3). CON, control; DEN, dendrobine.

Abnormal proliferation and apoptosis resistance are core malignant hallmarks of tumors and key drivers of ATC progression. Thus, we systematically evaluated the regulatory effect of DEN on the proliferation and apoptosis of ATC cells via EdU assay, colony formation assay, and flow cytometry-based apoptosis detection. EdU staining showed that compared with the control group, the DEN-treated group showed a significant decrease in cell proliferation (*P* < 0.05, **Figure 3A**). Annexin V-FITC/PI double staining flow cytometry further confirmed that the total apoptotic rate of both CAL-62 and 8505C cells was markedly elevated after DEN exposure (*P* < 0.01, **Figure 3B**). Meanwhile, DEN treatment resulted in a significant reduction in colony formation of ATC cells, especially in the DEN-H group (*P* < 0.001, **Figure 3C**).

### 3.3 Dendrobine inhibits invasion, migration, and EMT in ATC cells

**Figure 4:**
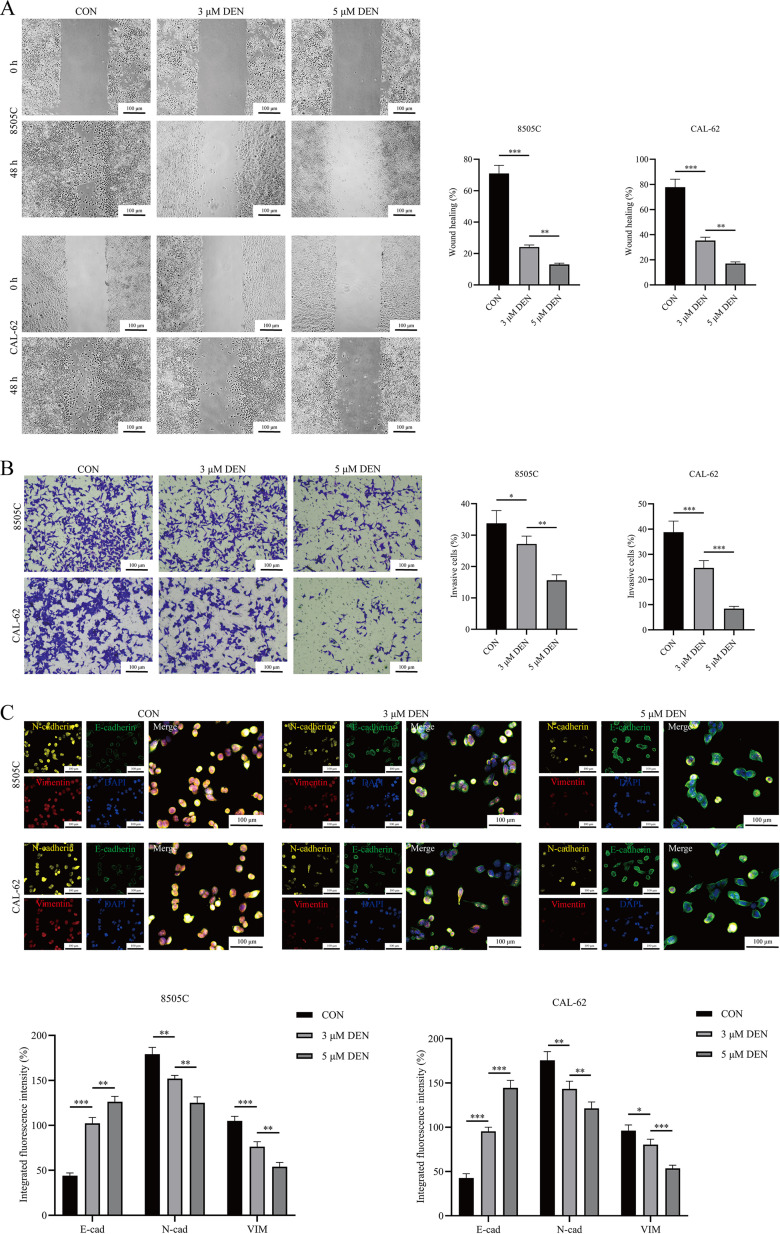
**(A)** Wound healing assay of 8505C and CAL-62 cells (*n* = 3). **(B)** Transwell assay of 8505C and CAL-62 cells (*n* = 3). **(C)** Immunofluorescence detection of E-cadherin (E-cad), N-cadherin (N-cad) and Vimentin (Vim) protein expression levels in 8505C and CAL-62 cells (*n* = 3). CON, control; DEN, dendrobine.

As is well known, EMT is a key biological process for tumor cells to acquire invasive and migratory abilities. Therefore, we performed wound healing assay, Transwell migration and invasion assays, combined with immunofluorescence staining, to explore the effect of DEN on the motility and EMT process of ATC cells. The wound healing assay showed that the scratch closure rate of CAL-62 and 8505C cells in the DEN-treated groups was significantly lower than that in the control group (*P* < 0.001, **Figure 4A**). Transwell assay further confirmed the inhibitory effect of DEN on ATC cell motility, with the most significant effect observed in the DEN-H group (*P* < 0.001, **Figure 4B**). Additionally, IF staining showed that DEN significantly upregulated E-cad expression (*P* < 0.001) and downregulated N-cad and Vim expression (*P* < 0.05), indicating suppression of EMT (**Figure 4C**).

### 3.4 Dendrobine suppresses JAK-STAT3 signaling pathway in ATC cells

**Figure 5:**
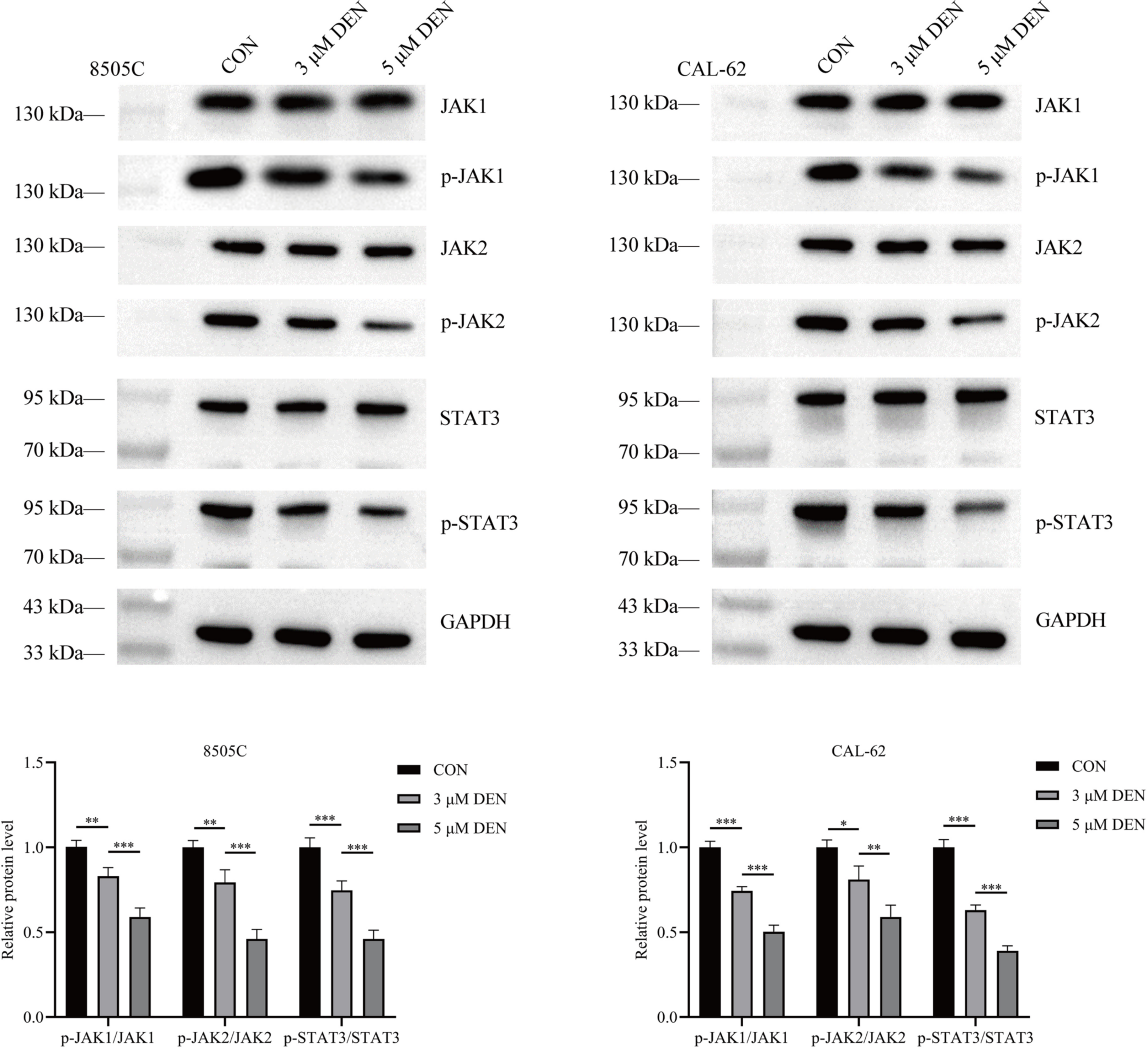
Western blotting of relative protein levels of phosphorylated janus kinase 1 (p-JAK1)/JAK1, p-JAK2/JAK2 and phosphorylated signal transducer and activator of transcription 3 (p-STAT3)/STAT3 in 8505C and CAL-62 cells (*n* = 3). CON, control; DEN, dendrobine.

To clarify the potential molecular target of DEN against ATC, we detected the effect of DEN on the activation of core molecules in the JAK-STAT3 pathway in ATC cells via WB analysis. WB analysis revealed that DEN treatment reduced the relative protein levels of p-JAK1/JAK1, p-JAK2/JAK2 and p-STAT3/STAT3 in cells (*P* < 0.05), with the DEN-H group exhibiting a more pronounced effect (*P* < 0.01, **Figure 5**). These results confirmed that DEN effectively suppresses the hyperactivation of the JAK-STAT3 signaling pathway in ATC cells mainly by targeting the phosphorylation activation of core molecules, rather than affecting the expression of total proteins.

### 3.5 Inflammatory factors activate JAK-STAT3 signaling, reversing the effects of dendrobine

**Figure 6:**
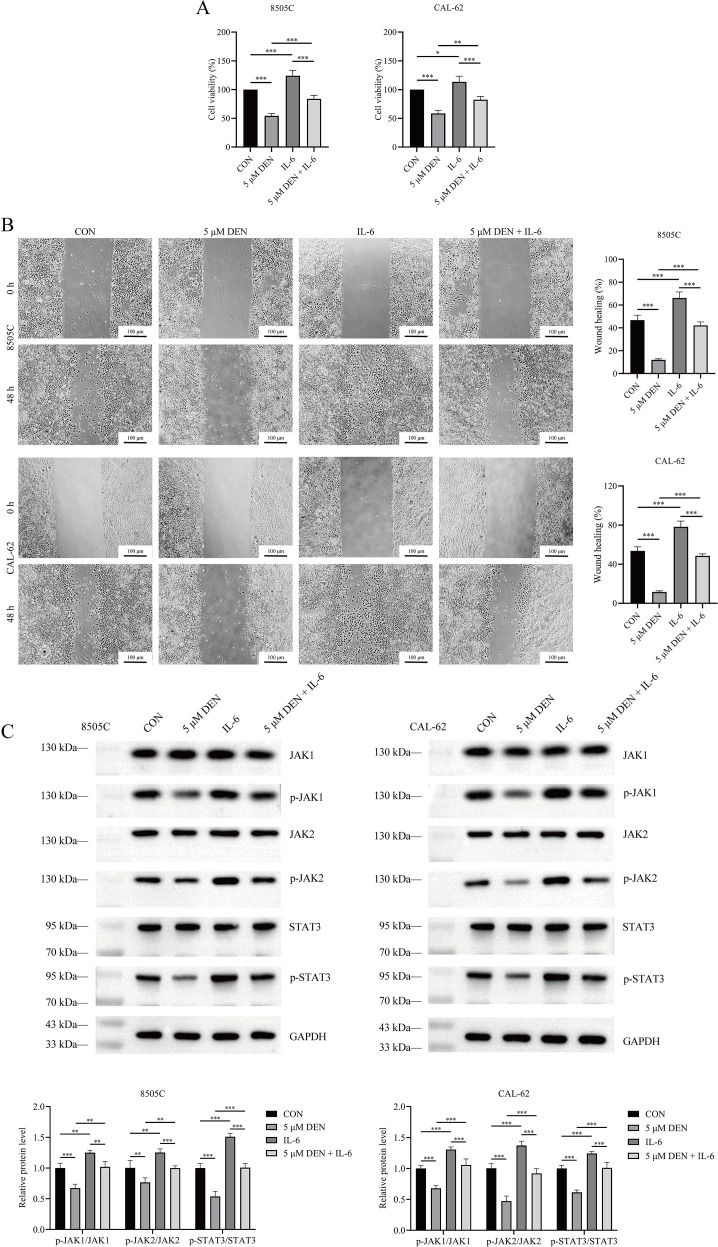
**(A)** Cell viability of 8505C and CAL-62 cells (*n* = 3). **(B)** Wound healing assay of 8505C and CAL-62 cells (*n* = 3). **(C)** Western blotting of relative protein levels of phosphorylated janus kinase 1 (p-JAK1)/JAK1, p-JAK2/JAK2 and phosphorylated signal transducer and activator of transcription 3 (p-STAT3)/STAT3 in 8505C and CAL-62 cells (*n* = 3). CON, control; DEN, dendrobine; IL-6, interleukin-6.

To further verify that DEN exerts anti-ATC effects by targeting the JAK-STAT3 pathway, we pretreated ATC cells with 20 ng/mL IL-6 for 1 h before high-dose DEN exposure, and performed a rescue experiment to confirm the reversal effect of pathway activation on the anti-tumor efficacy of DEN. CCK-8 and wound healing assay showed that IL-6 presence substantially enhanced the proliferative and migratory capacities of DEN-treated cells (P < 0.01), restoring them to levels comparable to the control group (**Figures 6A, B**). Concurrently, WB results showed that IL-6 significantly activated the JAK-STAT3 signaling pathway in DEN-treated ATC cells (P < 0.001, **Figure 6C**).

### 3.6 STAT3-siRNA enhances the effects of dendrobine in ATC cells

**Figure 7:**
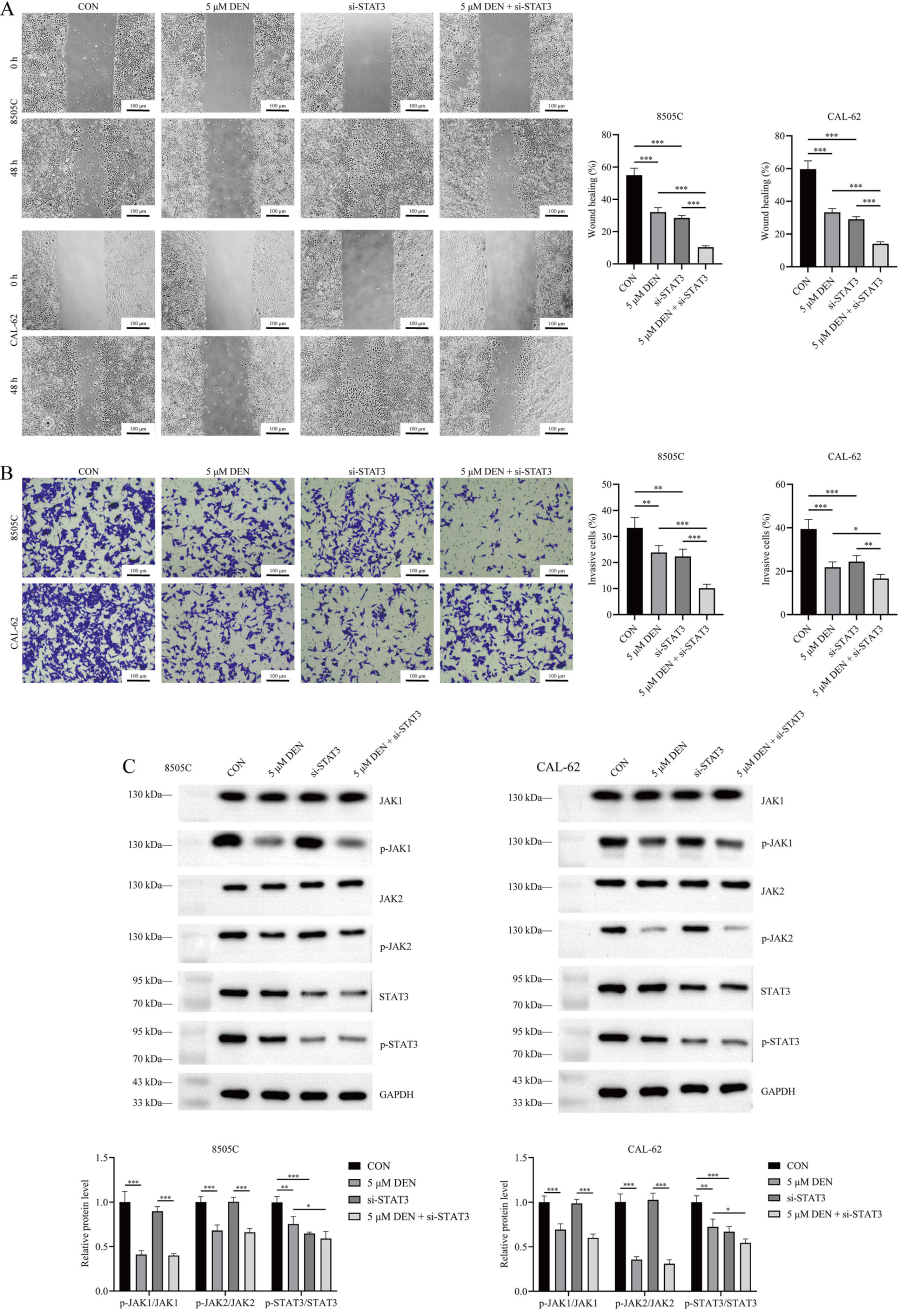
**(A)** Wound healing assay of 8505C and CAL-62 cells (*n* = 3). **(B)** Transwell assay of 8505C and CAL-62 cells (*n* = 3). **(C)** Western blotting of relative protein levels of phosphorylated janus kinase 1 (p-JAK1)/JAK1, p-JAK2/JAK2 and phosphorylated signal transducer and activator of transcription 3 (p-STAT3)/STAT3 in 8505C and CAL-62 cells (*n* = 3). CON, control; DEN, dendrobine.

To further clarify the core mediating role of STAT3 in the anti-ATC process of DEN, we transfected CAL-62 and 8505C cells with STAT3-siRNA, followed by high-dose DEN treatment, to explore the effect of STAT3 silencing on the anti-tumor efficacy of DEN. **Figure 1** shows the successful transfection of STAT3-siRNA. Wound healing and transwell assay showed that, combined treatment with STAT3-siRNA can significantly enhance the inhibitory effect of DEN on the proliferation, migration and invasion of ATC cells (*P* < 0.05, **Figures 7A, B**). WB results showed that the combined treatment further reduced the relative ratios of p-JAK1/JAK1, p-JAK2/JAK2 and p-STAT3/STAT3 (*P* < 0.05, **Figure 7C**), indicating that STAT3 knockdown synergized with DEN to inhibit the activation of the JAK-STAT3 signaling pathway more efficiently. These gene silencing experiment results further confirmed that STAT3 is the core target of DEN against ATC.

### 3.7 Dendrobine inhibits tumor proliferation and invasion through JAK-STAT3 pathway *in vivo*

**Figure 8:**
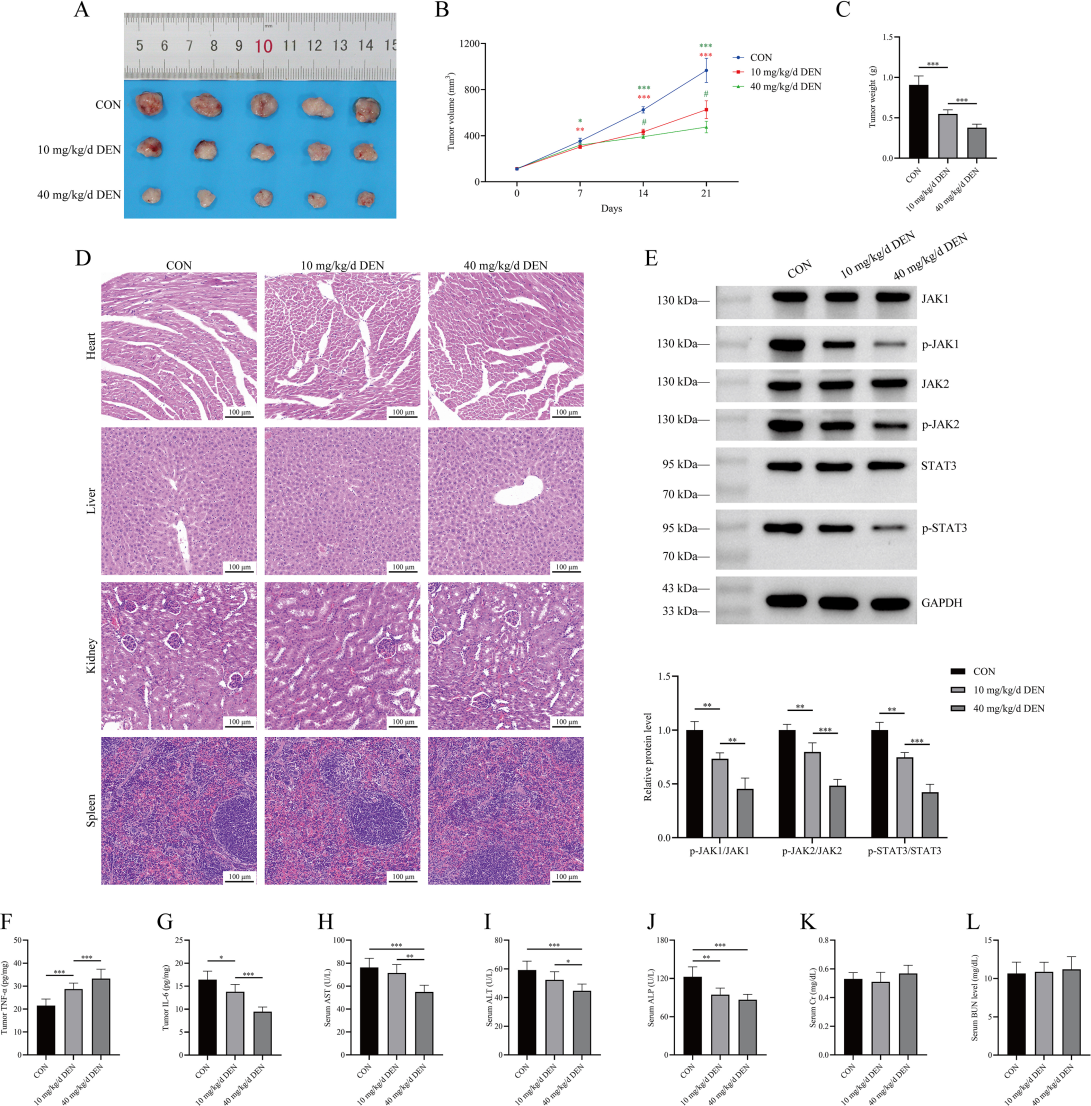
**(A)** Representative tumor images. **(B)** Tumor volume (*n* = 5). **(C)** Tumor weight (*n* = 5). **(D)** Representative H&E staining images of heart, liver, spleen and kidney tissues. **(E)** Western blotting of relative protein levels of phosphorylated janus kinase 1 (p-JAK1)/JAK1, p-JAK2/JAK2 and phosphorylated signal transducer and activator of transcription 3 (p-STAT3)/STAT3 in mice tumor tissues (*n* = 5). **(F)** tumor necrosis factor-alpha (TNF-α) content in tumor tissues (*n* = 5). **(G)** Interleukin-6 (IL-6) content in tumor tissues (*n* = 5). **(H)** Serum aspartate aminotransferase (AST) level. **(I)** Serum alanine aminotransferase (ALT) level (*n* = 5). **(J)** Serum alkaline phosphatase (ALP) level (*n* = 5). **(K)** Serum creatinine **(Cr)** level (*n* = 5). **(L)** Serum blood urea nitrogen (BUN) level (*n* = 5). CON: control. DEN: dendrobine. ^*^P < 0.05, ^**^P < 0.01, ^***^P < 0.001. *Compared with the CON group, # compared with the 10 mg/kg/d dendrobine (DEN) group.

**Figure 9:**
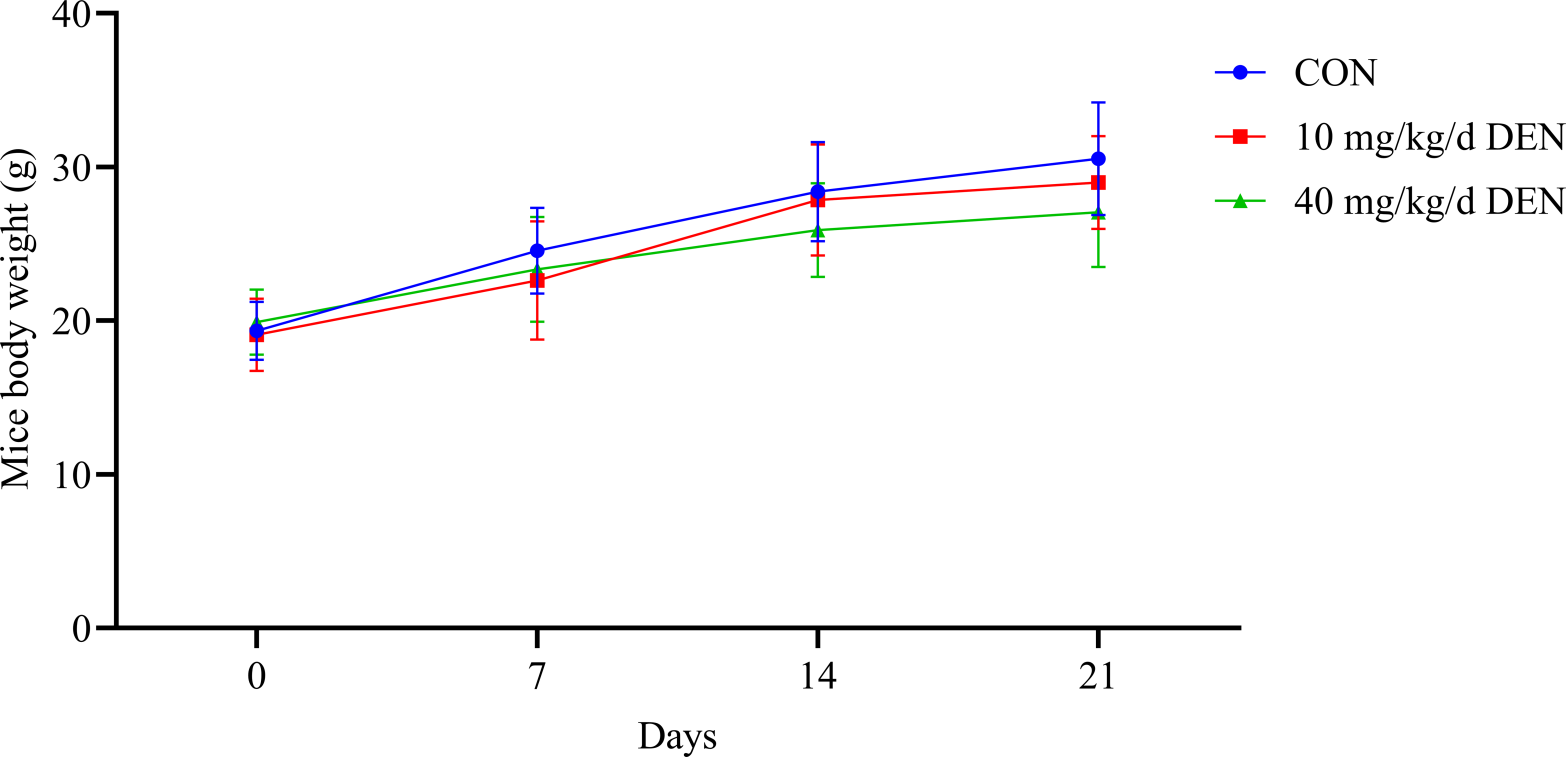
Body weight of mice.

To verify the anti-ATC effect and molecular mechanism of DEN *in vivo*, we constructed a subcutaneous xenograft tumor model of 8505C cells in BALB/c nude mice. Compared to the control group, DEN administration significantly reduced both tumor volume and tumor weight in mice (*P* < 0.001, **Figures 8A–C**). Meanwhile, no significant difference in body weight was observed among the groups (P > 0.05, **Figure 9**). H&E staining results showed that no obvious pathological damage, inflammatory cell infiltration, or abnormal tissue structure was observed in the heart, liver, spleen, and kidney tissues of the DEN-treated group, indicating that DEN has good *in vivo* biosafety within the effective antitumor dose range (**Figure 8D**). DEN also significantly inhibited the JAK-STAT3 signaling pathway (*P* < 0.01, **Figure 8E**) and regulated tumor inflammatory cytokines, as evidenced by the decrease in IL-6 levels (*P* < 0.05, **Figure 8F**) and the increase in TNF-α levels (*P* < 0.05, **Figure 8G**). This confirms that DEN can also effectively target and inhibit the hyperactivation of the JAK-STAT3 signaling pathway, while regulating the levels of inflammatory factors in the tumor microenvironment *in vivo*. Serum AST, ALT, ALP activity were significantly decreased after DEN treatment (*P* < 0.05, **Figures 8H–J**), while there was no significant change in Cr, BUN content (*P >* 0.05, **Figures 8K, L**). The above results further indicate that DEN has no obvious toxic damage to the liver and kidneys of mice.

## 4 Discussion

ATC is characterized by high invasiveness and poor prognosis, with conventional therapies often demonstrating limited efficacy (14). Dendrobine, an active alkaloid isolated from the traditional Chinese medicinal herb *Dendrobium*, exhibits a favorable safety profile and has demonstrated antitumor potential in lung cancer (15), colorectal cancer (16), and renal cell carcinoma (17). The present study explored the inhibitory impacts of dendrobine on the proliferation, invasion, and migration of ATC cells, and first applied Dendrobine to the treatment of subcutaneous tumors in mice, aimed at elucidating its functional mechanism through targeting the JAK-STAT3 pathway.

This study preliminarily confirmed the significant inhibitory effect of dendrobine on the viability of ATC cells *in vitro*, with the optimal effect at 5 μM. This result is consistent with findings by Kim et al. (10), indicating that 5 μM dendrobine significantly inhibits A549 cell viability. Our data further indicate that dendrobine significantly reduced the proliferation and colony formation ability of ATC cells and induced apoptosis. The IC_50_ values in 8050C and CAL-62 cells were 5.54 μM and 5.29 μM, respectively. The study by Luo et al. (16) reported an IC_50_ value of 5.769 μM for dendrobine in colorectal cancer cells, effectively suppressing their proliferation, which was highly consistent with our findings. Previous studies also corroborate that natural compounds with anticancer properties typically exhibit IC_50_ values within the low micromolar concentration range (18, 19). Consistently, dendrobine treatment significantly reduced tumor volume and weight in mouse model. H&E staining further revealed decreased tumor malignancy after dendrobine intervention. Research by Li et al. (15) indicates that dendrobine can inhibit tumor tissue growth and exert an anti-tumor effect *in vivo*, in agreement with the results of this study.

Due to its high invasiveness and insensitivity to radiotherapy and chemotherapy, ATC demonstrates poor clinical treatment outcomes. Inhibiting migration is critical for preventing ATC metastasis (20). This study conducted Transwell and wound healing assays, which demonstrated significantly decreased invasive and migratory capacities of ATC cells following dendrobine treatment. These findings suggest that dendrobine may impede tumor progression by suppressing cellular migration, which is consistent with previous studies on its role in colon cancer (16). Similar anti-migratory effects have been observed in studies investigating plant-derived compounds such as curcumin (21) and berberine (22) in cancer therapy. The above studies collectively support the potential application of dendrobine as a natural alkaloid compound in anti-cancer treatment. EMT is a critical process through which tumor cells acquire invasive and metastatic properties (23). E-cad serves as a key molecule mediating intercellular adhesion in epithelial cells, with its loss frequently associated with EMT in various cancers (24). N-cad functions as a transmembrane adhesion protein and established EMT marker (25), while Vim has been confirmed to critically regulate tumor initiation and progression via EMT modulation (26). Based on these mechanisms, immunofluorescence assays revealed that dendrobine upregulated E-cad expression while downregulating N-cad and Vim levels. This indicates that dendrobine may exert its anti-invasive and anti-migratory effects through EMT inhibition. The current findings highlight the therapeutic potential of dendrobine in cancer treatment.

The JAK/STAT pathway is a signal transduction cascade activated by various cytokines. STAT3 is an oncogene that is known to promote cancer cell proliferation, motility, progression, and survival (27). Predominantly localized in the cytoplasm, STAT3 becomes phosphorylated (p-STAT3) via JAK-mediated activation upon cytokine stimulation, subsequently translocating to the nucleus to regulate apoptosis and proliferation. Hyperactivated STAT3 enhances cancer cell proliferation, metastasis, and induces chemoresistance, establishing it as a key therapeutic target for inhibiting these processes (28). Based on this, we focused on the JAK-STAT3 signaling pathway. WB analysis revealed that dendrobine treatment significantly reduced phosphorylation levels of JAK1, JAK2, and STAT3. These findings indicate that dendrobine exerts anticancer effects by inhibiting activation of the JAK-STAT3 signaling pathway. Li et al. (29) reported that dendrobine may treat metabolic fatty liver disease by modulating STAT3 and its phosphorylation levels. Although this study is not related to cancer, it supports the findings of the present study that dendrobine exerts anti-ATC effects through the regulation of STAT3-related pathways. Given that IL-6 is a key activator of STAT3 signaling transduction and tumor metastasis (30), we pretreated ATC cells with IL-6 to evaluate its potential to reverse dendrobine’s anticancer effects. Results showed that IL-6 partially restored proliferation and migration capacities in dendrobine-treated cells while also restoring phosphorylation levels of JAK1, JAK2, and STAT3. This suggests inflammatory microenvironments may compromise dendrobine’s therapeutic efficacy, implying future clinical applications may require combining dendrobine with anti-inflammatory therapies. Furthermore, STAT3-siRNA not only enhanced dendrobine-mediated suppression of JAK-STAT3 signaling but also synergistically inhibited ATC cell migration and invasion with dendrobine. Although dendrobine has been studied in other types of cancer, its mechanisms in ATC remain unclear, particularly its targeting of the JAK-STAT3 pathway and effects on EMT. The above findings confirm the critical role of the JAK-STAT3 pathway in the mechanism of dendrobine against ATC.

*In vivo* results indicated that dendrobine dose-dependently reduced tumor volume and weight while decreasing tumor malignancy. Notably, dendrobine decreased intratumoral IL-6 levels and elevated TNF-α levels, and its regulatory effects on the JAK/STAT3 pathway aligned with findings from the *in vitro* studies. As IL-6 promotes tumor cell proliferation and inhibits apoptosis through the JAK/STAT3 pathway (31), dendrobine-mediated IL-6 reduction may block sustained STAT3 activation, thereby suppressing tumor growth. Concurrently, increased TNF-α likely enhances T-cell activity to counteract tumor immune evasion (32). These findings suggest dendrobine exerts both direct antitumor effects and indirect therapeutic benefits by modulating inflammatory responses in the tumor microenvironment. In addition, dendrobine treatment reduced hepatic AST, ALT and ALP activities in mice serum, suggesting its potential to improve liver function indirectly through tumor growth inhibition. While dendrobine did not significantly alter serum kidney parameters, indicating the absence of notable toxic effects and supporting its safety as a potential anticancer agent. In this study, dendrobine was administered at doses of 10 and 40 mg/kg, demonstrating lower toxicity compared to other known STAT3 inhibitors such as ODZ10117 (1 mg/kg) and SG-1721 (0.5 mg/kg) (33). Additionally, most synthetic inhibitors target only STAT3 or a single JAK isoform (34), whereas dendrobine inhibits the p-JAK1, p-JAK2, and p-STAT3 simultaneously. Its combined anti-inflammatory effects may effectively overcome therapeutic resistance in clinical ATC patients, offering significant potential for improving their prognosis.

Although the results of the present study support the low toxicity and safety profile of moderate dendrobine doses (10 and 40 mg/kg) in animals, other studies have highlighted potential adverse effects of dendrobine at higher doses (100 and 200 mg/kg), including hepatotoxicity, nephrotoxicity, and neurotoxicity (7). Additional studies have indicated that intravenous administration of 2 mg/kg dendrobine may exacerbate toxicity through interactions with other compounds (35), particularly those involving cytochrome P450 enzymes, as dendrobine is primarily metabolized via this pathway (36). Thus, further preclinical pharmacodynamic studies are required to define the safe dose range of dendrobine, balancing its therapeutic efficacy with toxicity risks. Moreover, as a lipophilic alkaloid, dendrobine exhibits relatively rapid metabolism, resulting in a low absolute oral bioavailability of approximately 2.32% (37). However, this value is comparable to clinically used natural anticancer drugs such as paclitaxel, which has an absolute oral bioavailability of about 1.3% (38). Existing studies have explored formulation strategies such as nanoparticle drug delivery systems to improve the bioavailability of dendrobine in animals and address the above pharmacokinetic limitations (39). Currently, no studies have comprehensively evaluated dendrobine’s safety profile in humans. Patients with hepatic or renal dysfunction should consult medical professionals before dendrobine administration and receive individualized dosing based on hepatic and renal function tests.

Although this study has achieved some significant findings, several limitations should be acknowledged. For instance, experiments were primarily conducted at cellular level and in animal models without validation in clinical specimens. Therefore, future studies should incorporate clinical samples from ATC patients. We also did not investigate the therapeutic effects of combining dendrobine with other chemotherapeutic or targeted drugs. Future research should further explore the synergistic effects between dendrobine and other agents, as well as employ high-throughput sequencing technologies to elucidate more detailed molecular mechanisms. Additionally, we plan to employ molecular docking and network pharmacology approaches in future studies to confirm the direct effects of dendrobine on pathway-related factors. Experiments related to how dendrobine affects cell growth and proliferation, as well as more detailed safety evaluations of dendrobine in cells and animals, should be further improved in subsequent studies. Investigating the toxicity range of dendrobine on normal thyroid cells and conducting pharmacokinetic studies in animals will provide important theoretical support for its future clinical application.

## 5 Conclusion

In conclusion, this study confirmed the inhibitory effect of dendrobine on the proliferation, invasion and migration of ATC cells through *in vivo* and *in vitro* experiments, while revealing its mechanism of action through targeting the JAK-STAT3 signaling pathway.

## Data Availability

The original contributions presented in the study are included in the article/Supplementary Material. Further inquiries can be directed to the corresponding author.
