# Supplementary material for: Dendrobine inhibits anaplastic thyroid cancer progression by targeting the JAK-STAT3 pathway
Source: Front Oncol. 2026 Jun 2;16:1842670. doi: 10.3389/fonc.2026.1842670 (PMC13268948; doi:10.3389/fonc.2026.1842670)
Supplement: Supplementary file 1 [file DataSheet1.zip › Supplementary Material/Fig5-WB/Note.docx]

Notes:

The leftmost band in all WB images within this folder is the protein ladder. The group order from left to right is as follows: Control group, 5 μM DEN group, IL-6 group and 5 μM DEN+IL-6 group. Image titles indicate the corresponding protein names.
