# Supplementary figures and images for: Dendrobine inhibits anaplastic thyroid cancer progression by targeting the JAK-STAT3 pathway
Source: Front Oncol. 2026 Jun 2;16:1842670. doi: 10.3389/fonc.2026.1842670 (PMC13268948; doi:10.3389/fonc.2026.1842670)

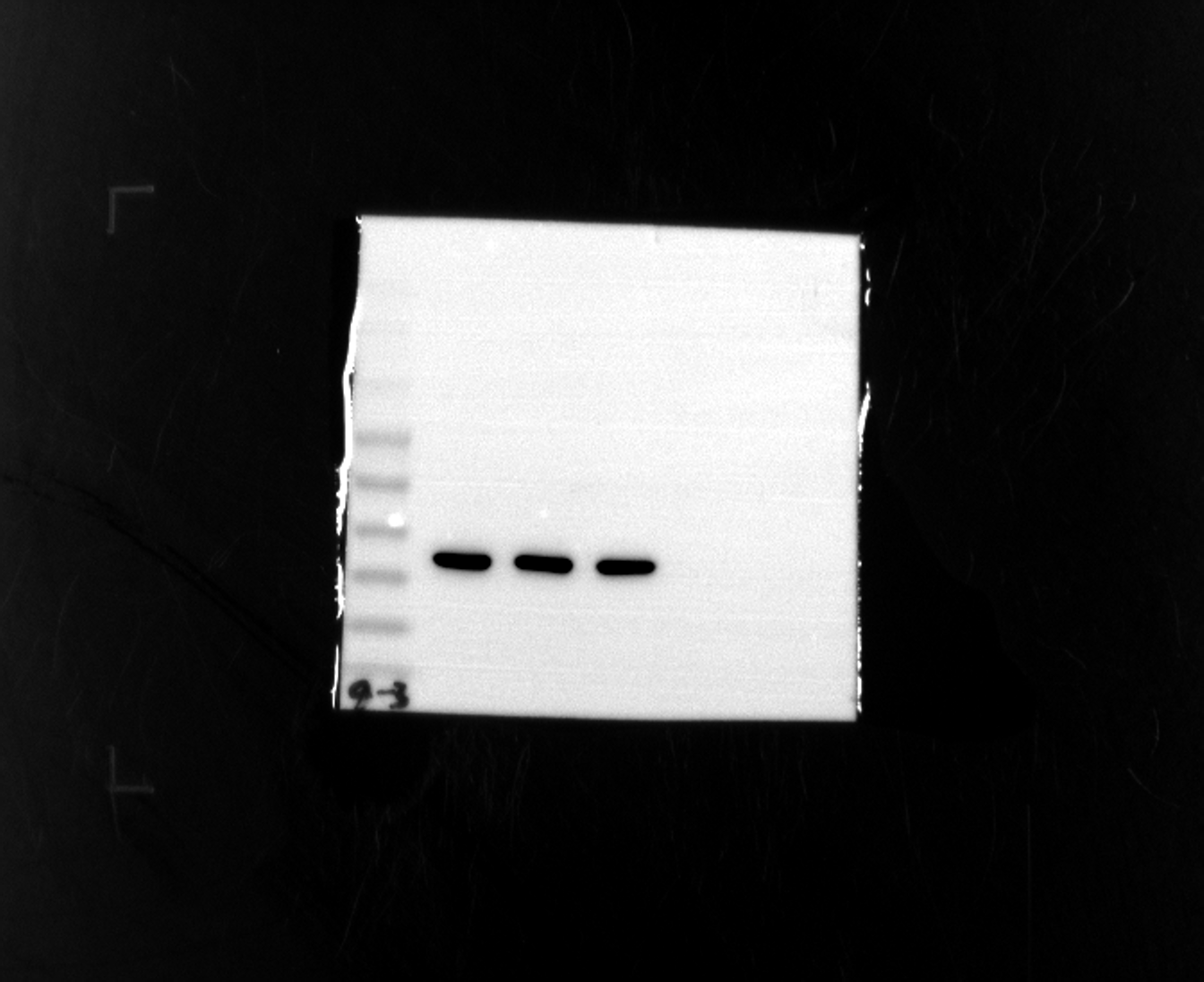

Supplement: Supplementary file 1 [file DataSheet1.zip › Supplementary Material/Fig4-WB/8505C/GAPDH.Tif]

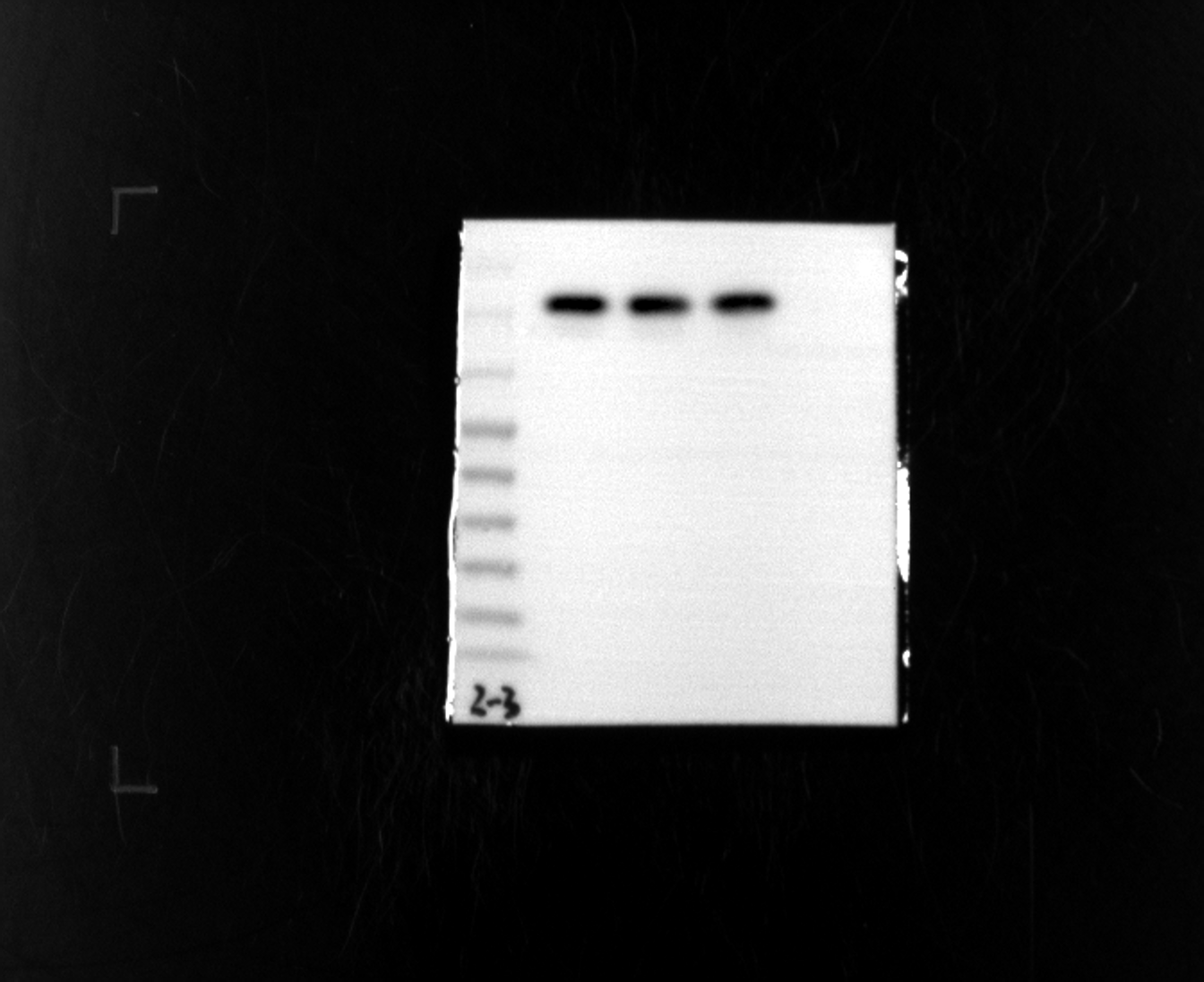

Supplement: Supplementary file 1 [file DataSheet1.zip › Supplementary Material/Fig4-WB/8505C/JAK1.Tif]

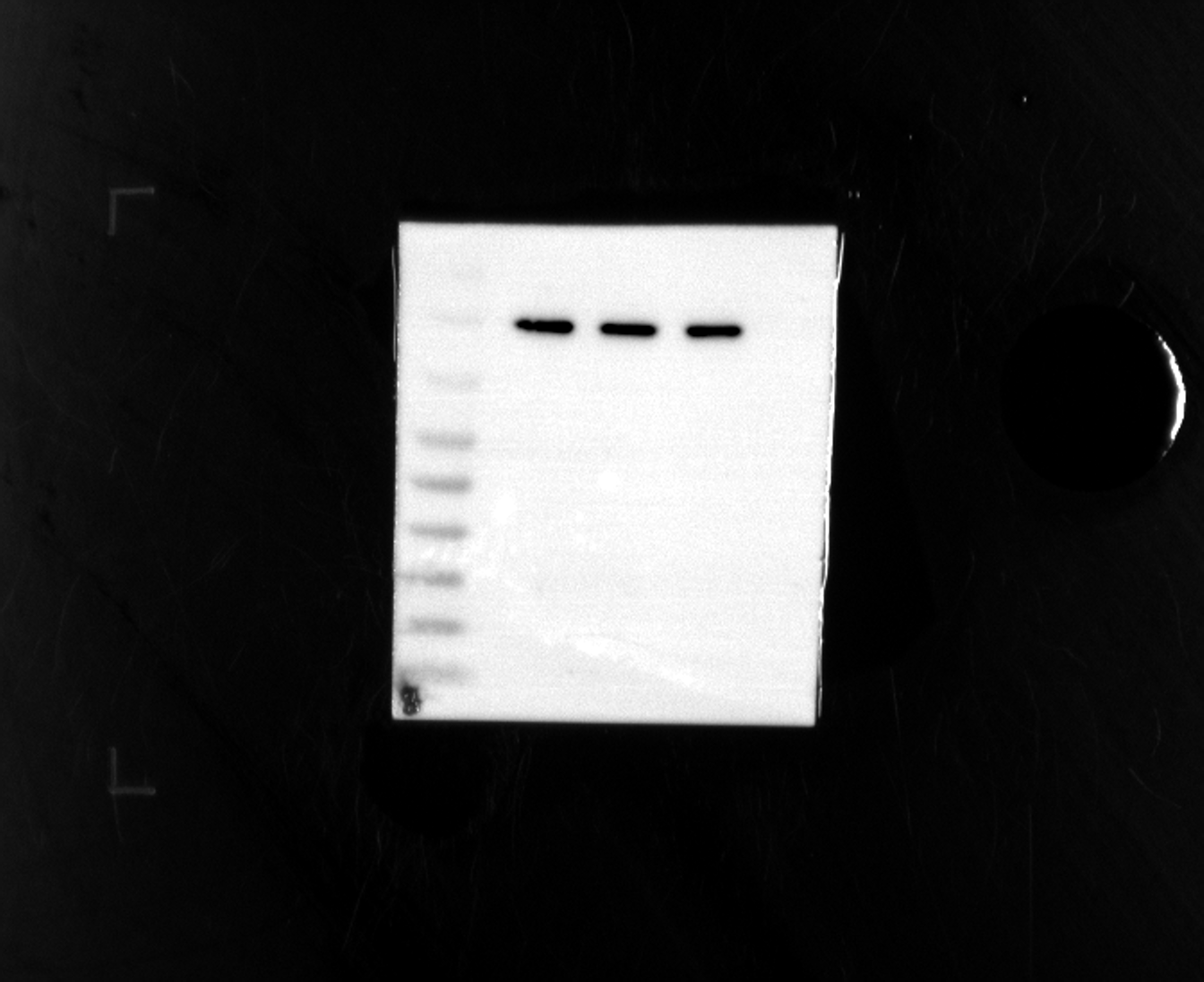

Supplement: Supplementary file 1 [file DataSheet1.zip › Supplementary Material/Fig4-WB/8505C/JAK2.Tif]

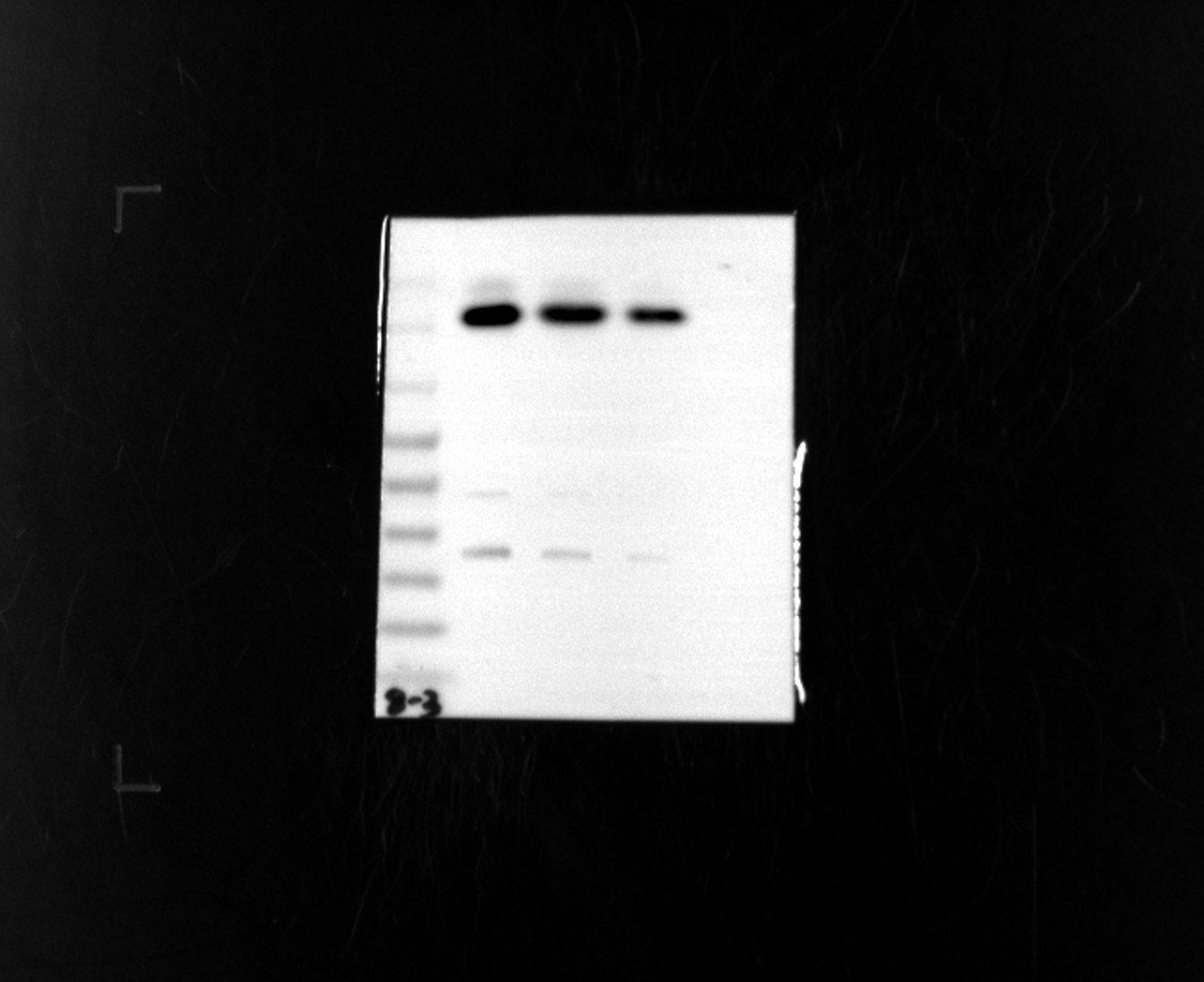

Supplement: Supplementary file 1 [file DataSheet1.zip › Supplementary Material/Fig4-WB/8505C/p-JAK1.Tif]

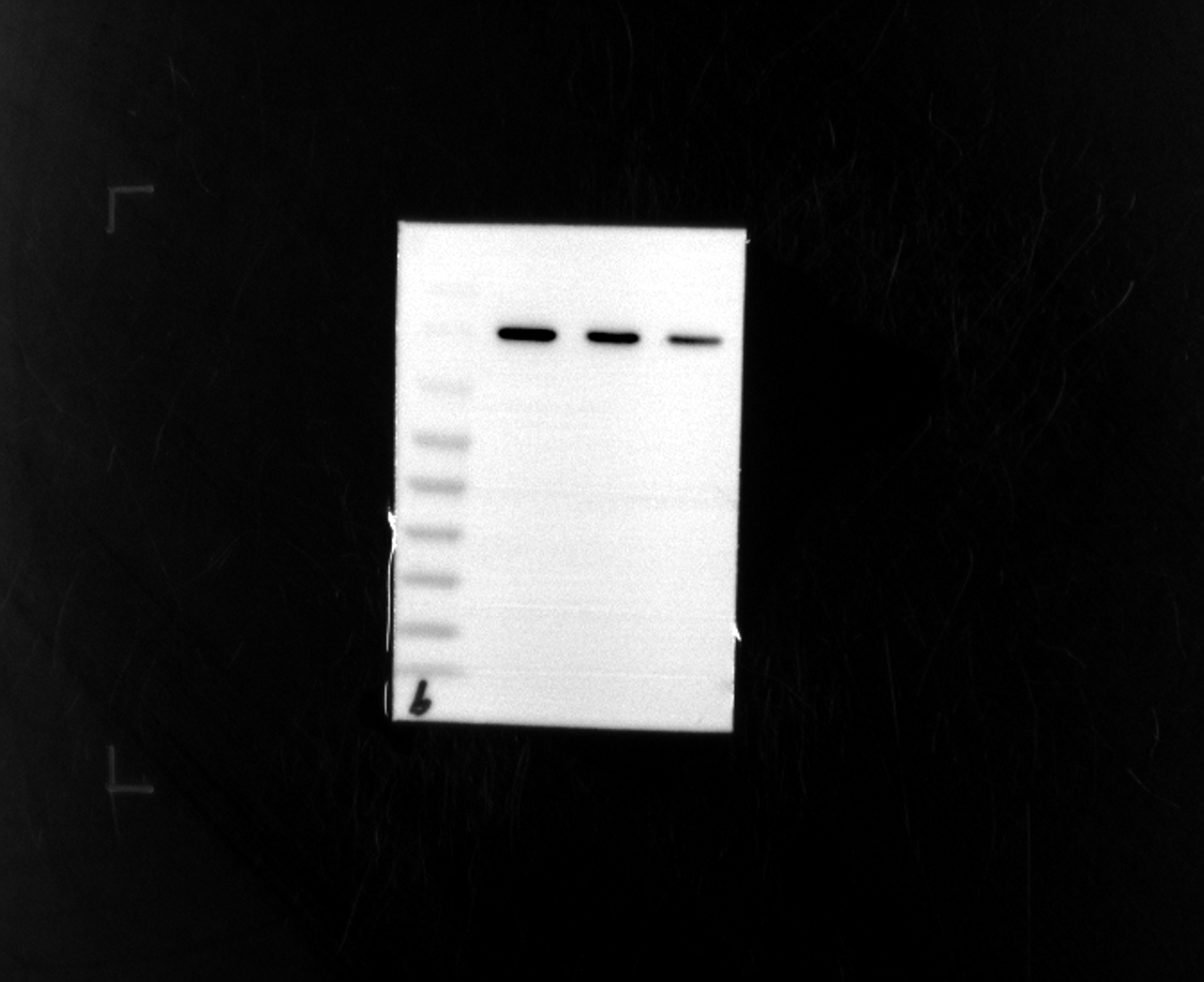

Supplement: Supplementary file 1 [file DataSheet1.zip › Supplementary Material/Fig4-WB/8505C/p-JAK2.Tif]

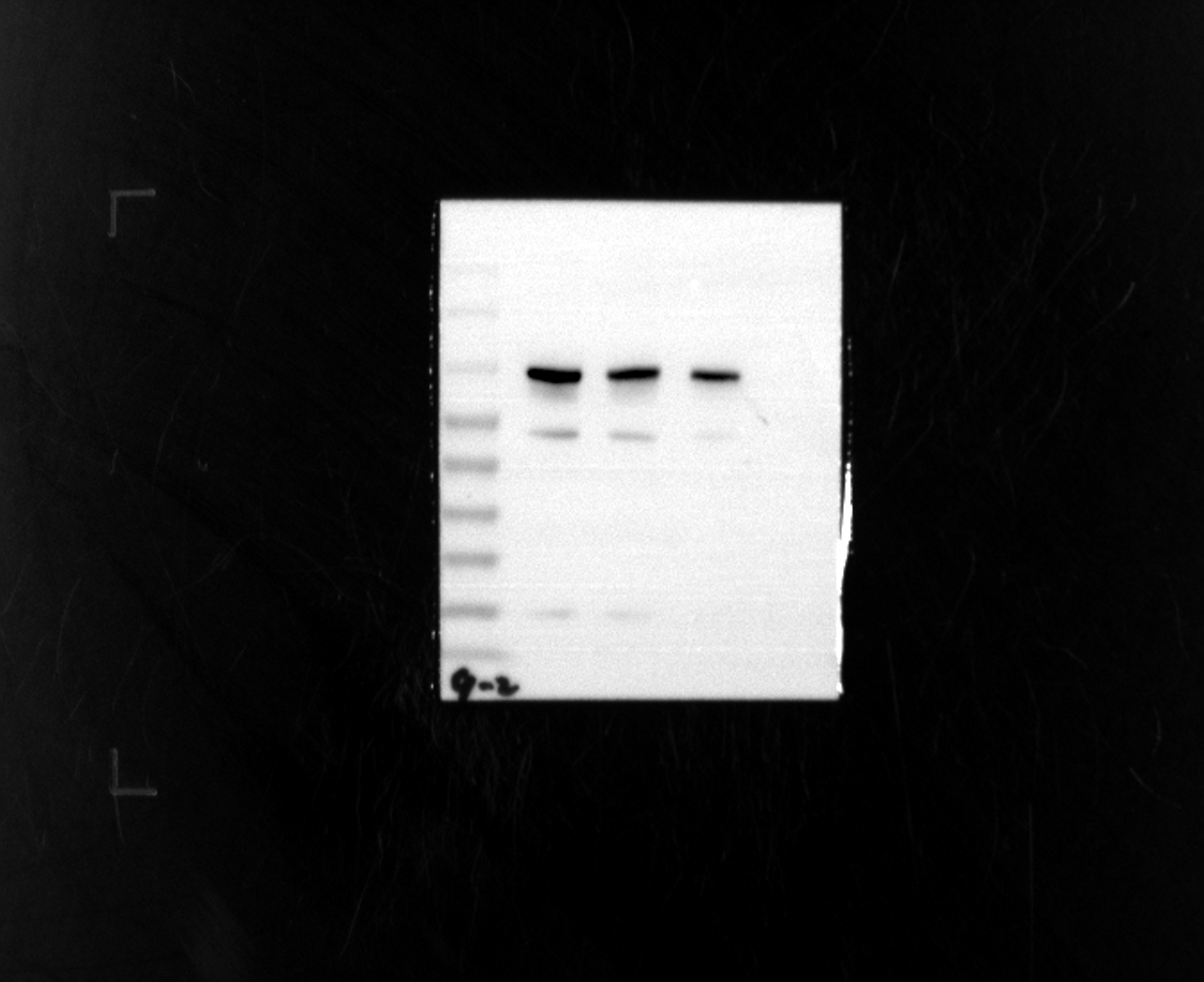

Supplement: Supplementary file 1 [file DataSheet1.zip › Supplementary Material/Fig4-WB/8505C/p-STAT3.Tif]

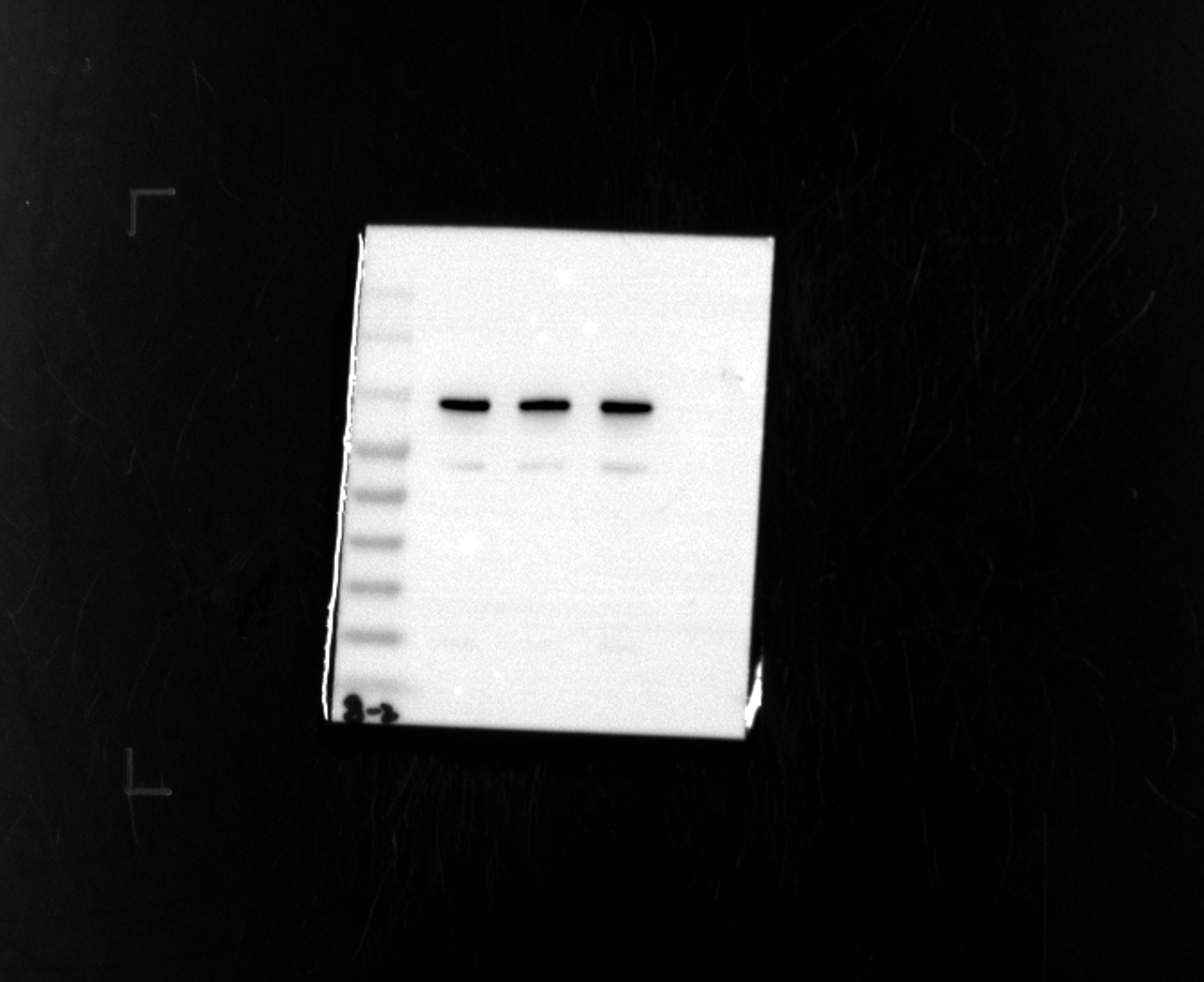

Supplement: Supplementary file 1 [file DataSheet1.zip › Supplementary Material/Fig4-WB/8505C/STAT3.Tif]

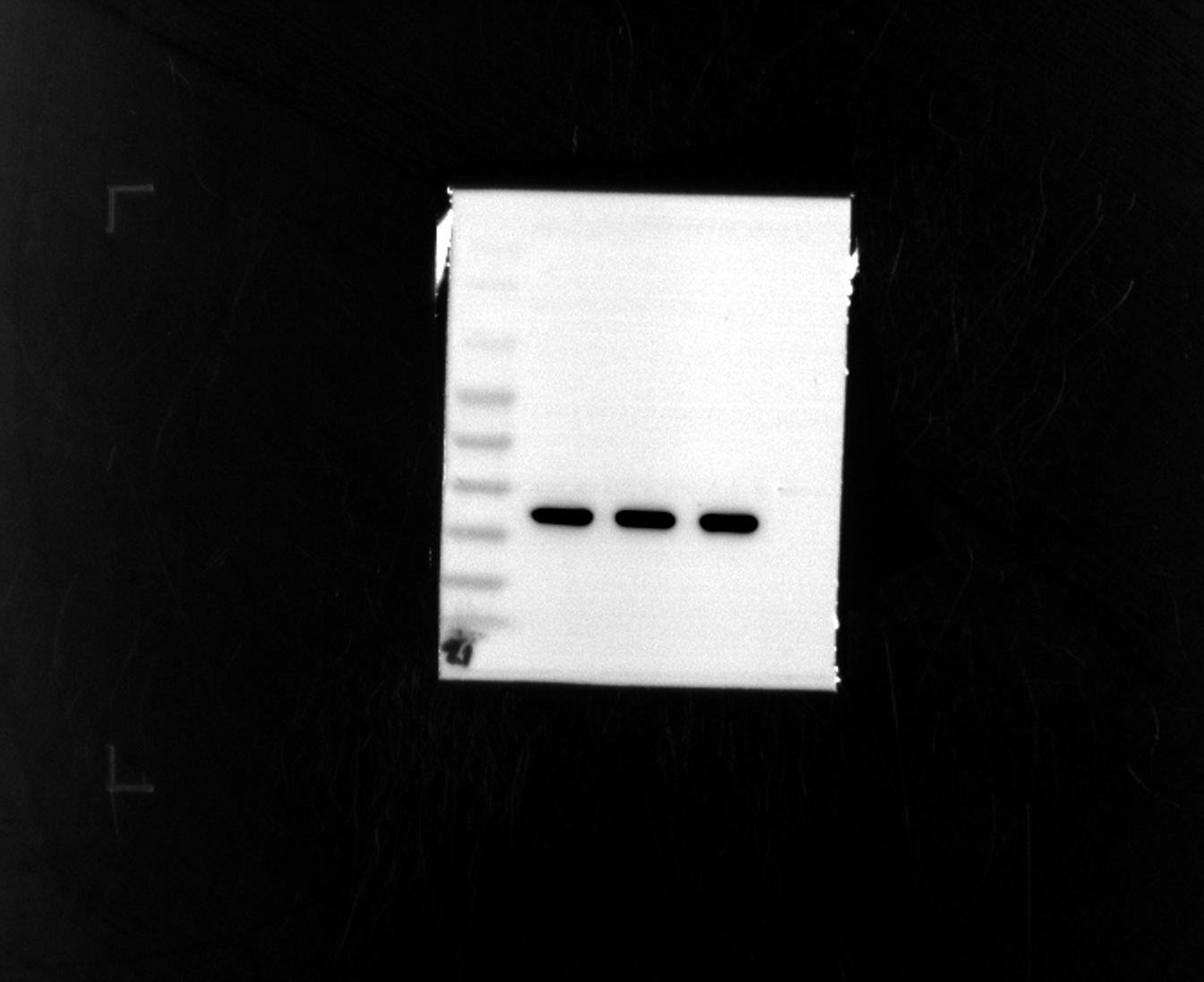

Supplement: Supplementary file 1 [file DataSheet1.zip › Supplementary Material/Fig4-WB/CAL-62/GAPDH.Tif]

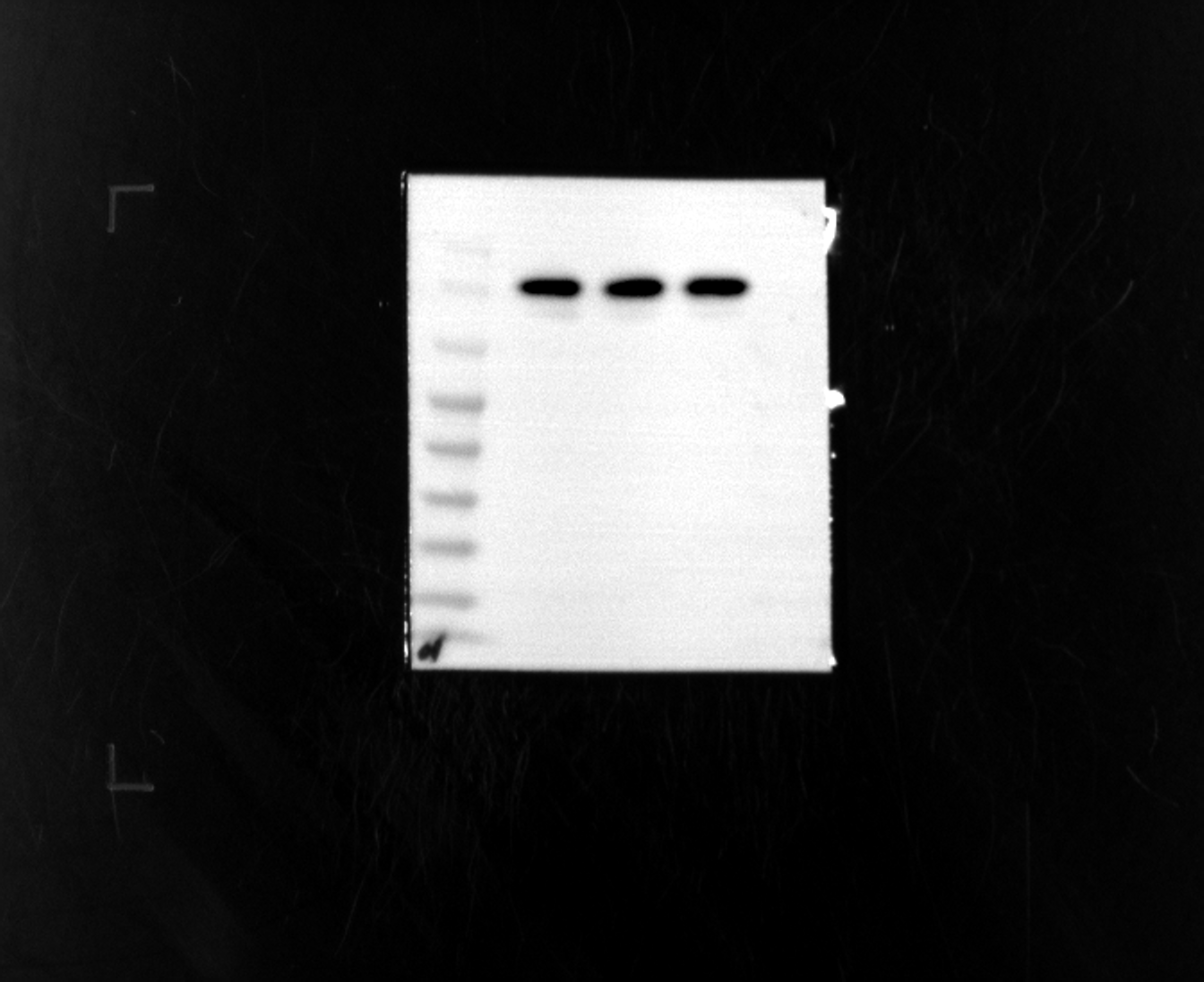

Supplement: Supplementary file 1 [file DataSheet1.zip › Supplementary Material/Fig4-WB/CAL-62/JAK1.Tif]

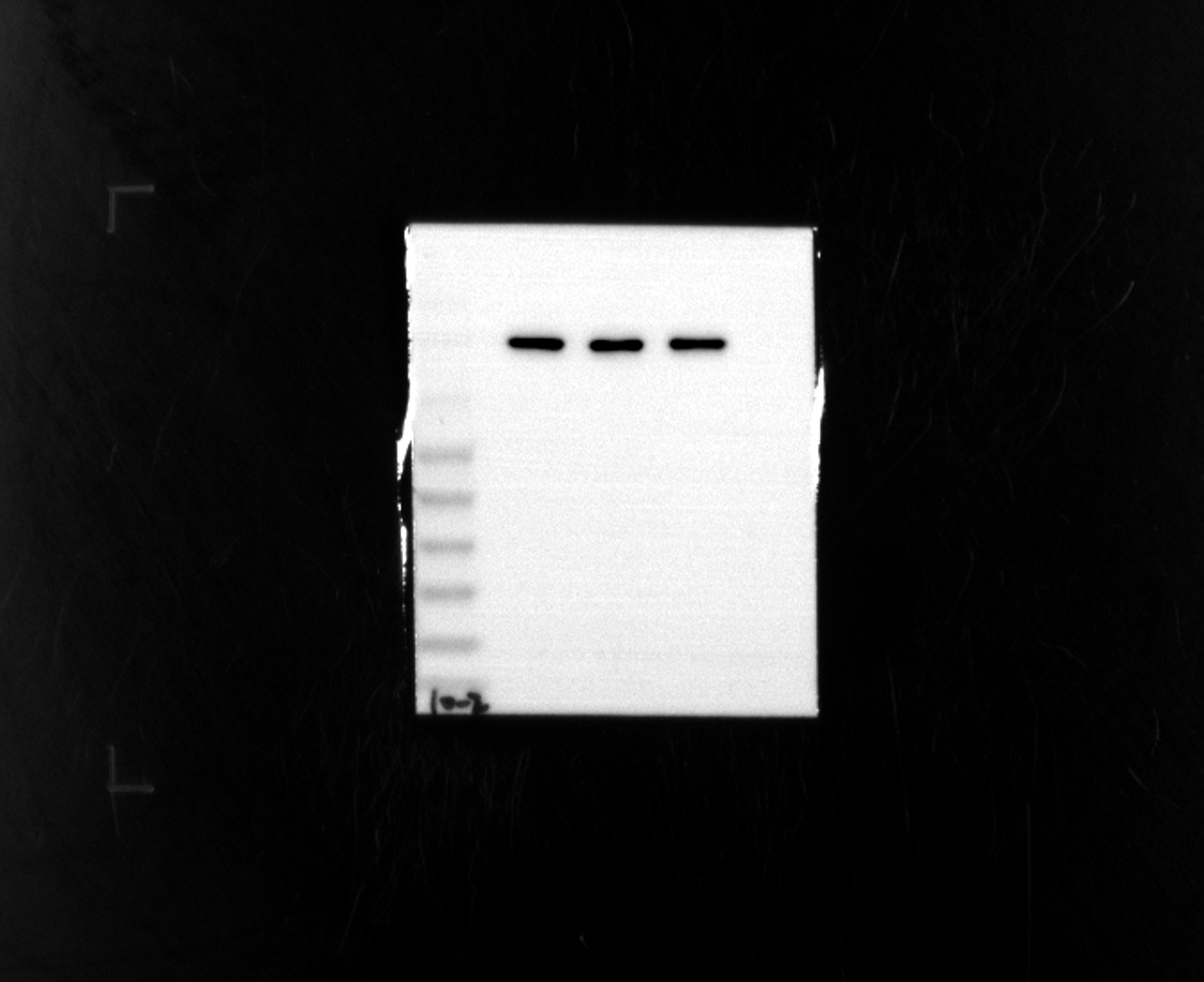

Supplement: Supplementary file 1 [file DataSheet1.zip › Supplementary Material/Fig4-WB/CAL-62/JAK2.Tif]

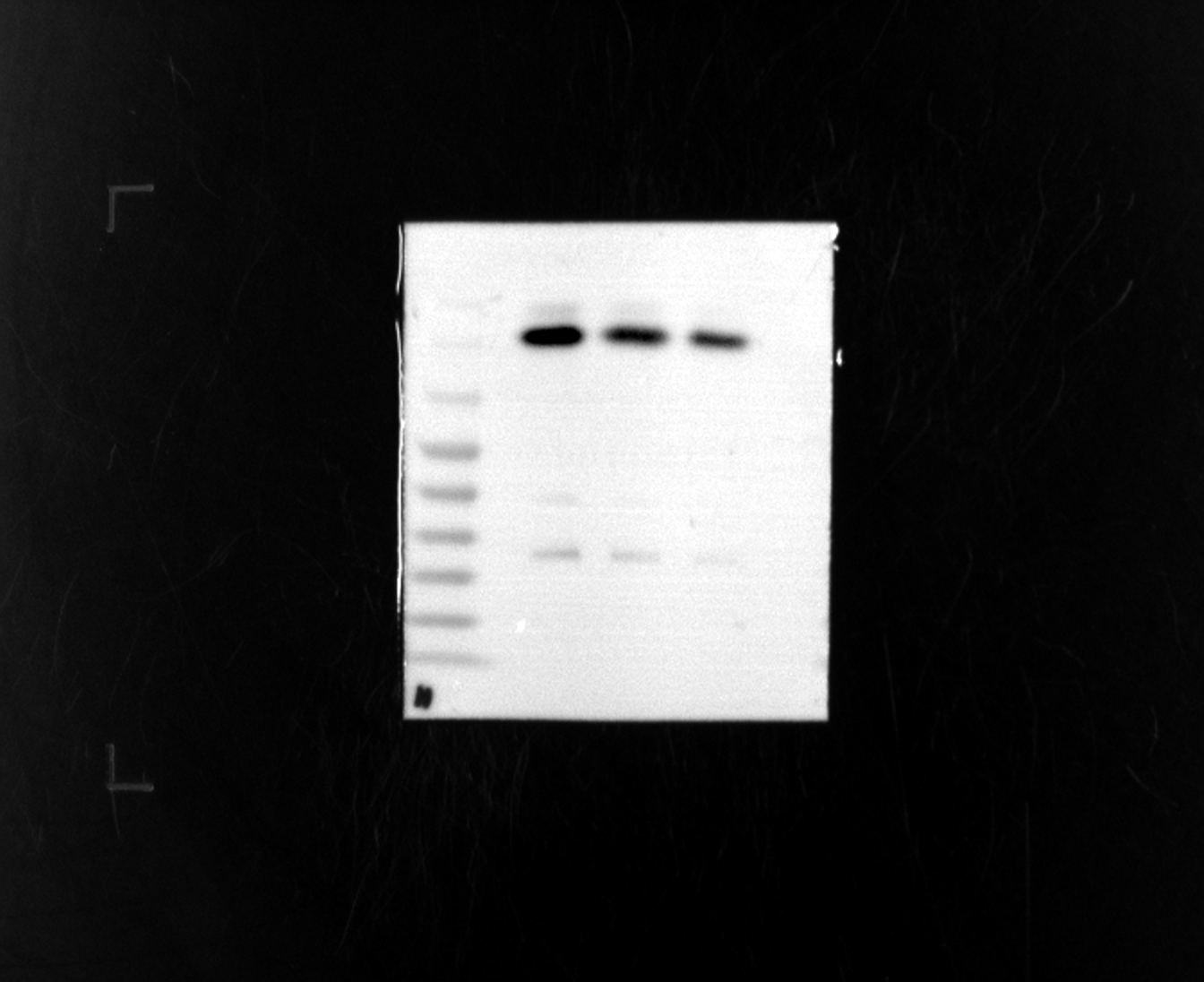

Supplement: Supplementary file 1 [file DataSheet1.zip › Supplementary Material/Fig4-WB/CAL-62/p-JAK1.Tif]

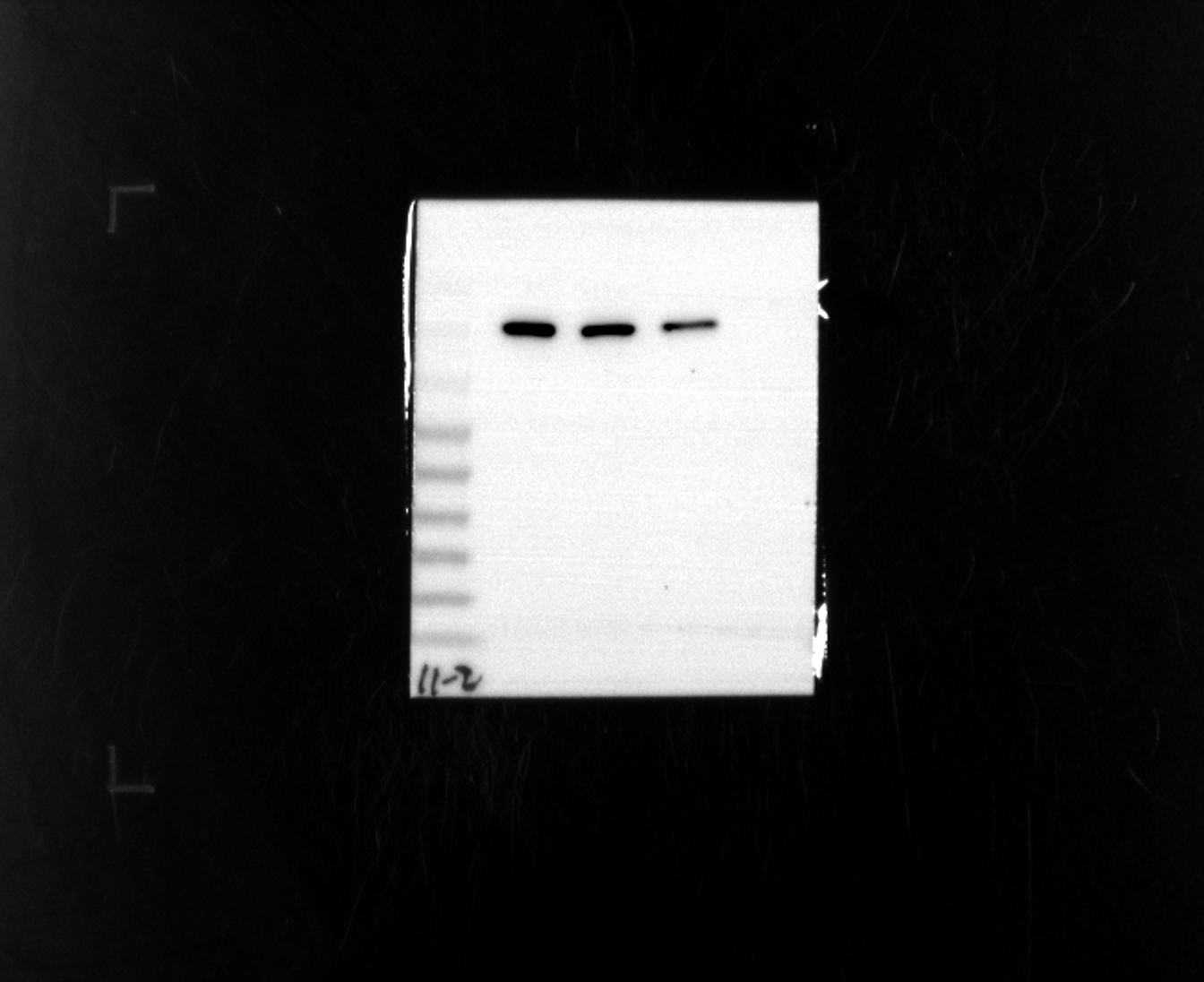

Supplement: Supplementary file 1 [file DataSheet1.zip › Supplementary Material/Fig4-WB/CAL-62/p-JAK2.Tif]

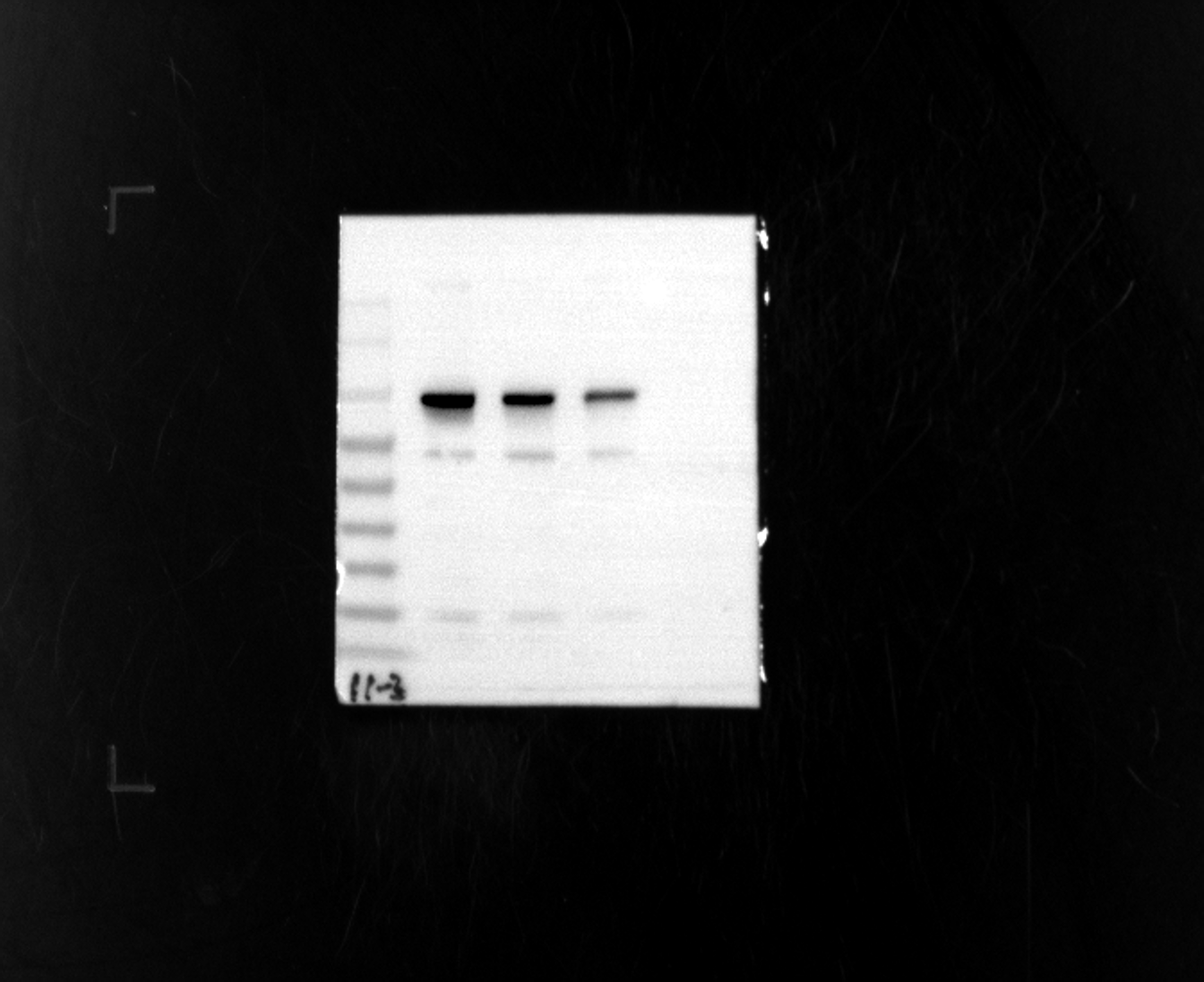

Supplement: Supplementary file 1 [file DataSheet1.zip › Supplementary Material/Fig4-WB/CAL-62/p-STAT3.Tif]

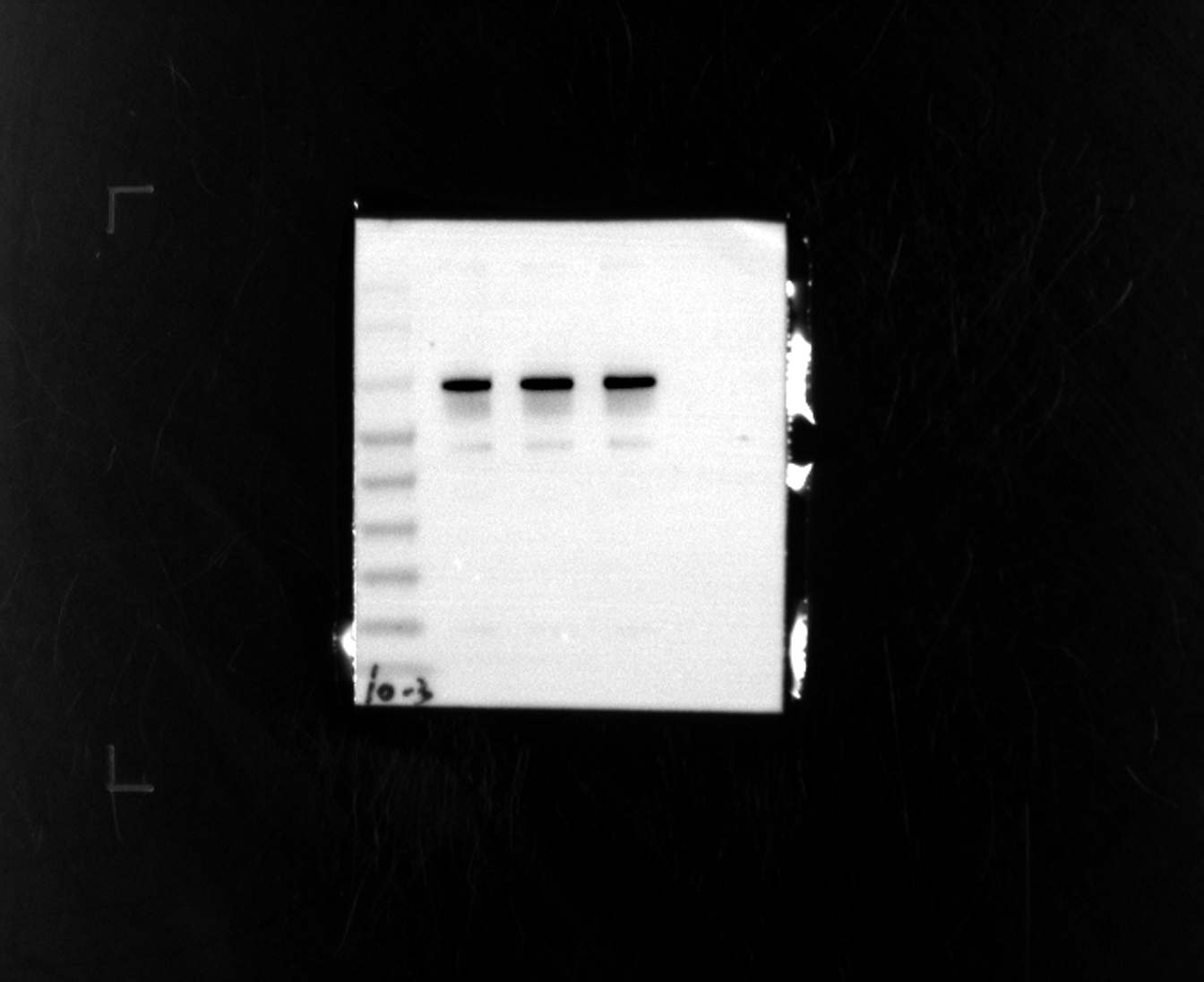

Supplement: Supplementary file 1 [file DataSheet1.zip › Supplementary Material/Fig4-WB/CAL-62/STAT3.Tif]

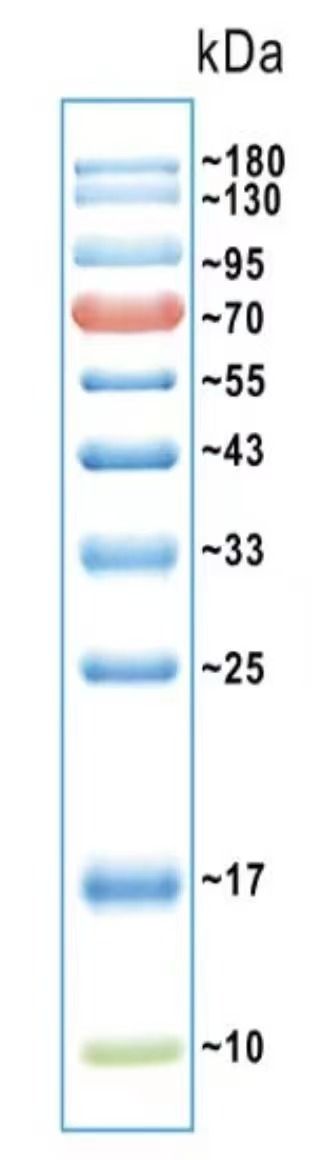

Supplement: Supplementary file 1 [file DataSheet1.zip › Supplementary Material/Fig4-WB/Marker.jpg]

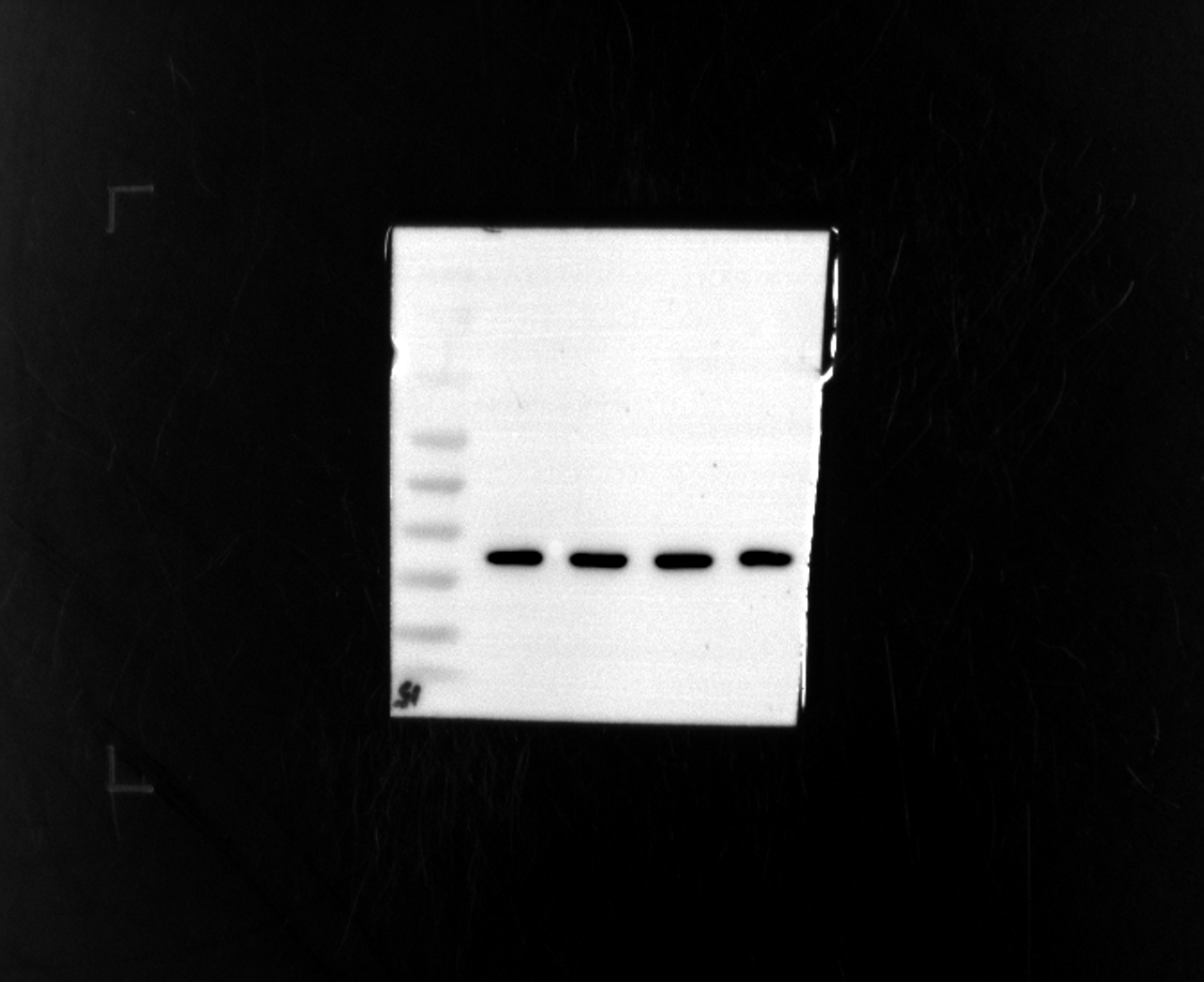

Supplement: Supplementary file 1 [file DataSheet1.zip › Supplementary Material/Fig5-WB/8505C/GAPDH.Tif]

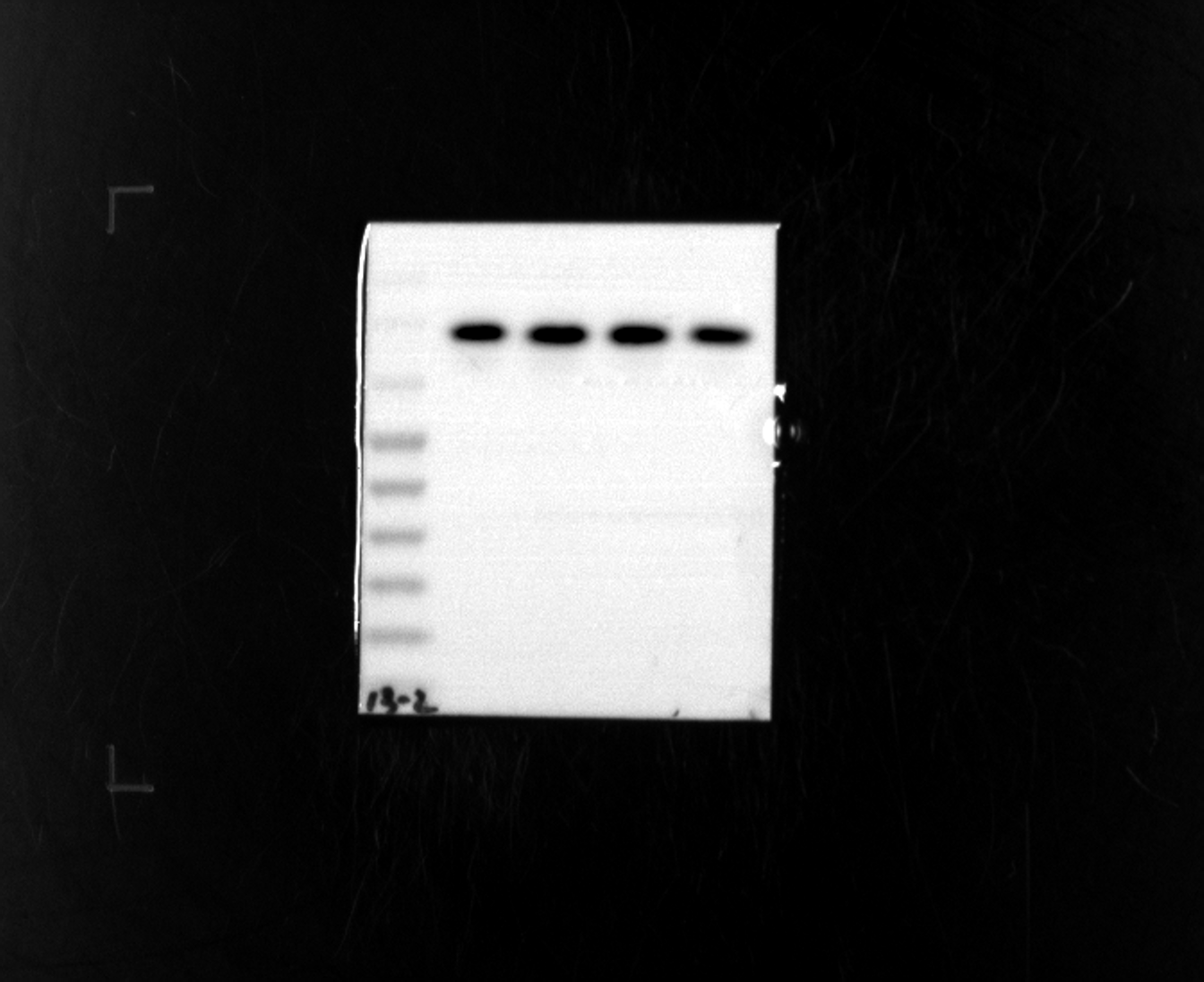

Supplement: Supplementary file 1 [file DataSheet1.zip › Supplementary Material/Fig5-WB/8505C/JAK1.Tif]

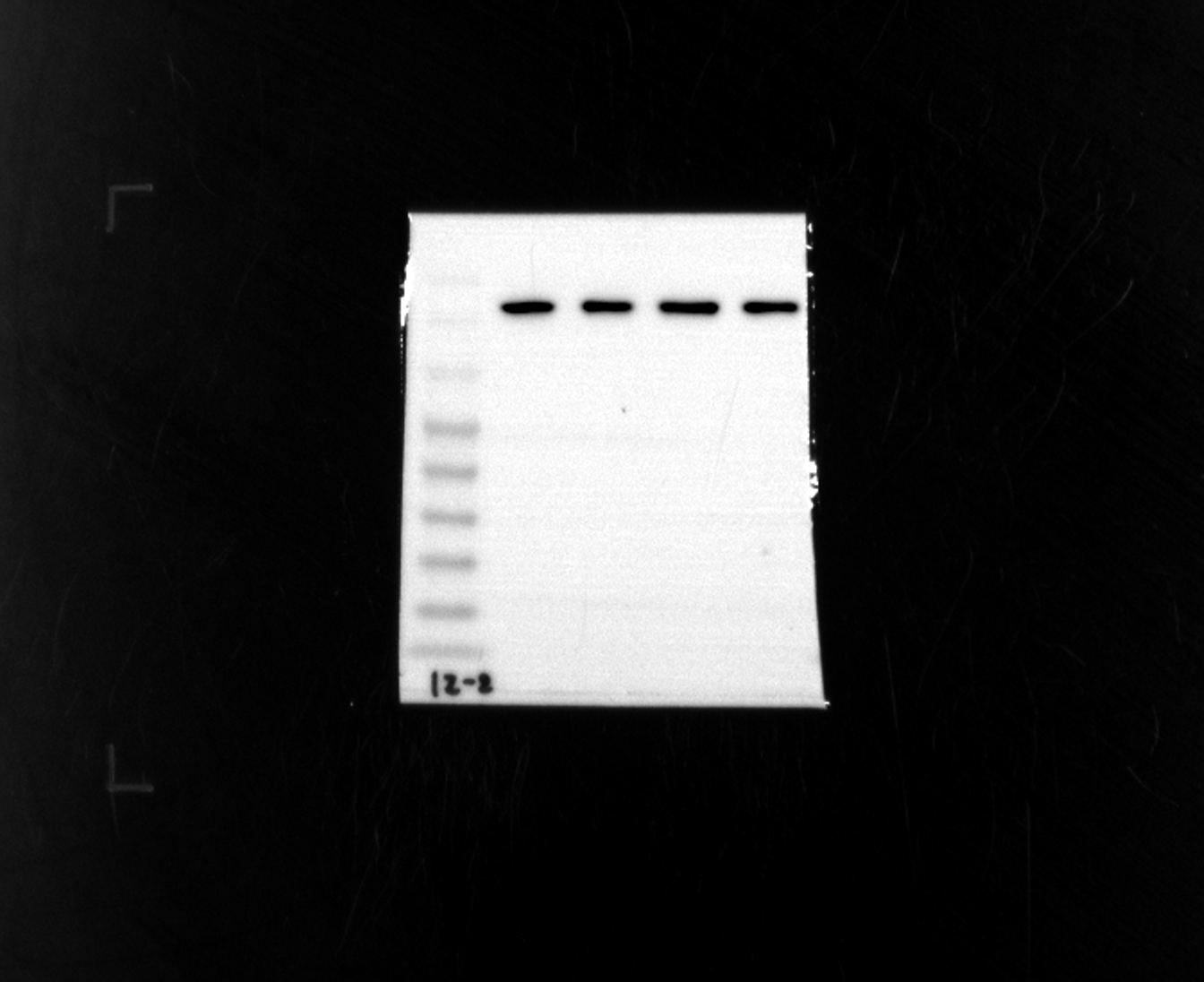

Supplement: Supplementary file 1 [file DataSheet1.zip › Supplementary Material/Fig5-WB/8505C/JAK2.Tif]

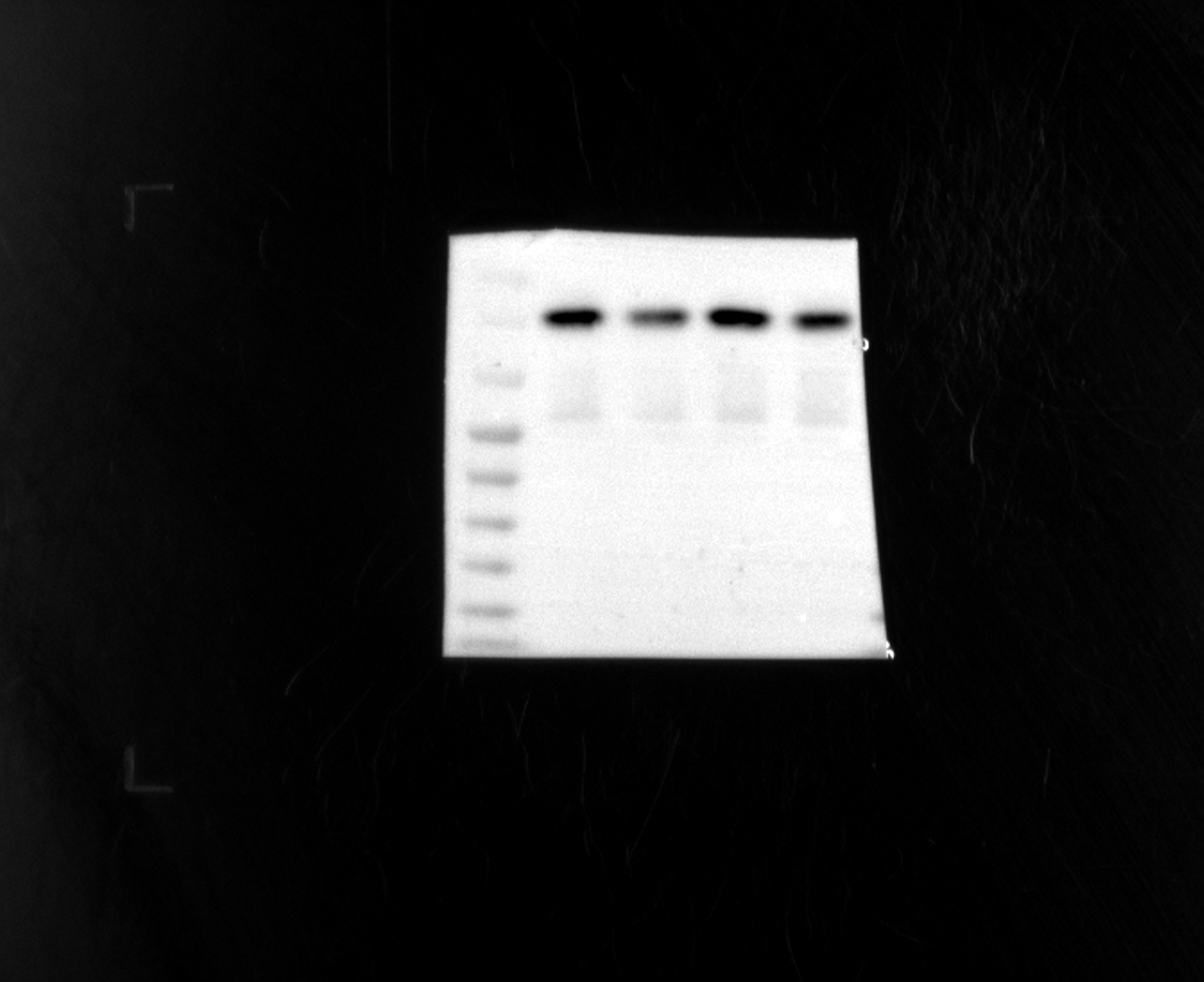

Supplement: Supplementary file 1 [file DataSheet1.zip › Supplementary Material/Fig5-WB/8505C/p-JAK1.Tif]

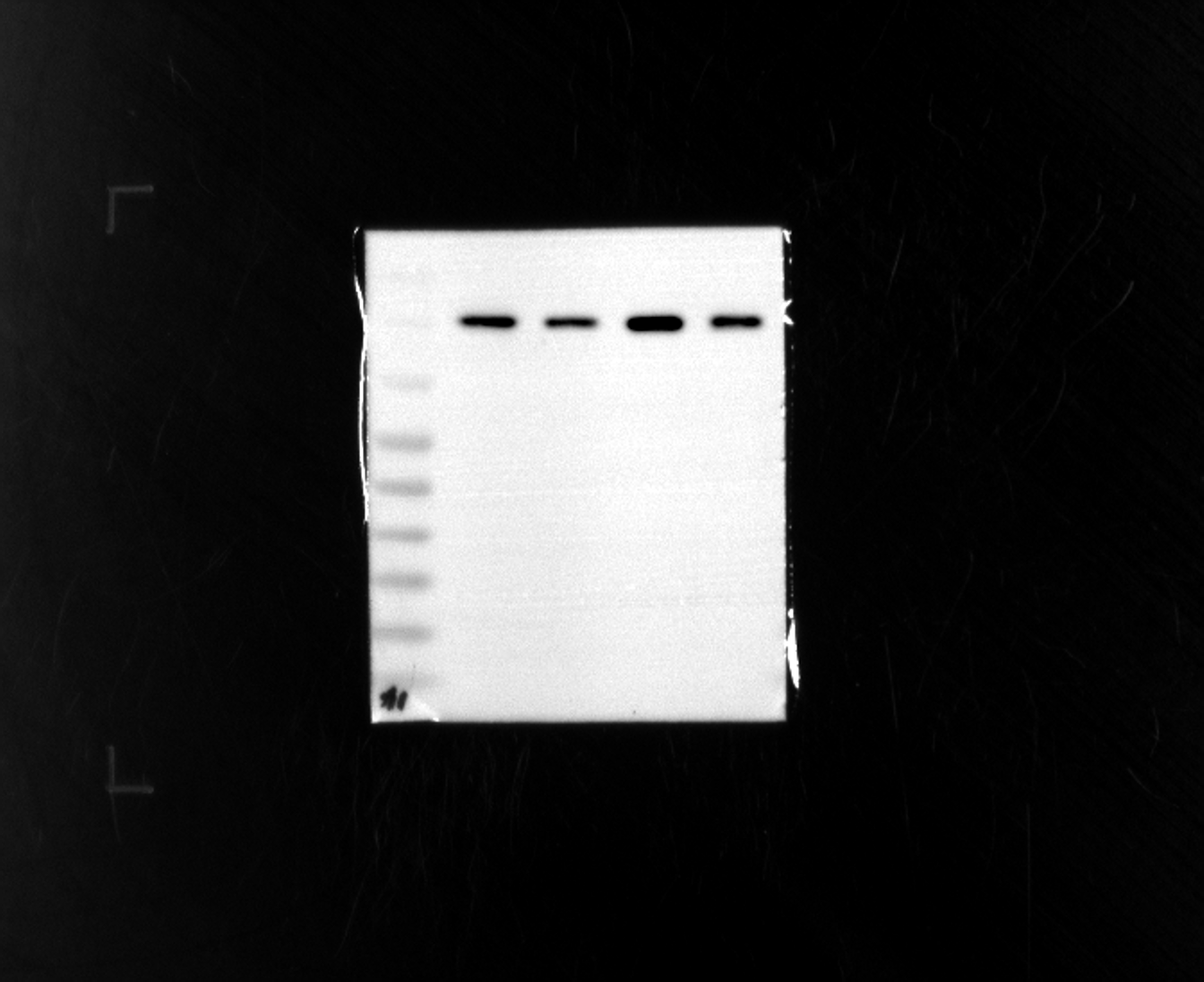

Supplement: Supplementary file 1 [file DataSheet1.zip › Supplementary Material/Fig5-WB/8505C/p-JAK2.Tif]

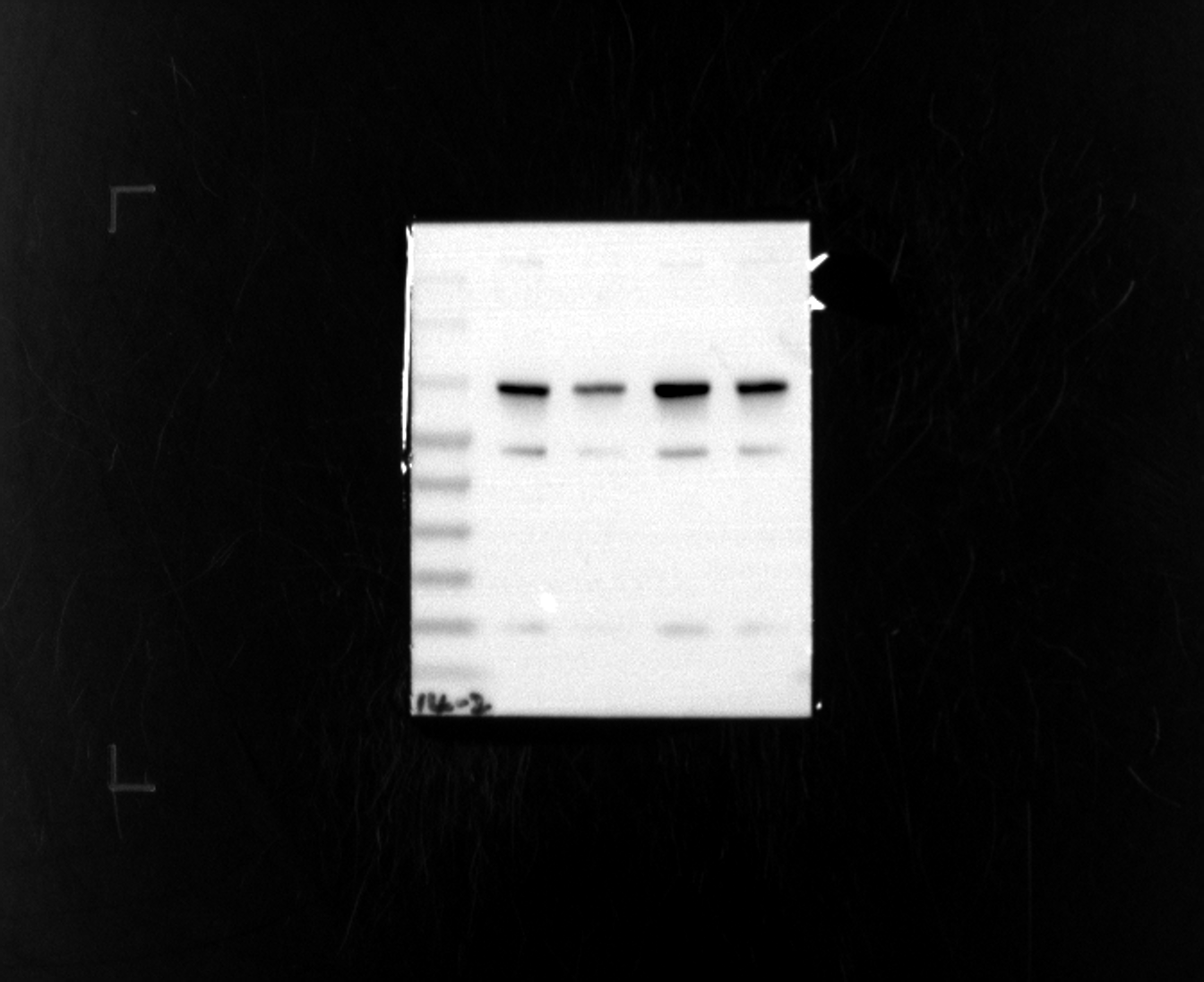

Supplement: Supplementary file 1 [file DataSheet1.zip › Supplementary Material/Fig5-WB/8505C/p-STAT3.Tif]

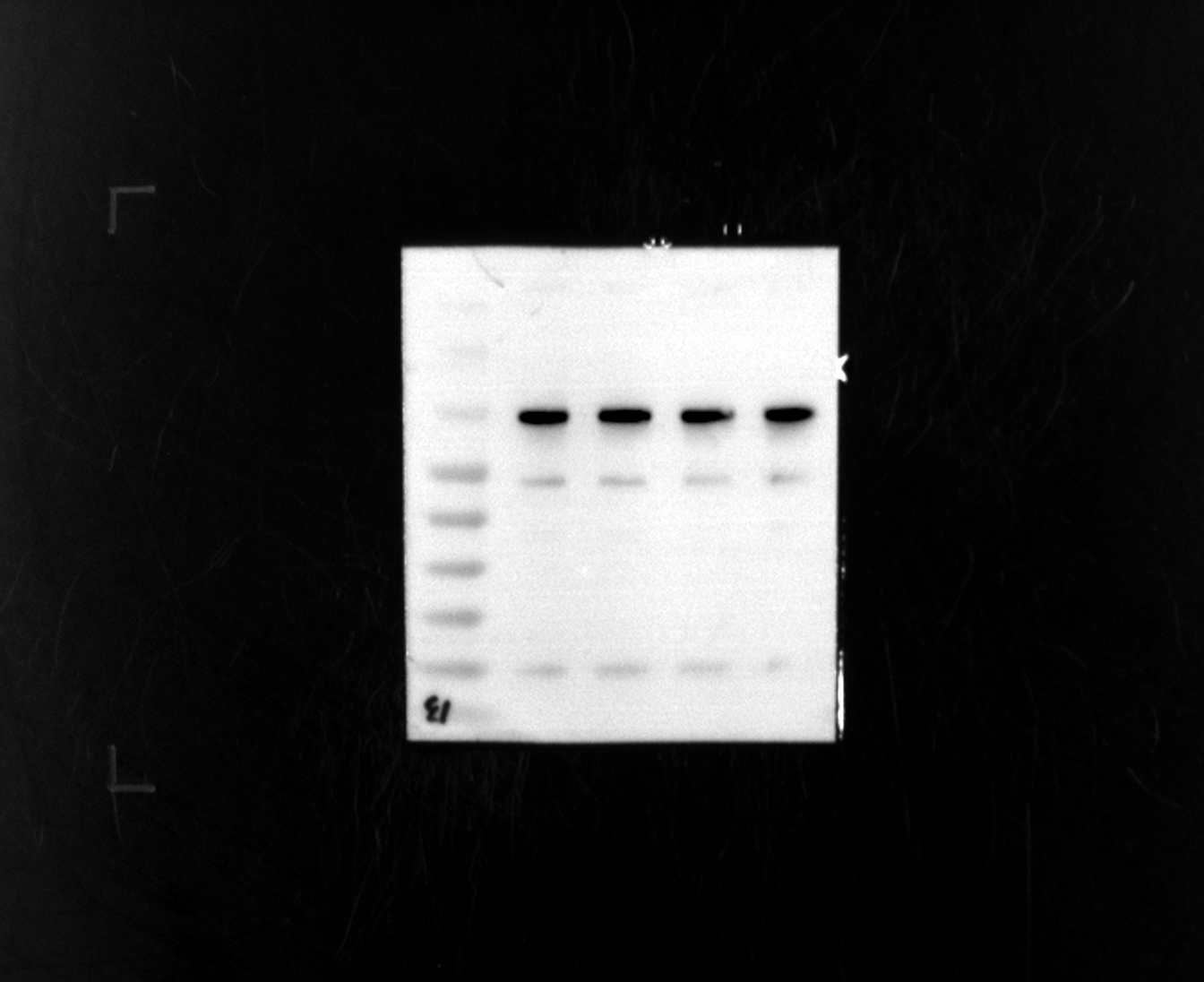

Supplement: Supplementary file 1 [file DataSheet1.zip › Supplementary Material/Fig5-WB/8505C/STAT3.Tif]

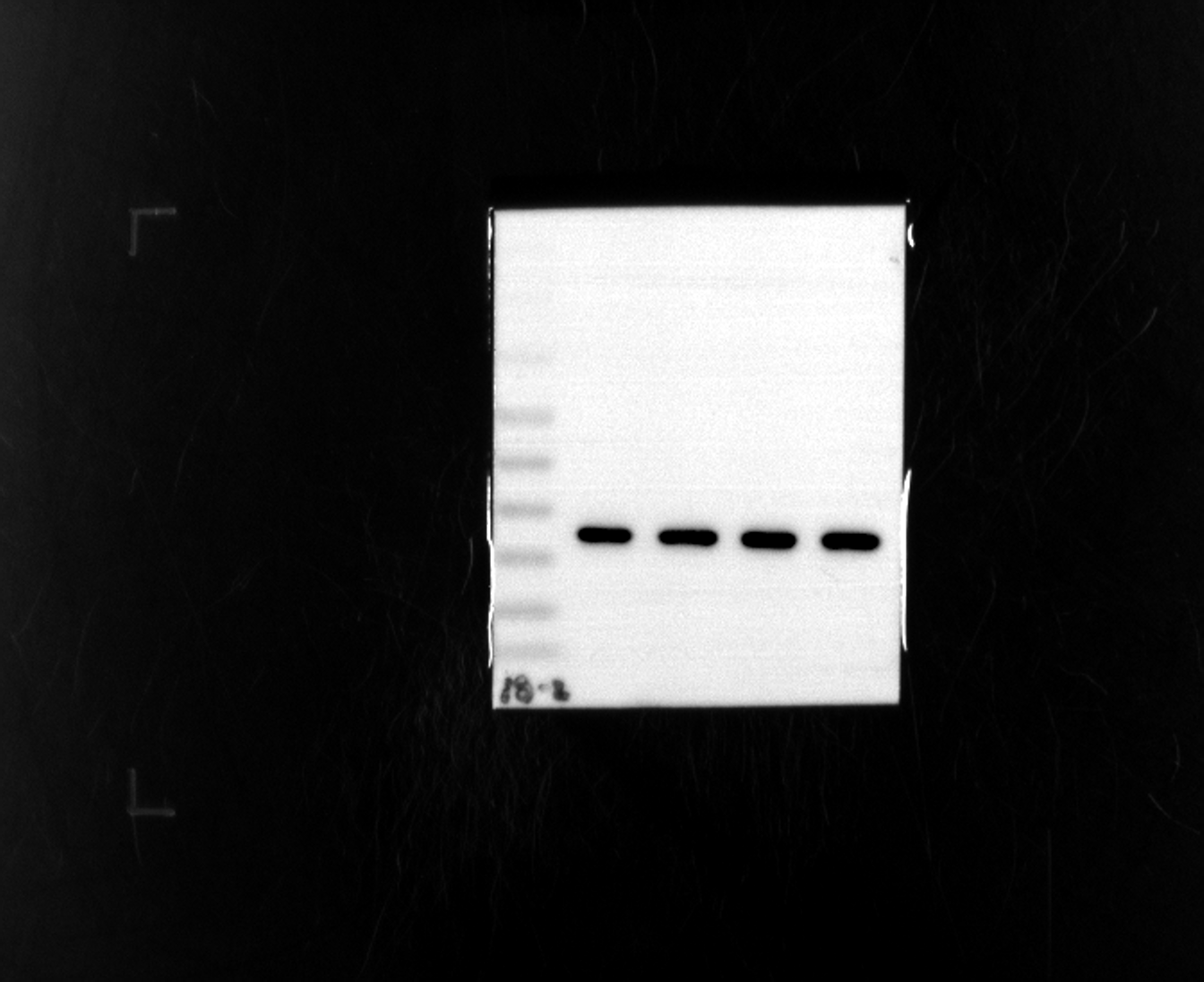

Supplement: Supplementary file 1 [file DataSheet1.zip › Supplementary Material/Fig5-WB/CAL-62/GAPDH.Tif]

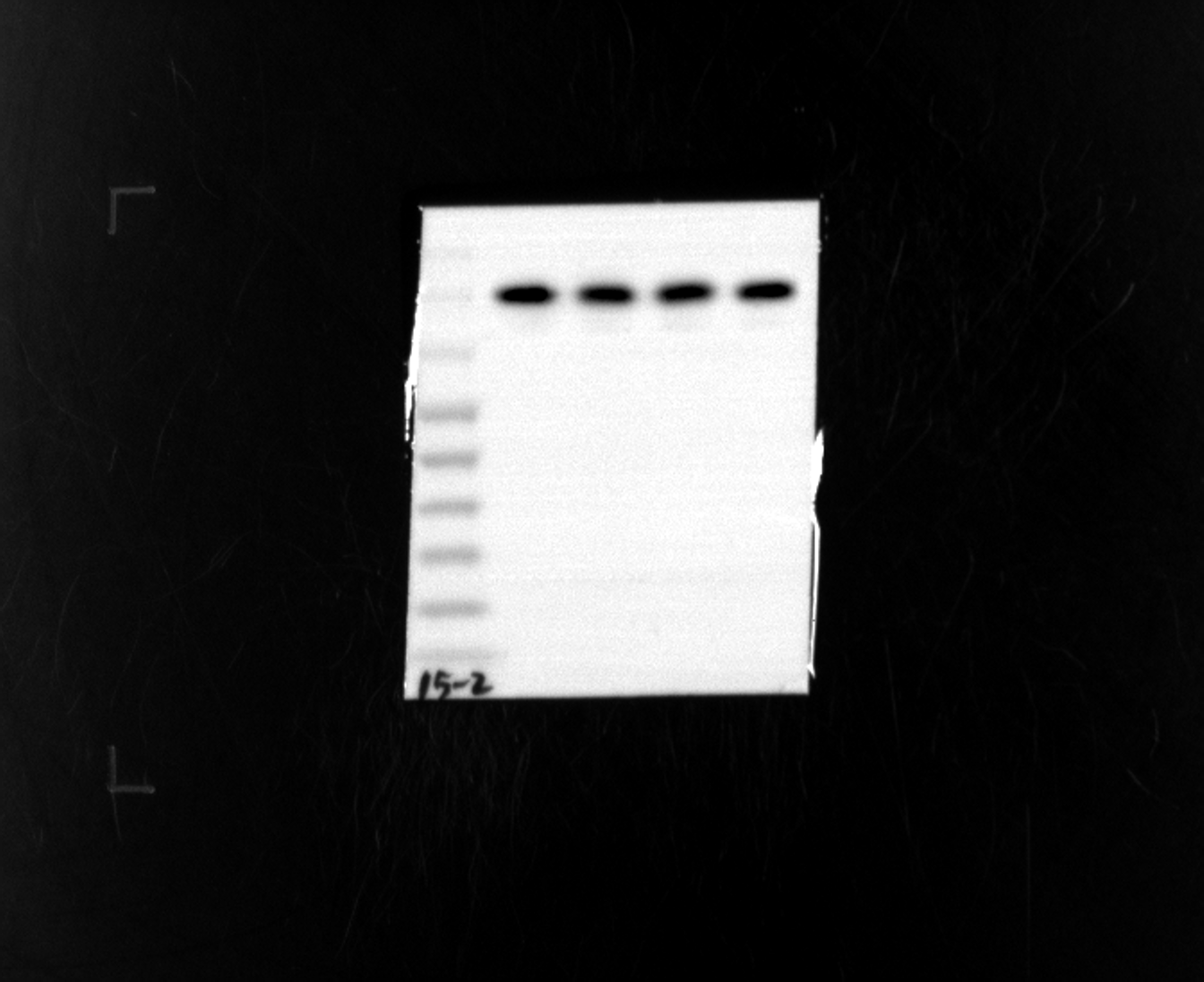

Supplement: Supplementary file 1 [file DataSheet1.zip › Supplementary Material/Fig5-WB/CAL-62/JAK1.Tif]

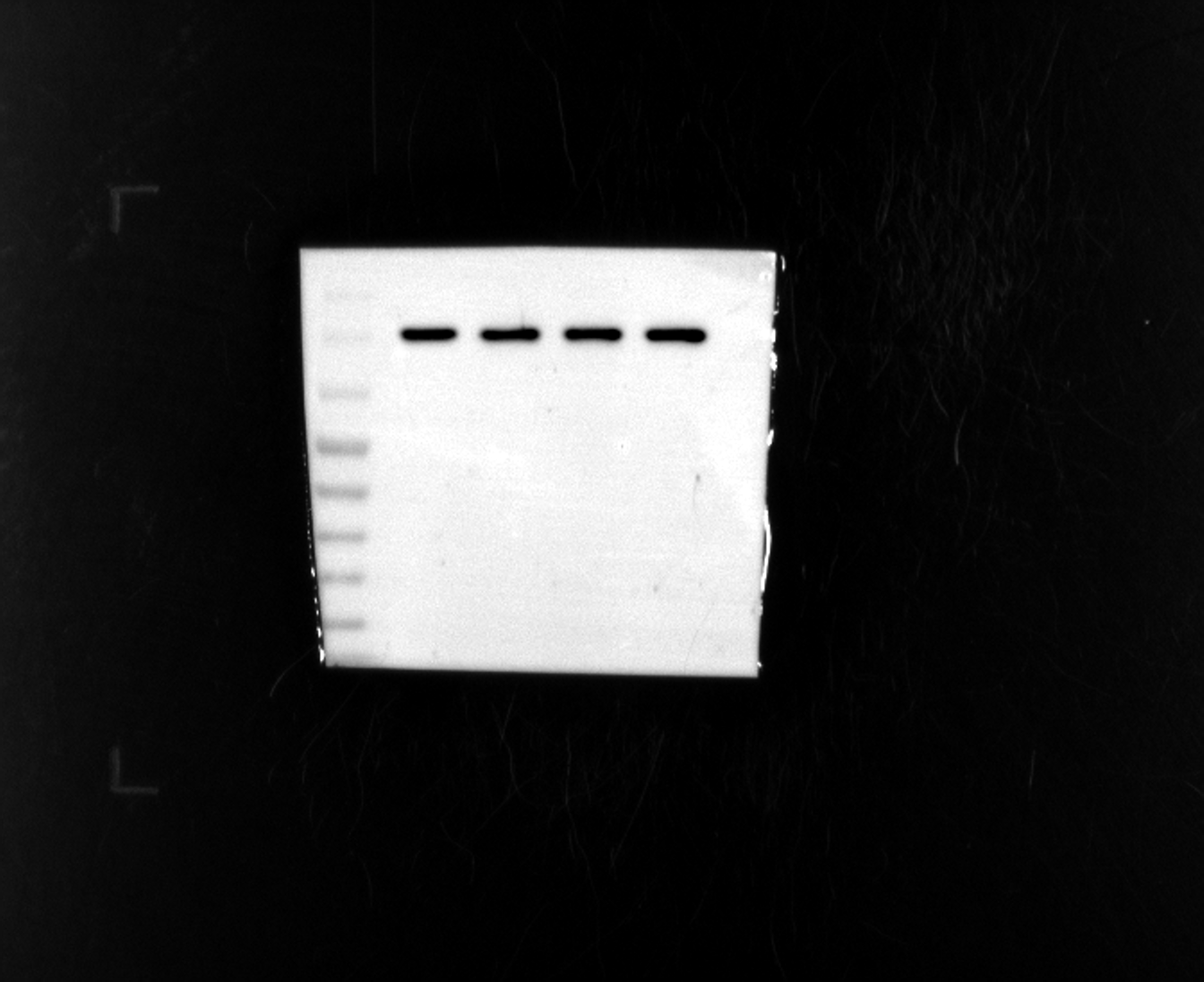

Supplement: Supplementary file 1 [file DataSheet1.zip › Supplementary Material/Fig5-WB/CAL-62/JAK2.Tif]

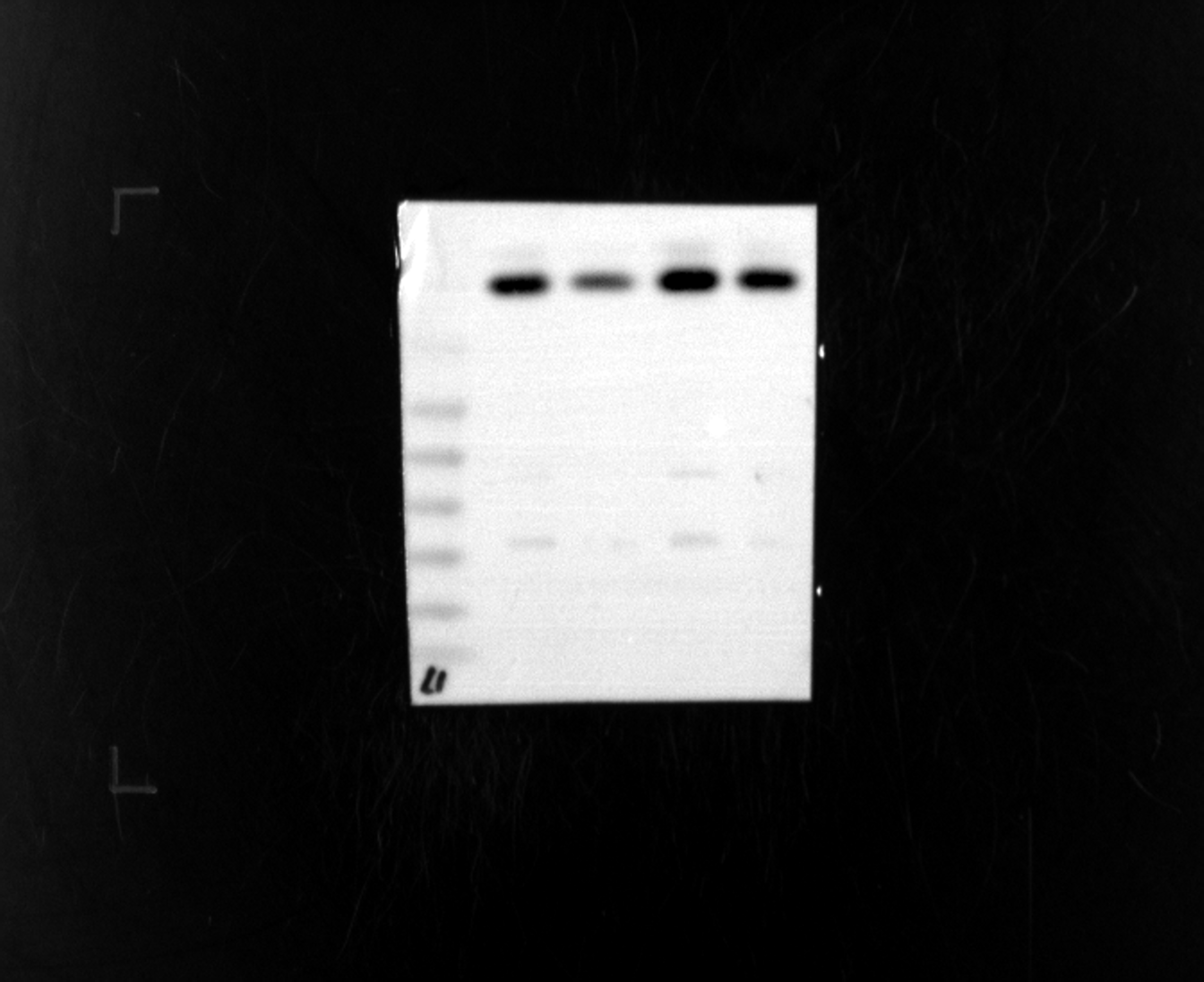

Supplement: Supplementary file 1 [file DataSheet1.zip › Supplementary Material/Fig5-WB/CAL-62/p-JAK1.Tif]

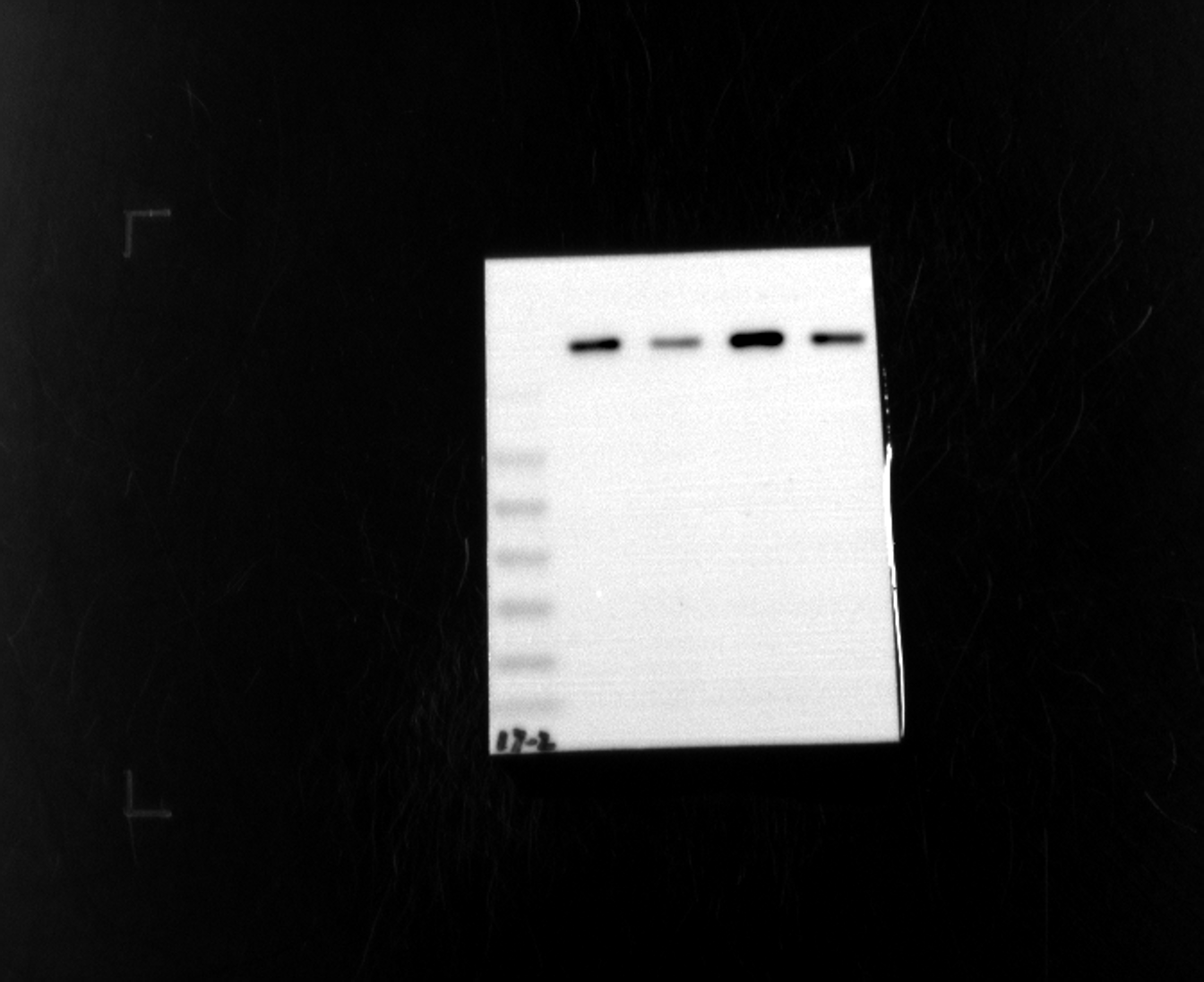

Supplement: Supplementary file 1 [file DataSheet1.zip › Supplementary Material/Fig5-WB/CAL-62/p-JAK2.Tif]

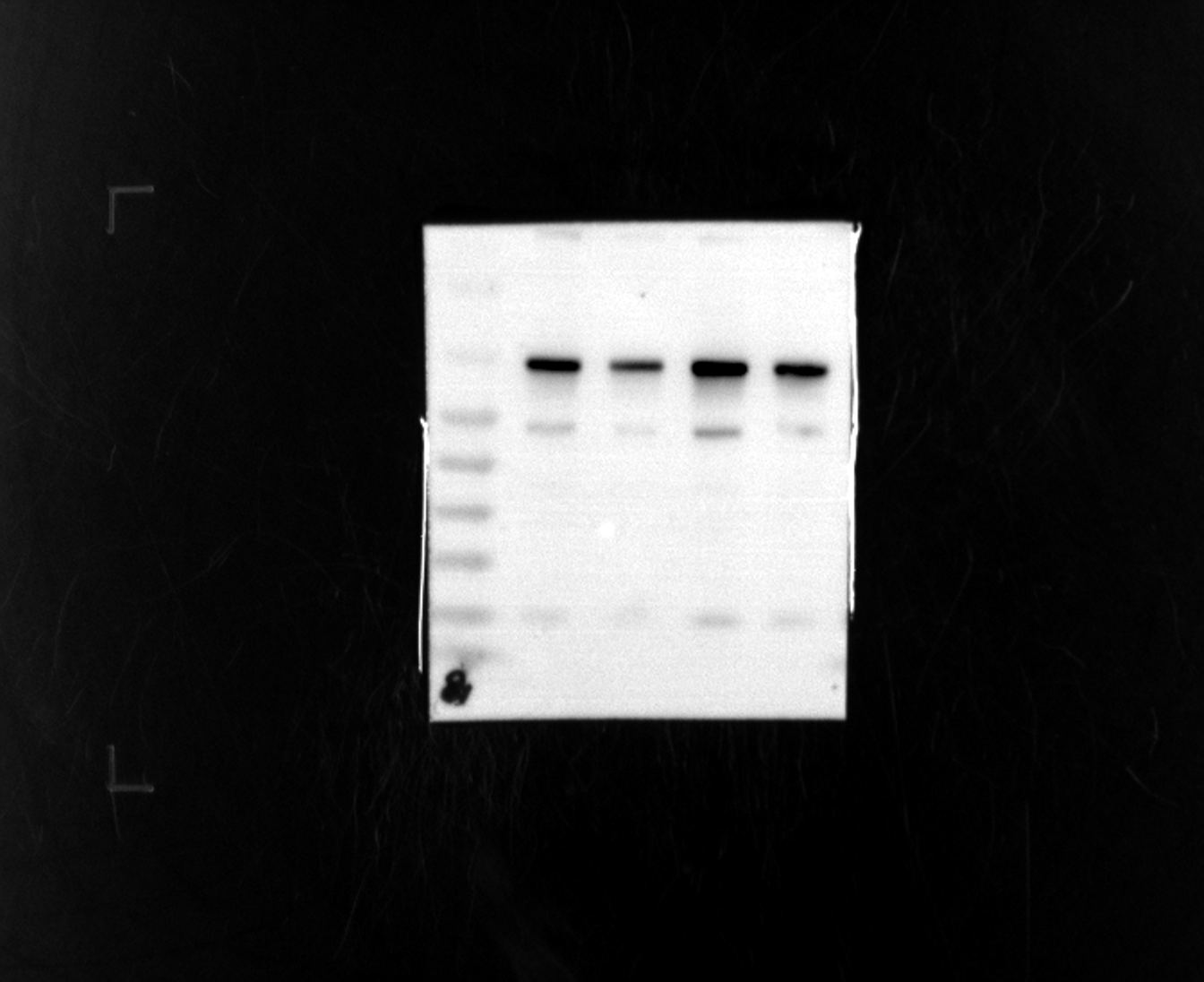

Supplement: Supplementary file 1 [file DataSheet1.zip › Supplementary Material/Fig5-WB/CAL-62/p-STAT3.Tif]

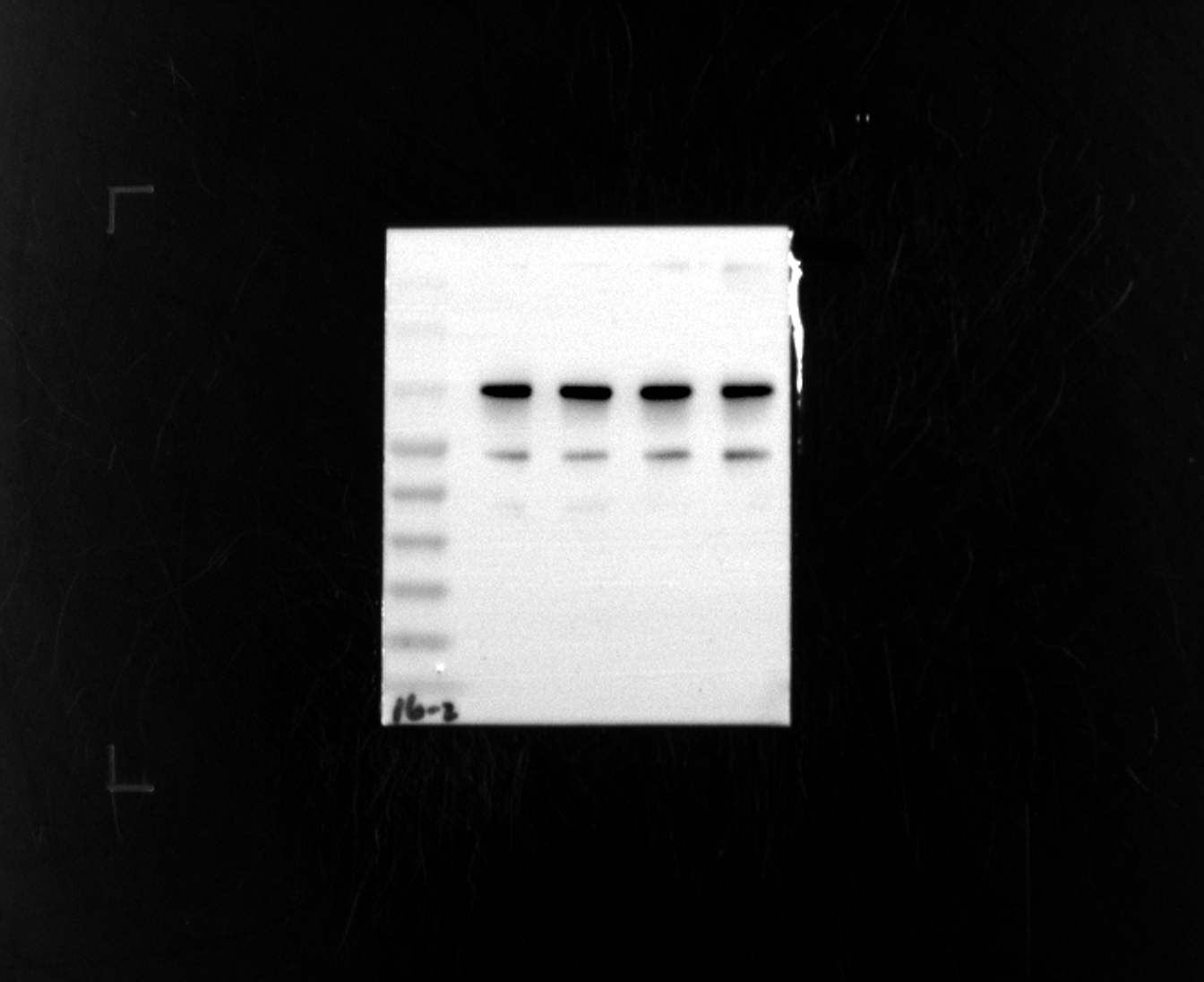

Supplement: Supplementary file 1 [file DataSheet1.zip › Supplementary Material/Fig5-WB/CAL-62/STAT3.Tif]

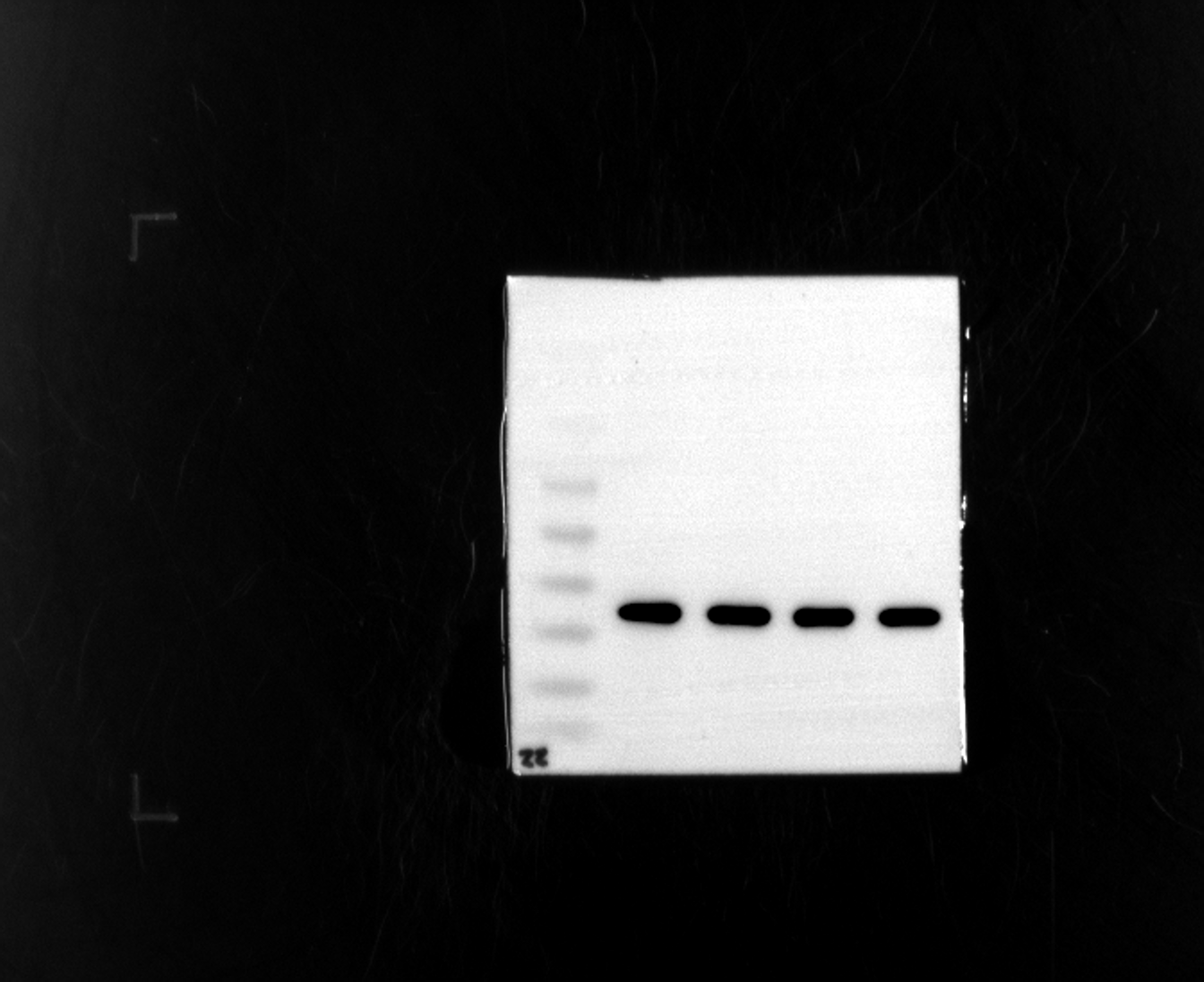

Supplement: Supplementary file 1 [file DataSheet1.zip › Supplementary Material/Fig6-WB/8505C/GAPDH.Tif]

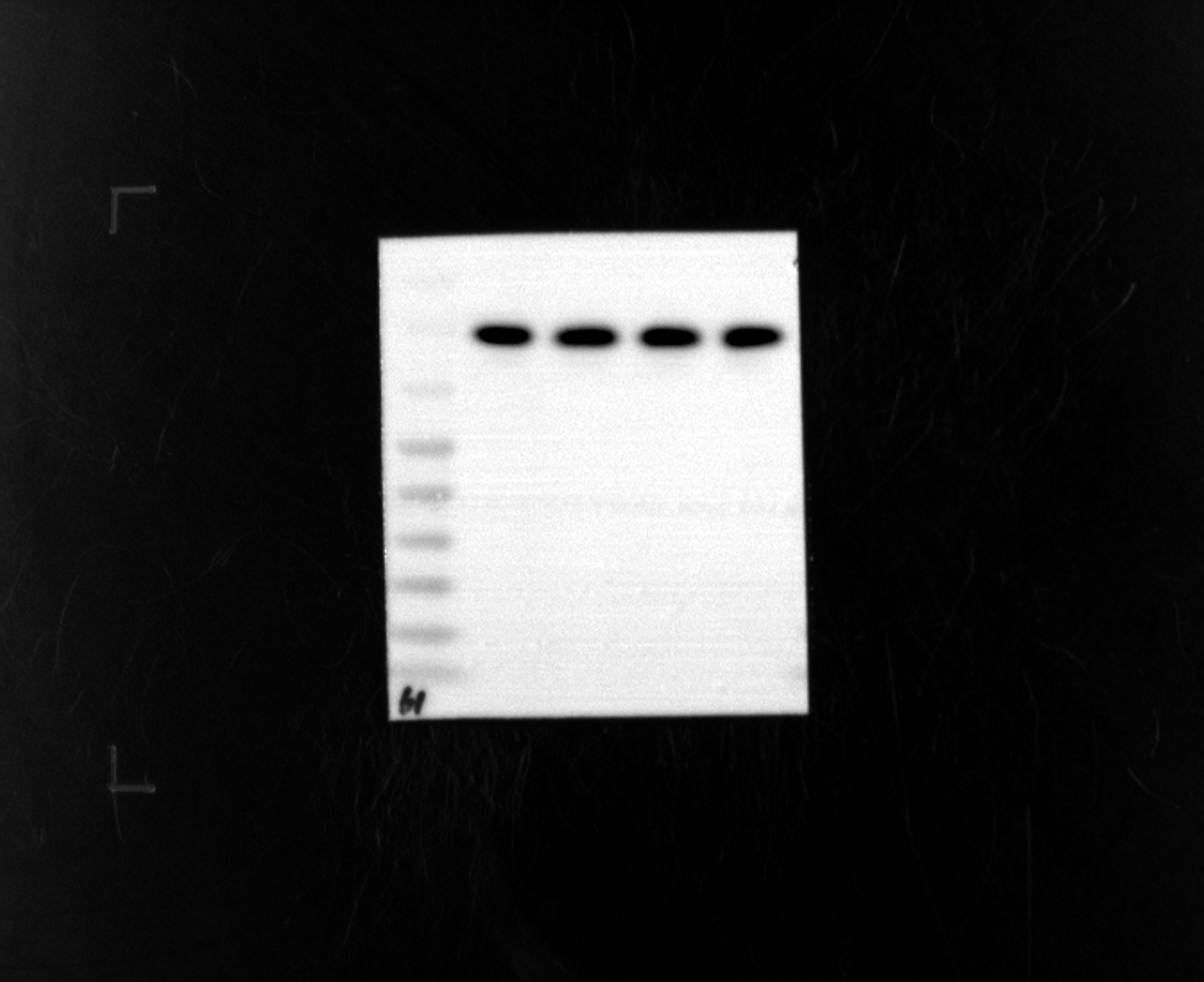

Supplement: Supplementary file 1 [file DataSheet1.zip › Supplementary Material/Fig6-WB/8505C/JAK1.Tif]

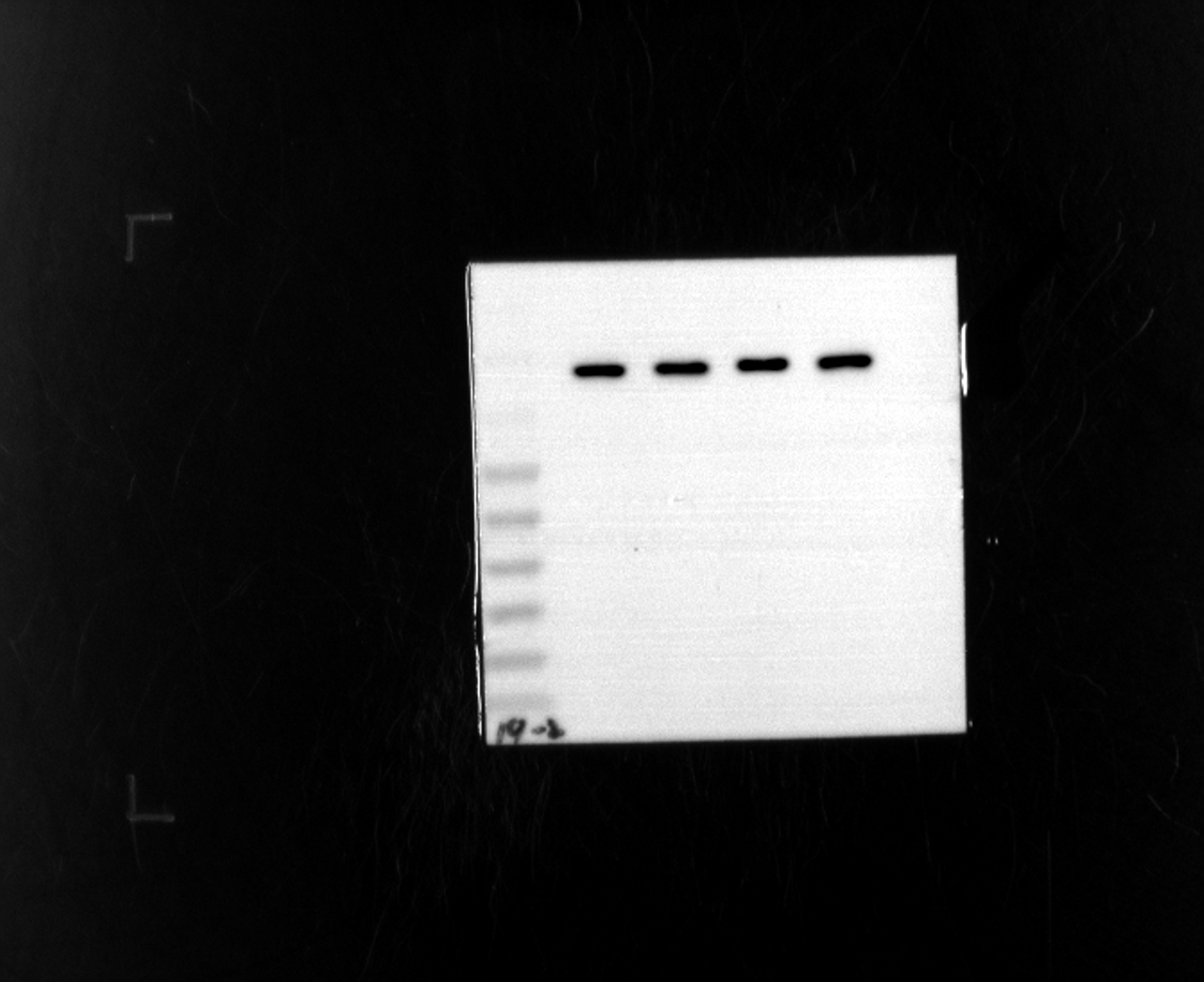

Supplement: Supplementary file 1 [file DataSheet1.zip › Supplementary Material/Fig6-WB/8505C/JAK2.Tif]

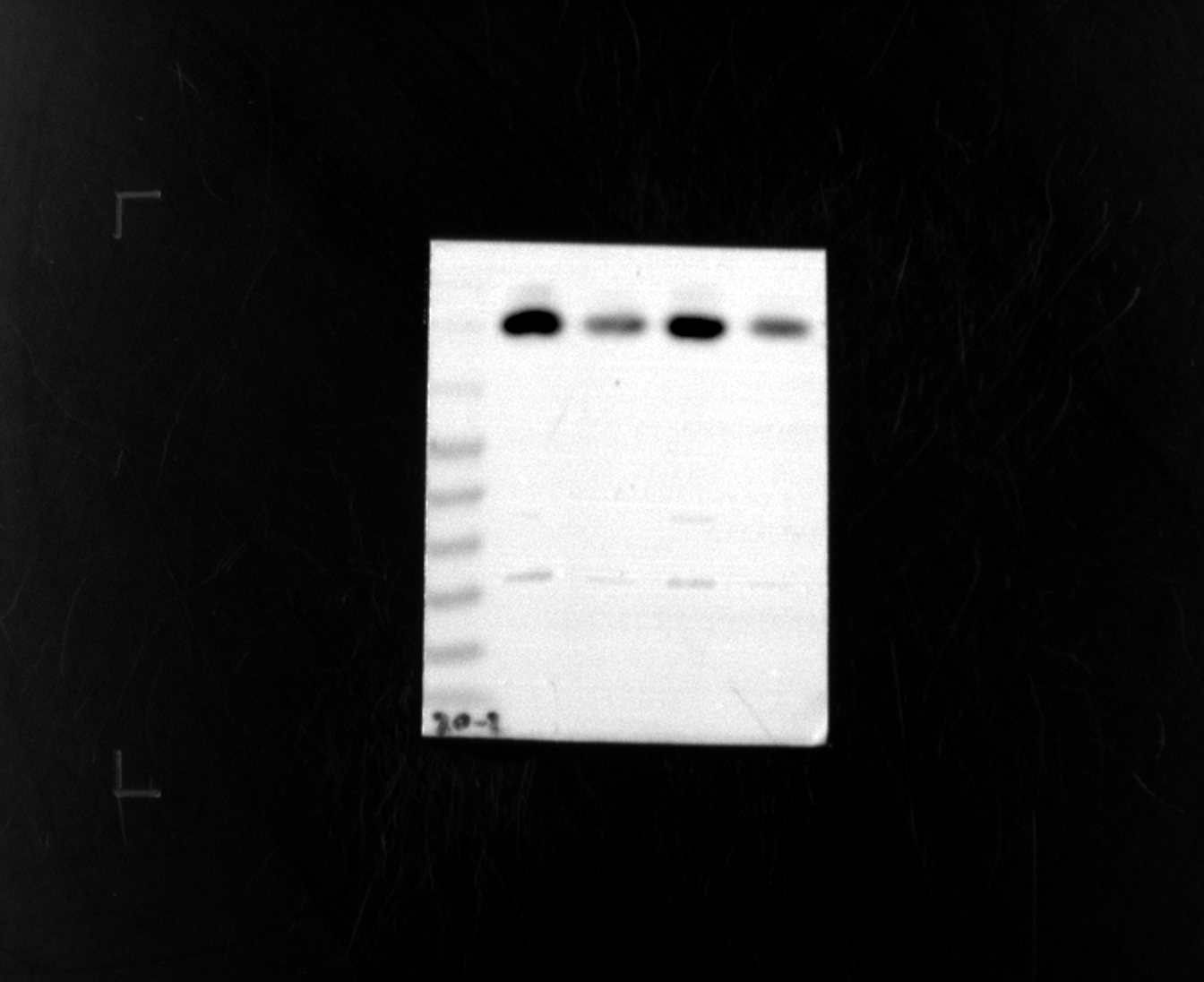

Supplement: Supplementary file 1 [file DataSheet1.zip › Supplementary Material/Fig6-WB/8505C/p-JAK1.Tif]

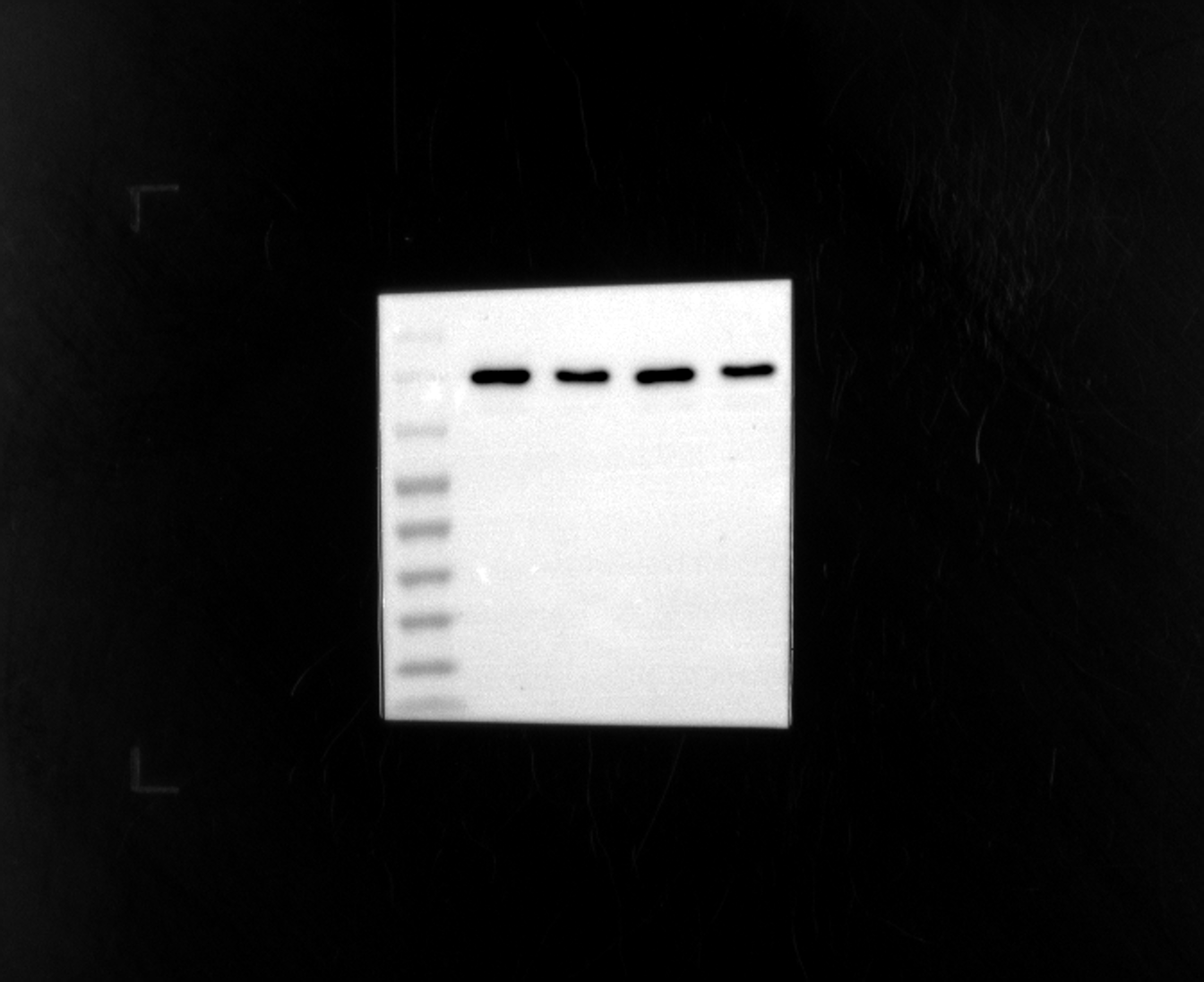

Supplement: Supplementary file 1 [file DataSheet1.zip › Supplementary Material/Fig6-WB/8505C/p-JAK2.Tif]

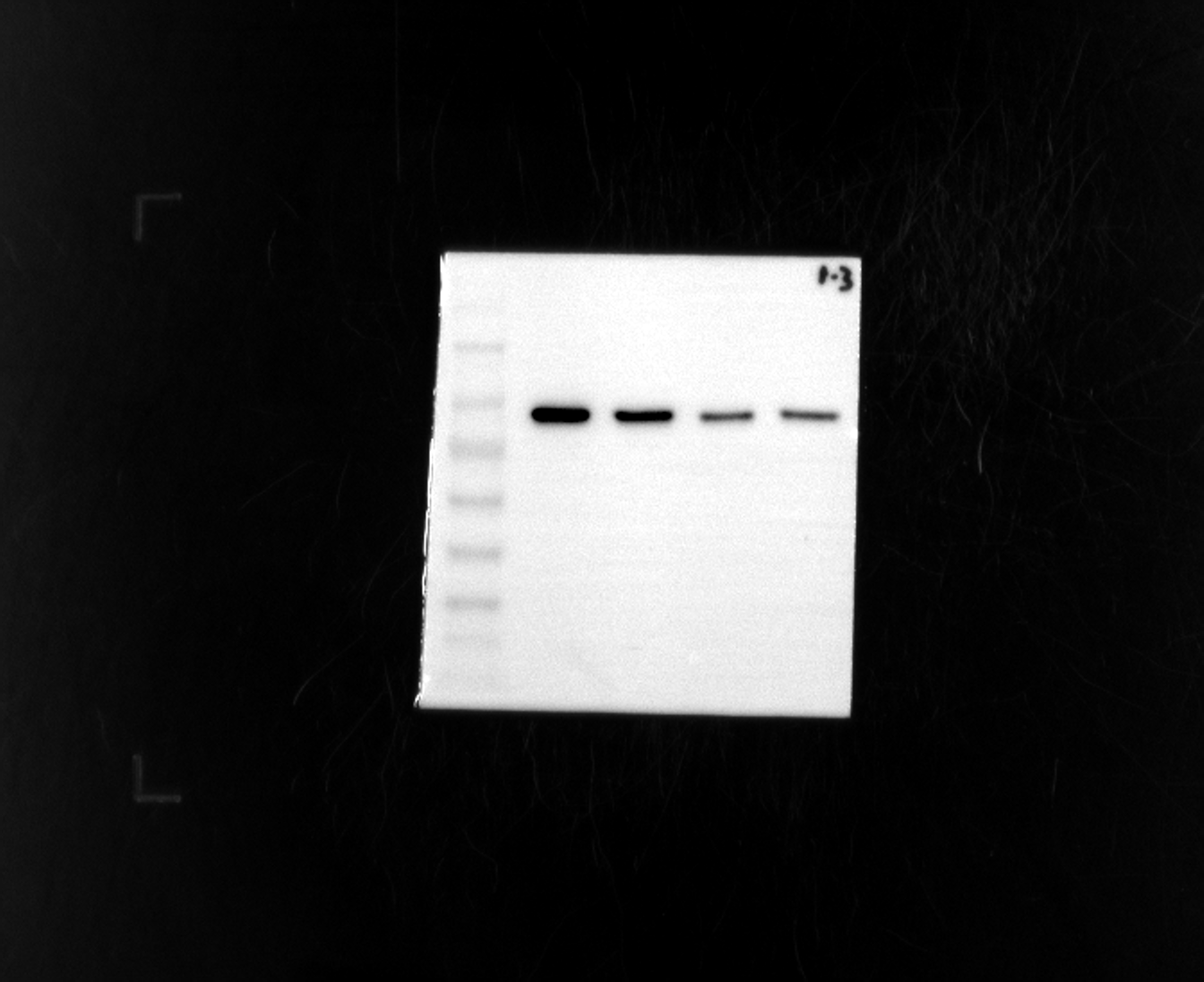

Supplement: Supplementary file 1 [file DataSheet1.zip › Supplementary Material/Fig6-WB/8505C/p-STAT3.Tif]

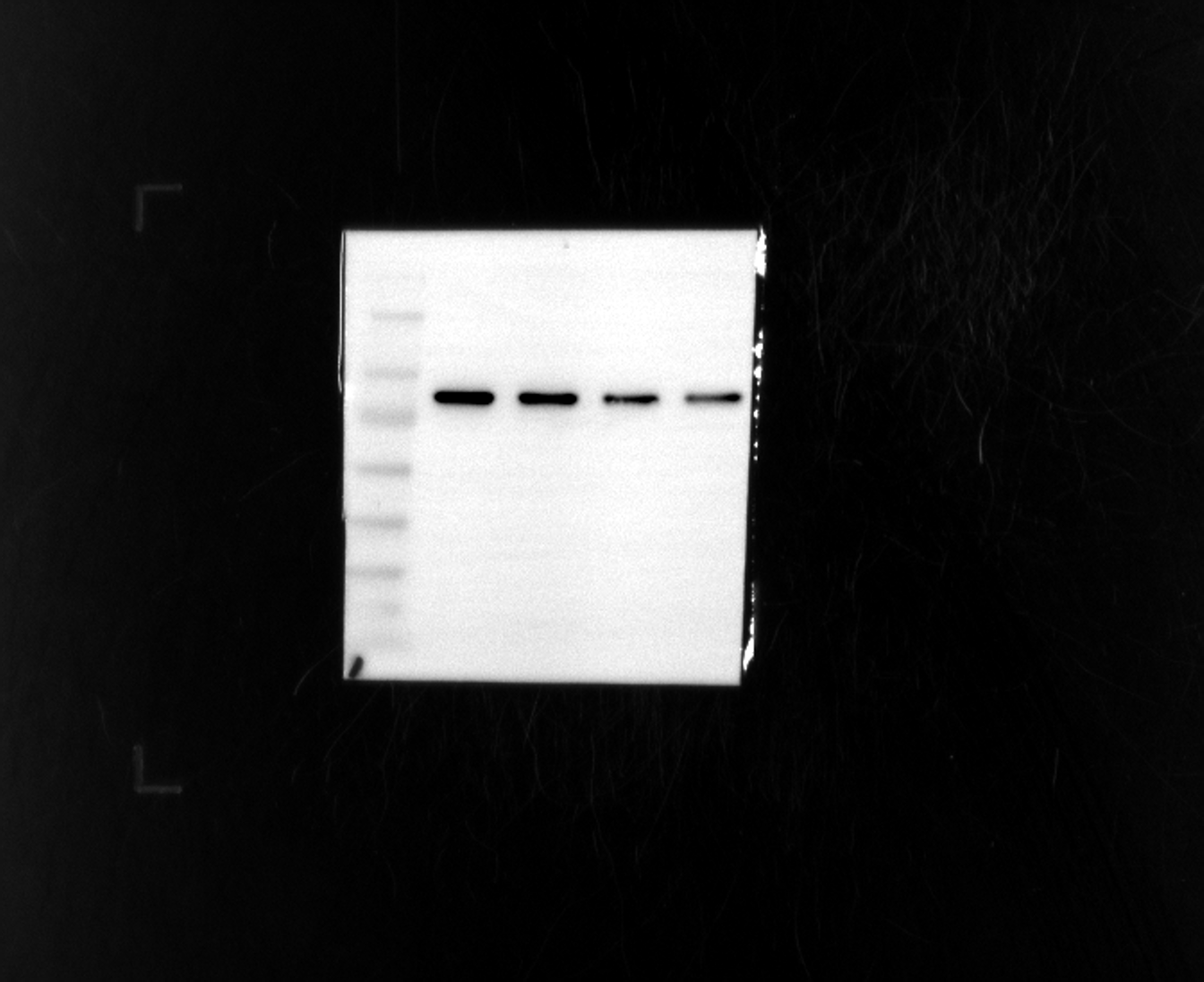

Supplement: Supplementary file 1 [file DataSheet1.zip › Supplementary Material/Fig6-WB/8505C/STAT3.Tif]

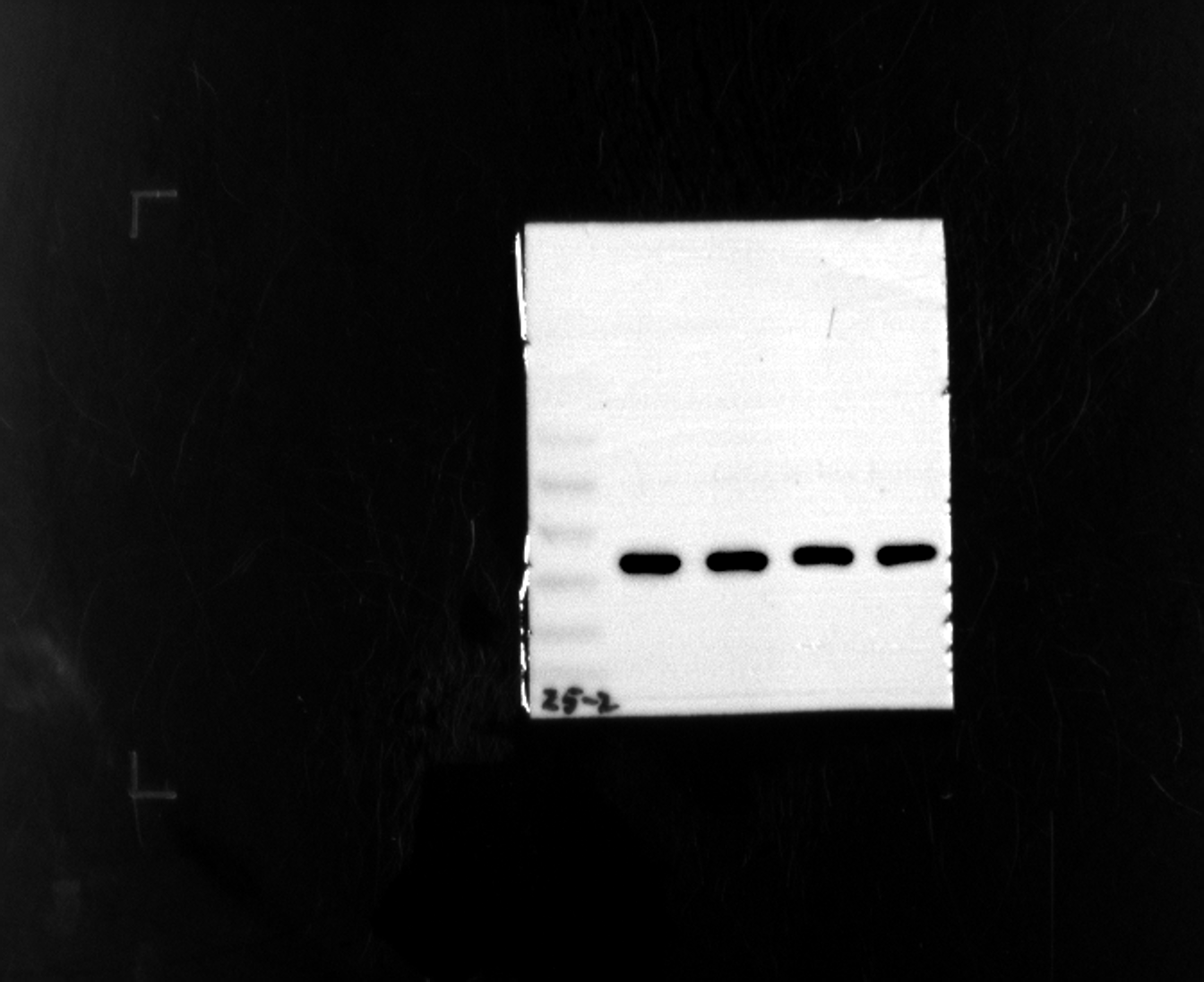

Supplement: Supplementary file 1 [file DataSheet1.zip › Supplementary Material/Fig6-WB/CAL-62/GAPDH.Tif]

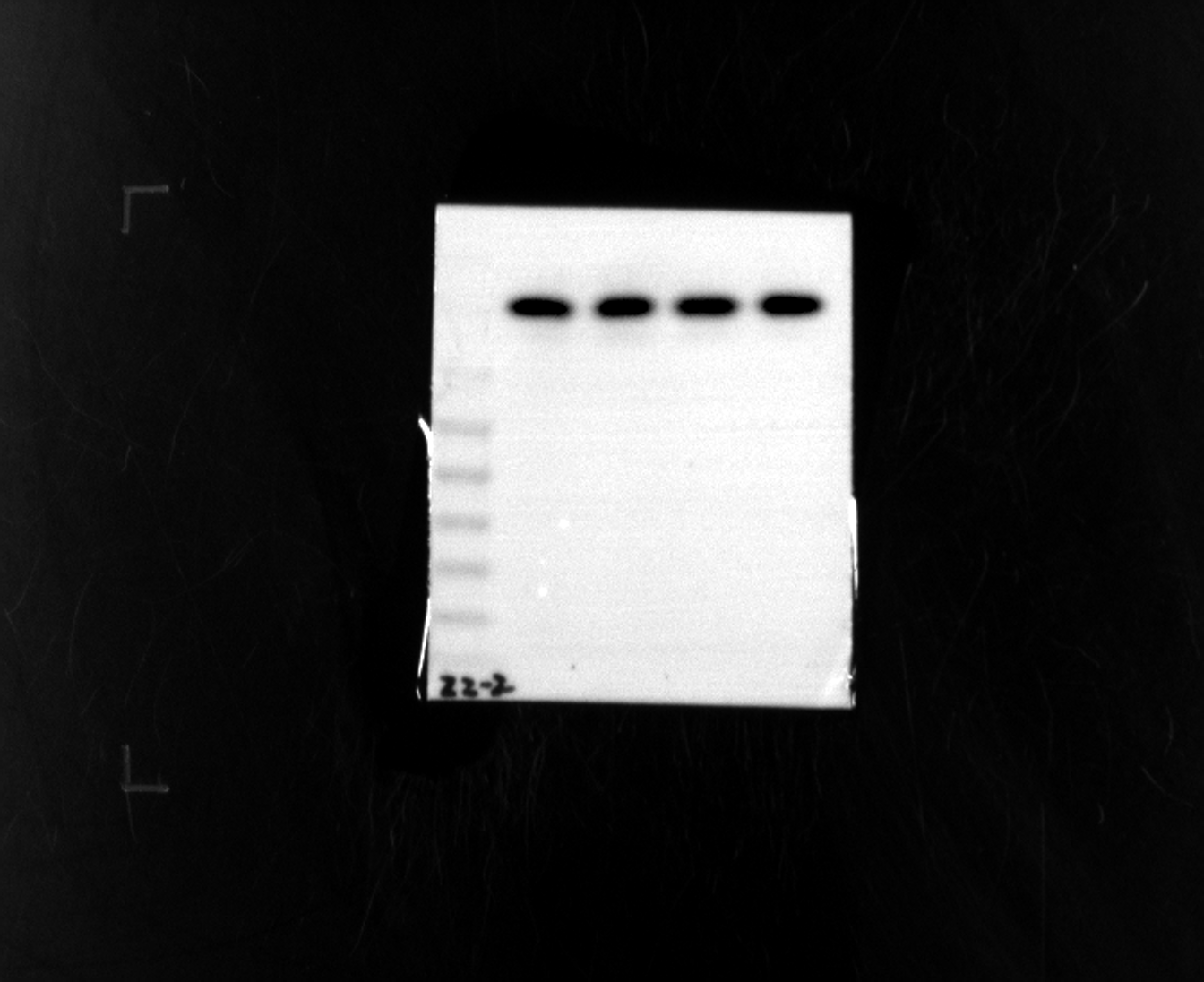

Supplement: Supplementary file 1 [file DataSheet1.zip › Supplementary Material/Fig6-WB/CAL-62/JAK1.Tif]

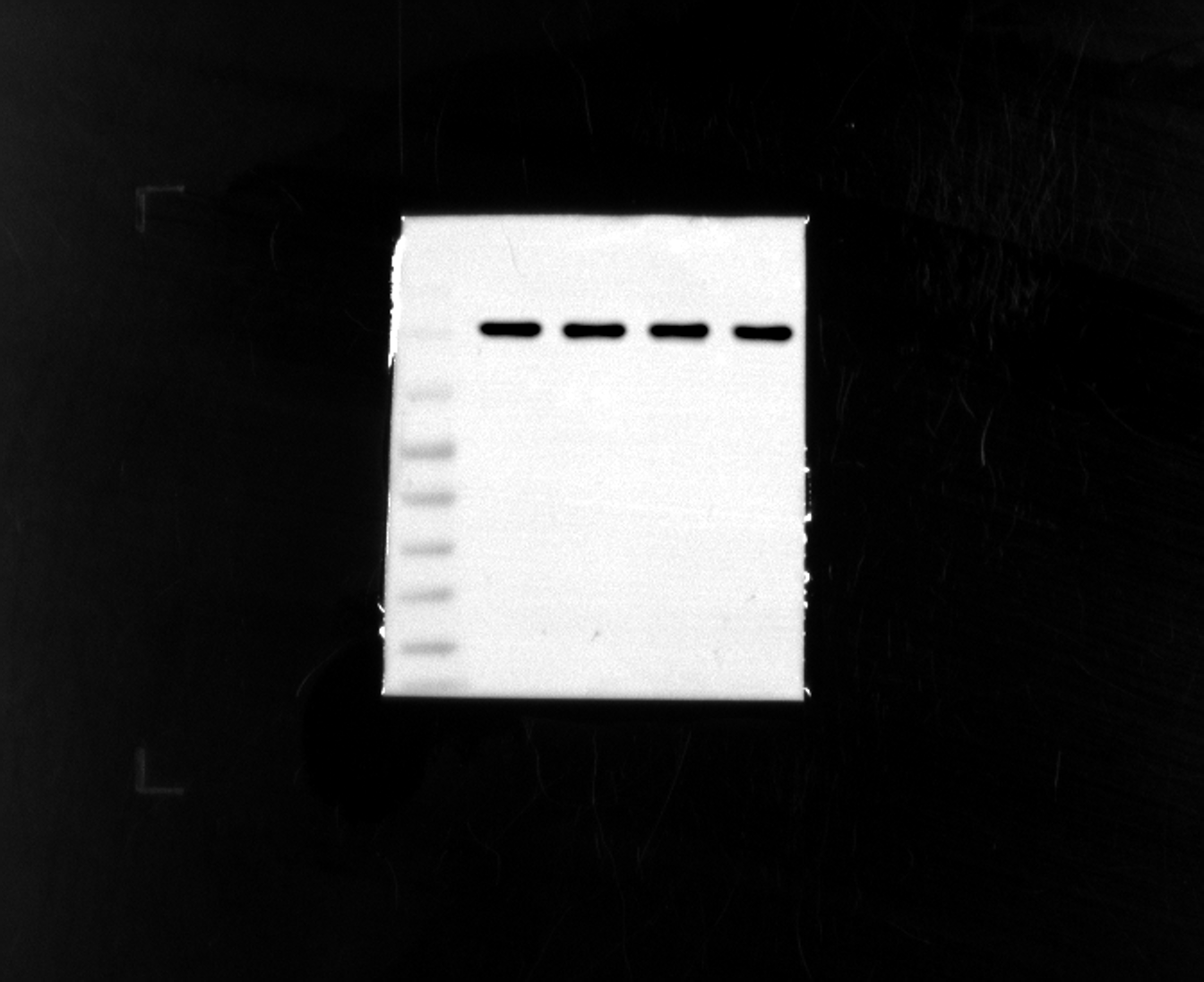

Supplement: Supplementary file 1 [file DataSheet1.zip › Supplementary Material/Fig6-WB/CAL-62/JAK2.Tif]

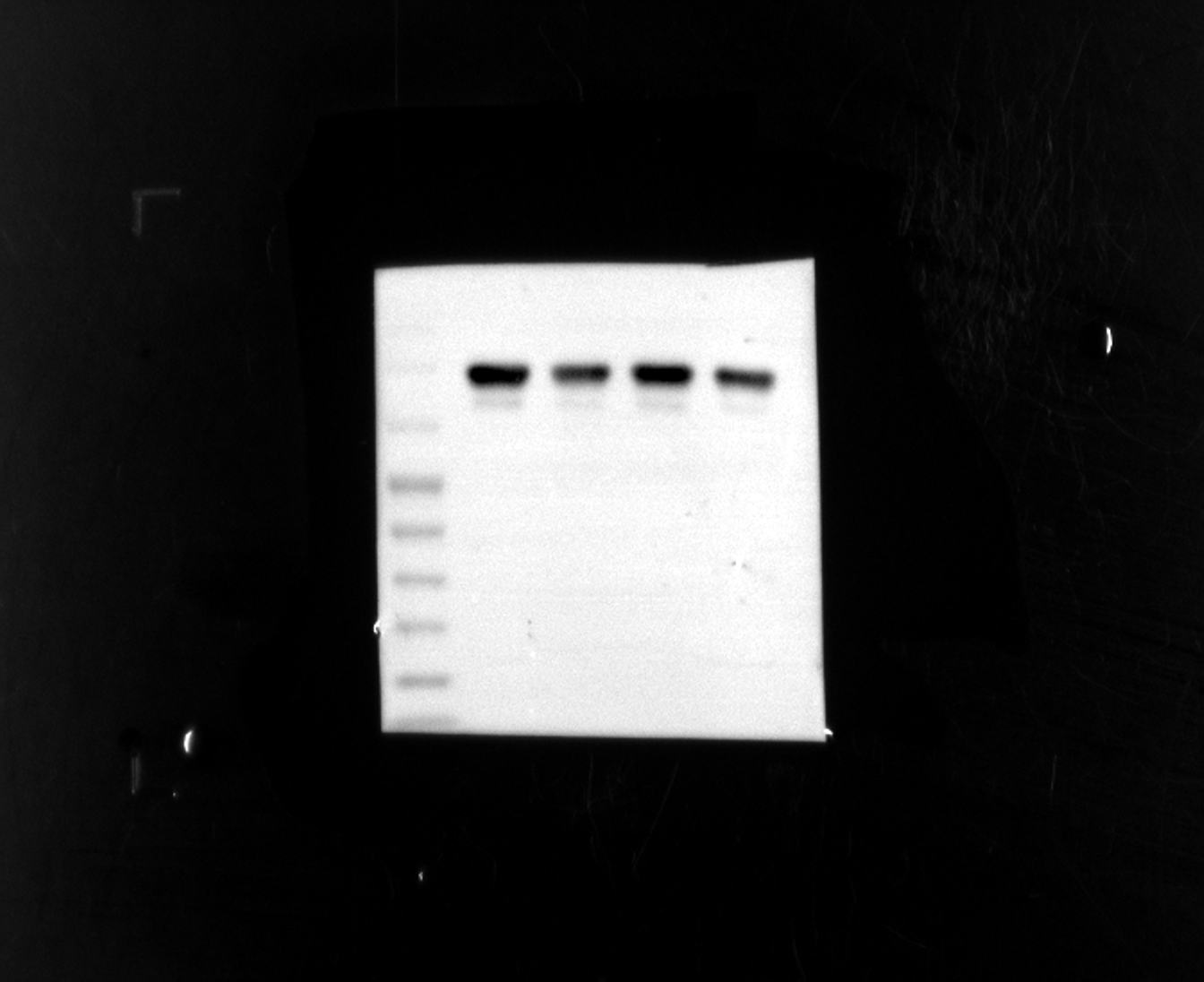

Supplement: Supplementary file 1 [file DataSheet1.zip › Supplementary Material/Fig6-WB/CAL-62/p-JAK1.Tif]

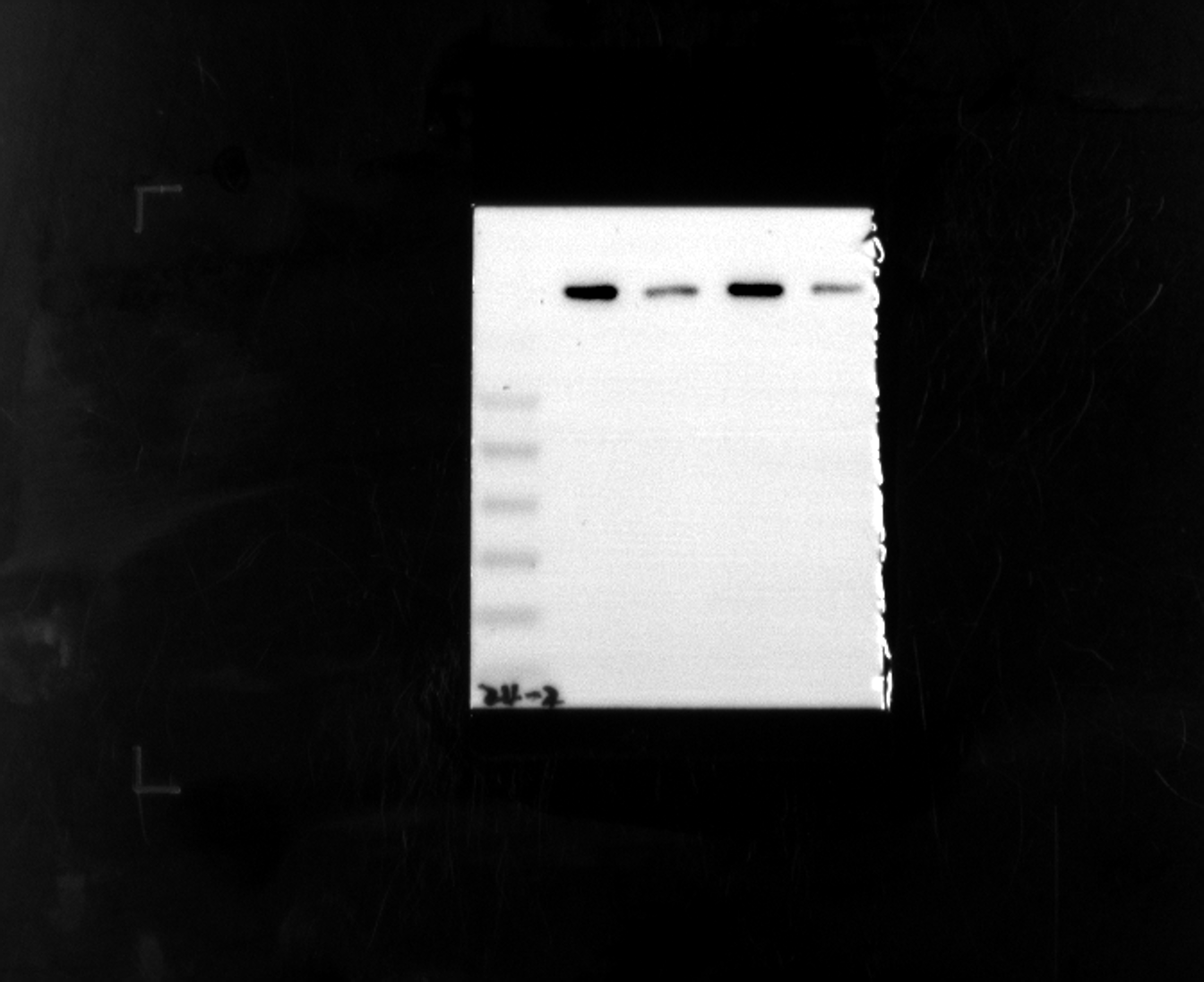

Supplement: Supplementary file 1 [file DataSheet1.zip › Supplementary Material/Fig6-WB/CAL-62/p-JAK2.Tif]

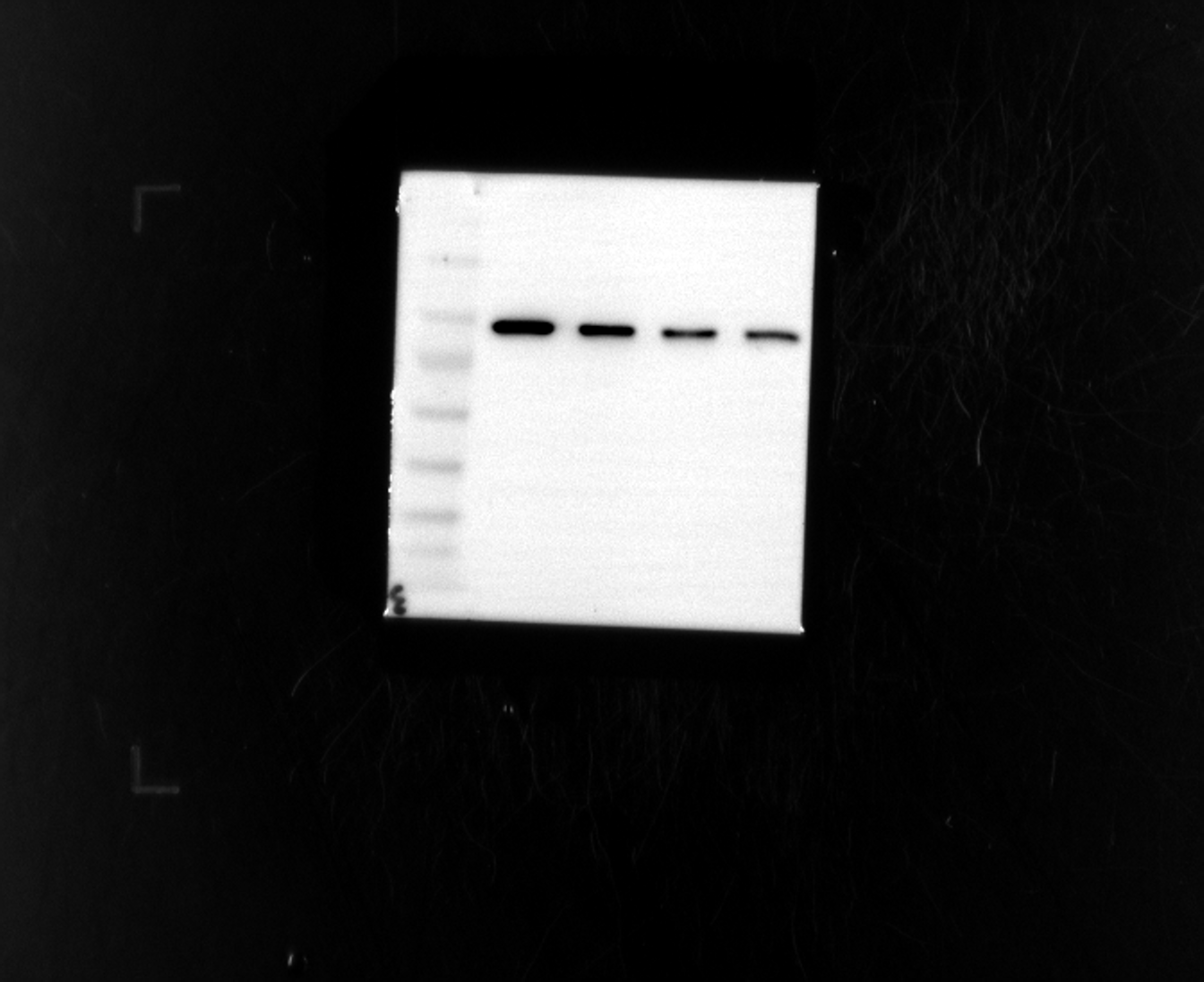

Supplement: Supplementary file 1 [file DataSheet1.zip › Supplementary Material/Fig6-WB/CAL-62/p-STAT3.Tif]

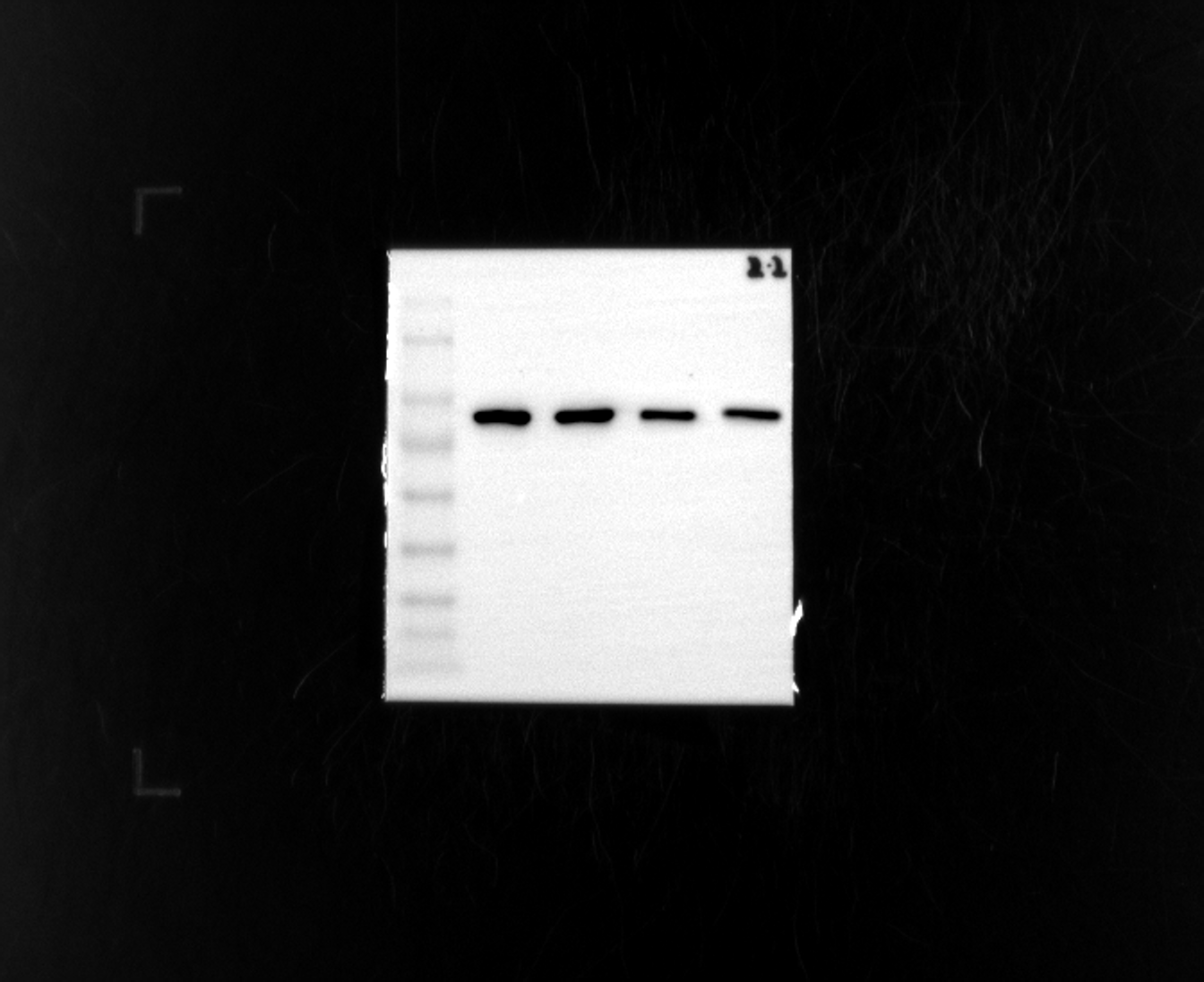

Supplement: Supplementary file 1 [file DataSheet1.zip › Supplementary Material/Fig6-WB/CAL-62/STAT3.Tif]

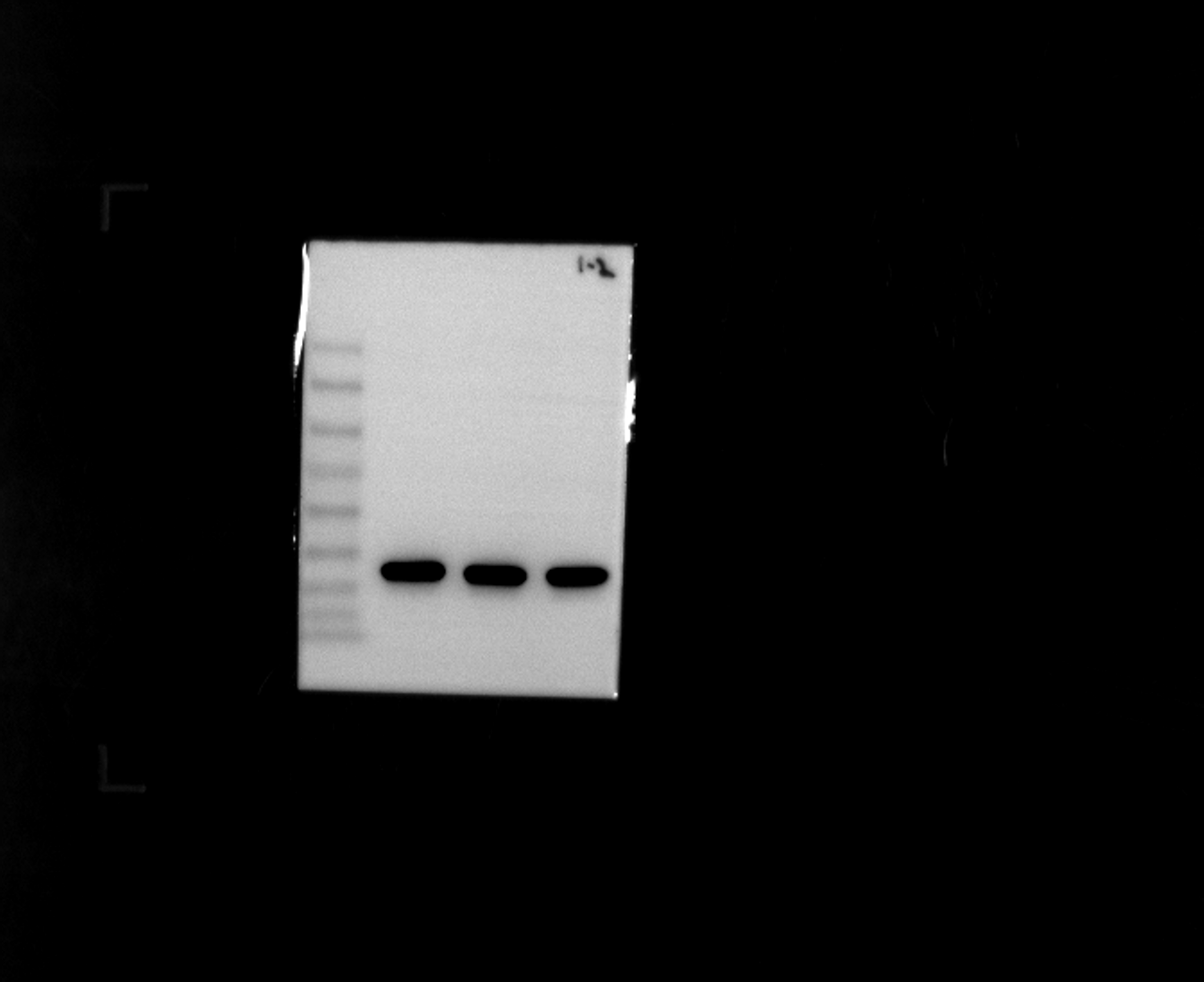

Supplement: Supplementary file 1 [file DataSheet1.zip › Supplementary Material/Fig7-WB/GAPDH.Tif]

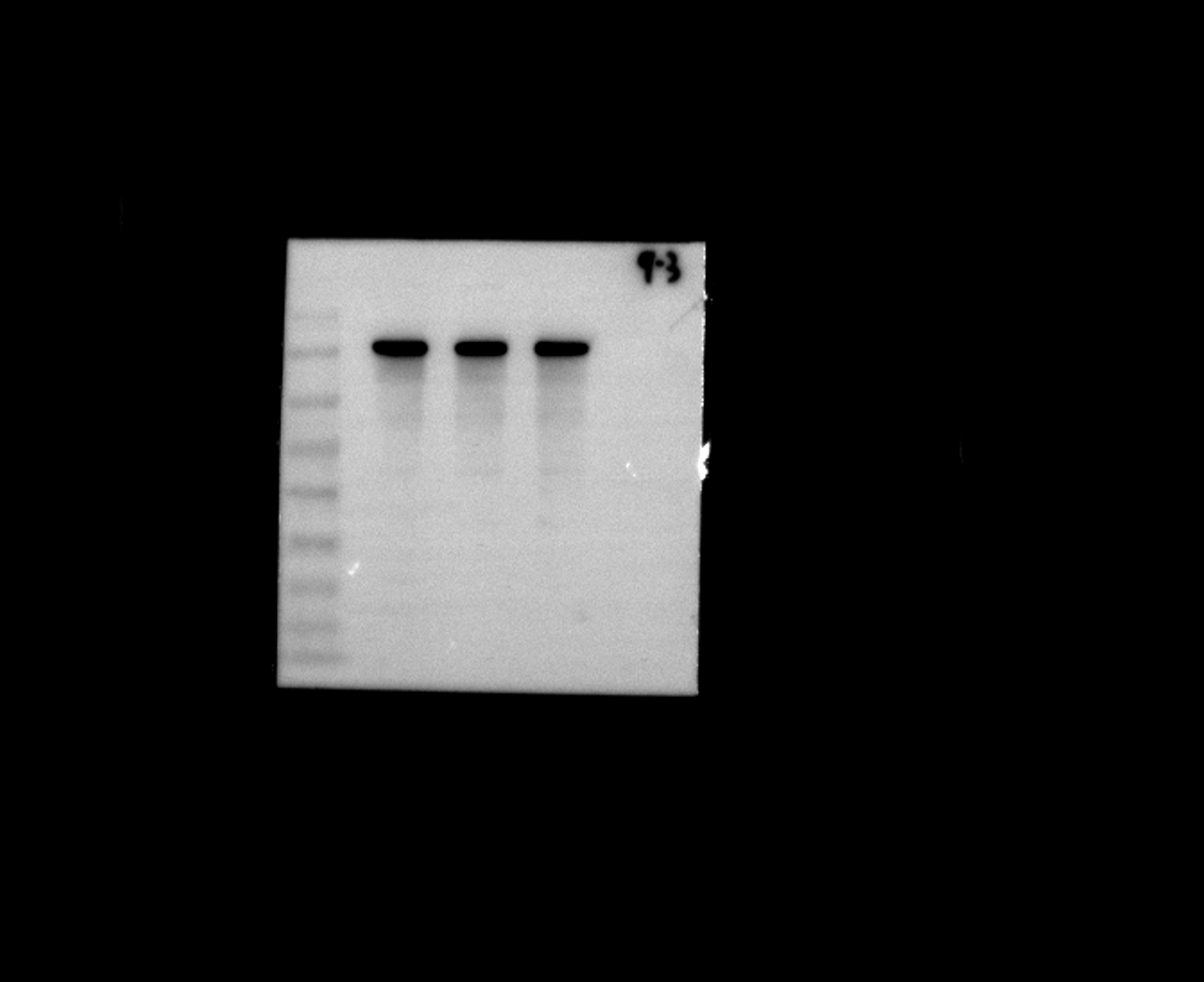

Supplement: Supplementary file 1 [file DataSheet1.zip › Supplementary Material/Fig7-WB/JAK1.Tif]

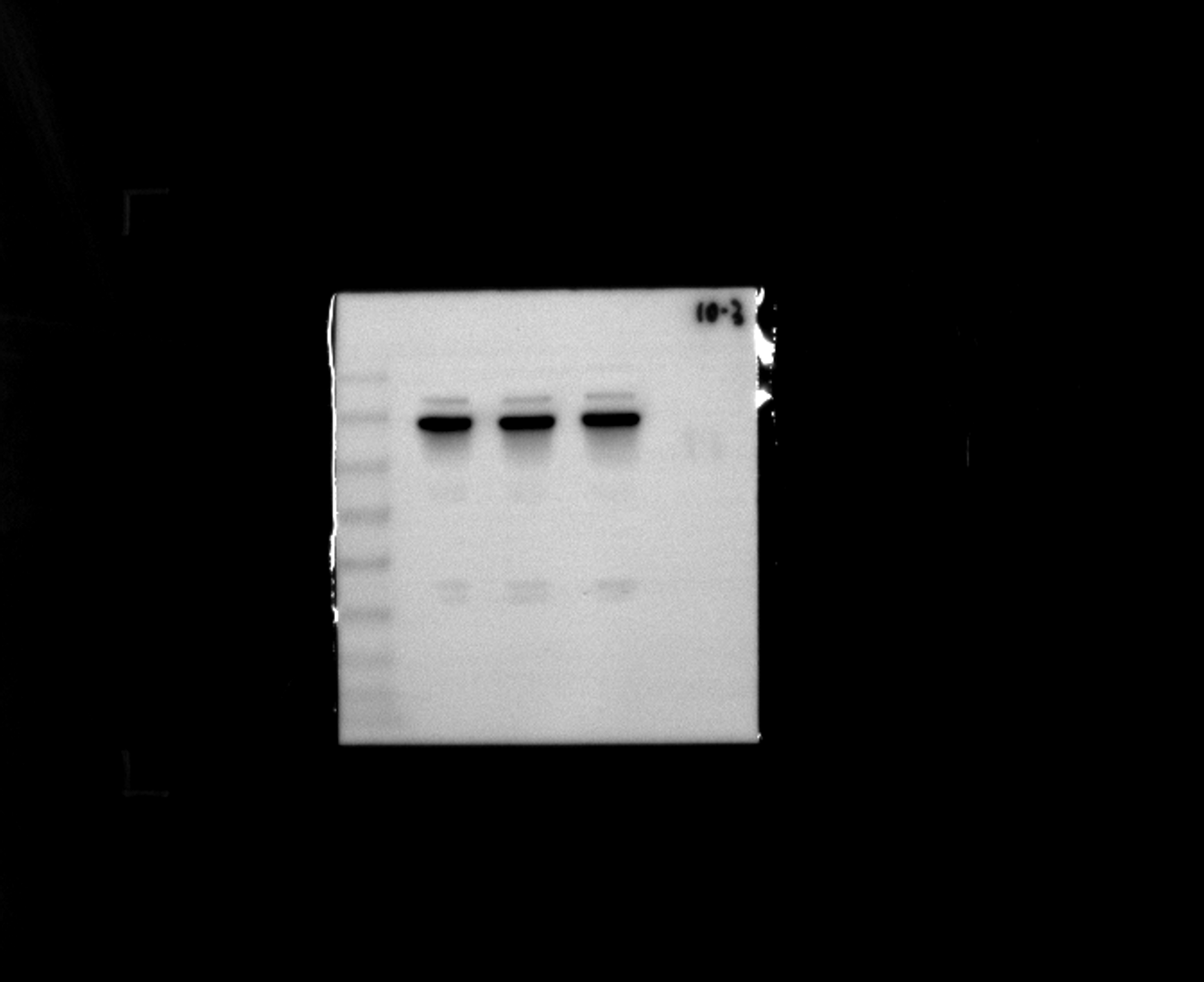

Supplement: Supplementary file 1 [file DataSheet1.zip › Supplementary Material/Fig7-WB/JAK2.Tif]

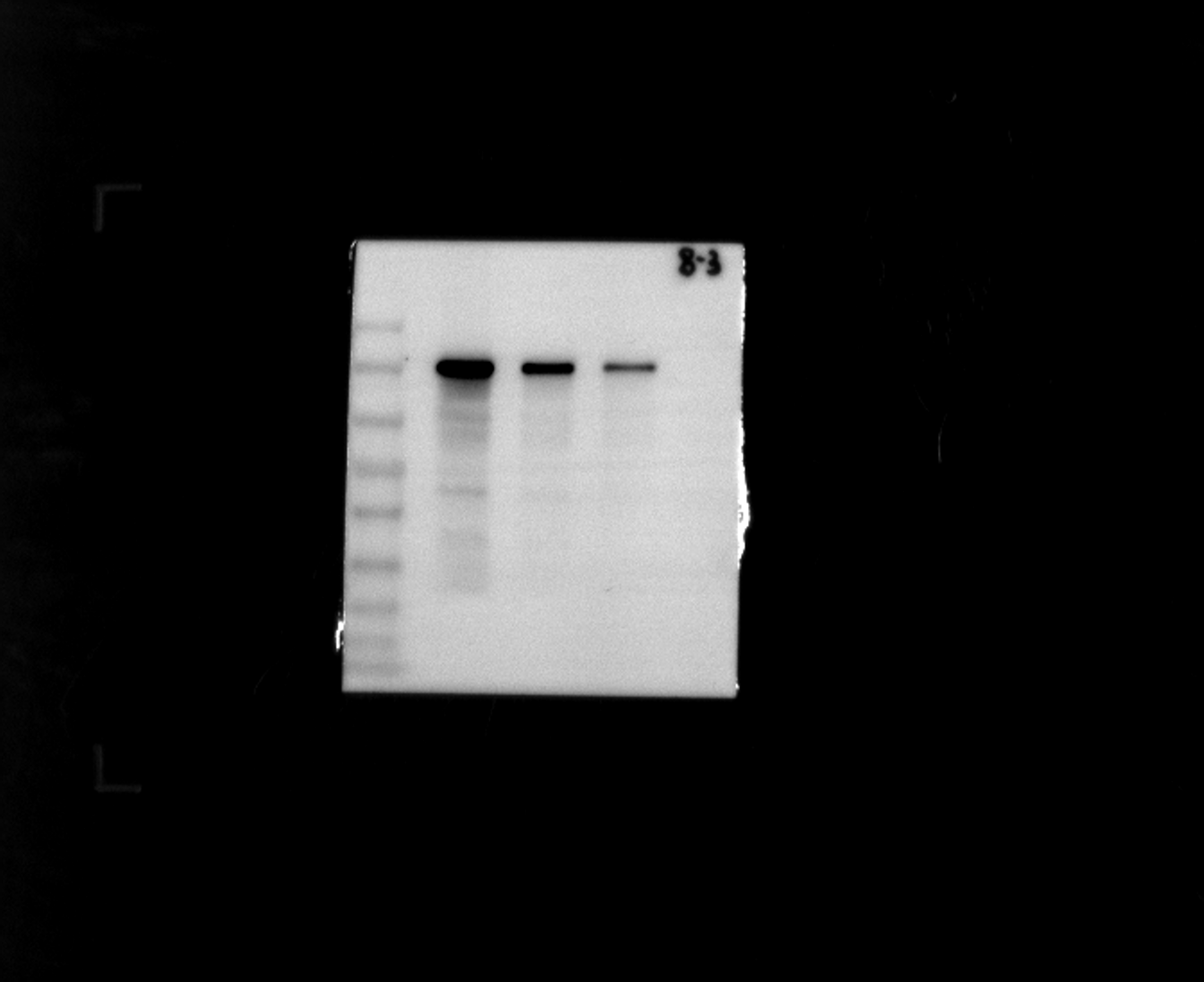

Supplement: Supplementary file 1 [file DataSheet1.zip › Supplementary Material/Fig7-WB/p-JAK1.Tif]

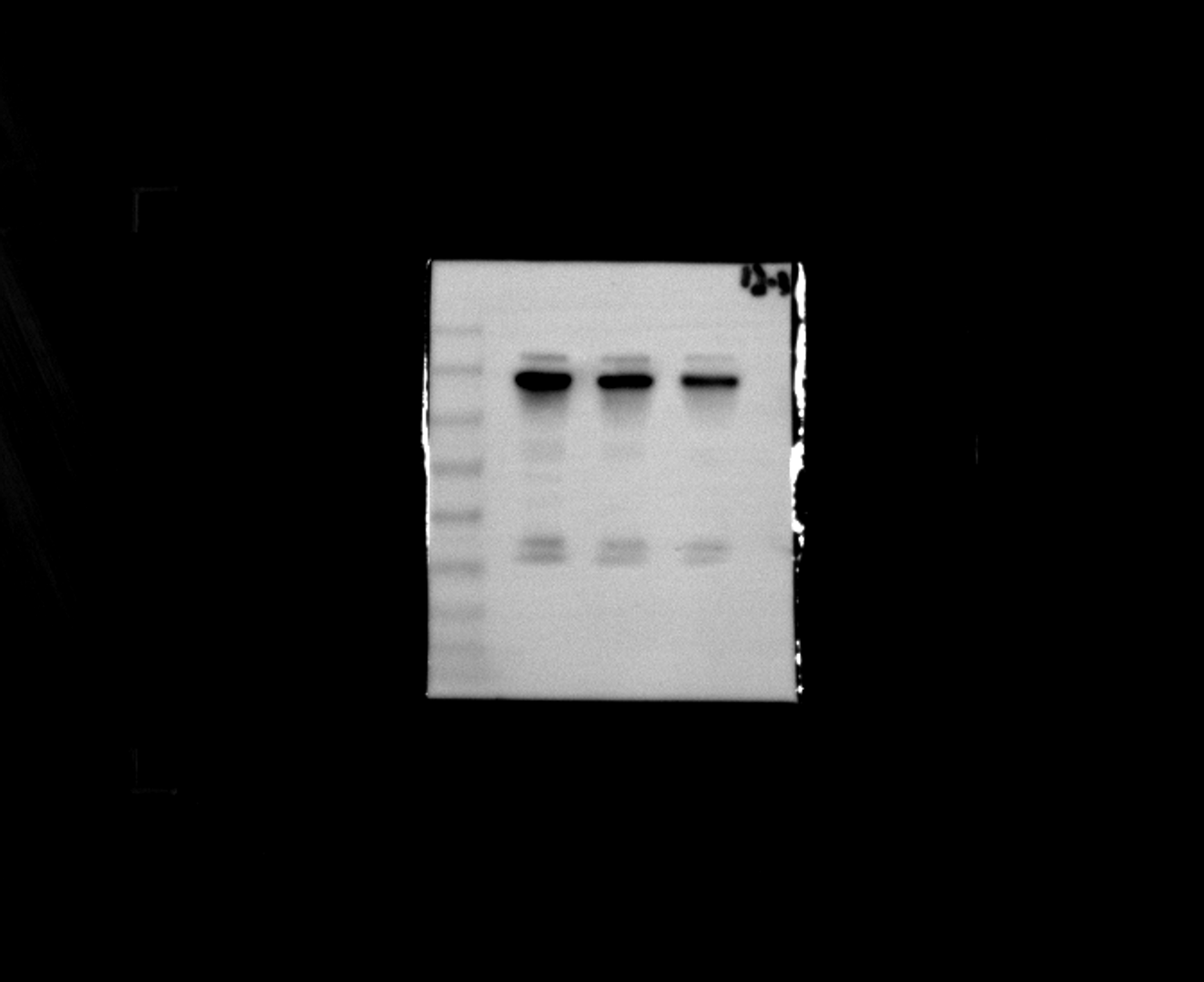

Supplement: Supplementary file 1 [file DataSheet1.zip › Supplementary Material/Fig7-WB/p-JAK2.Tif]

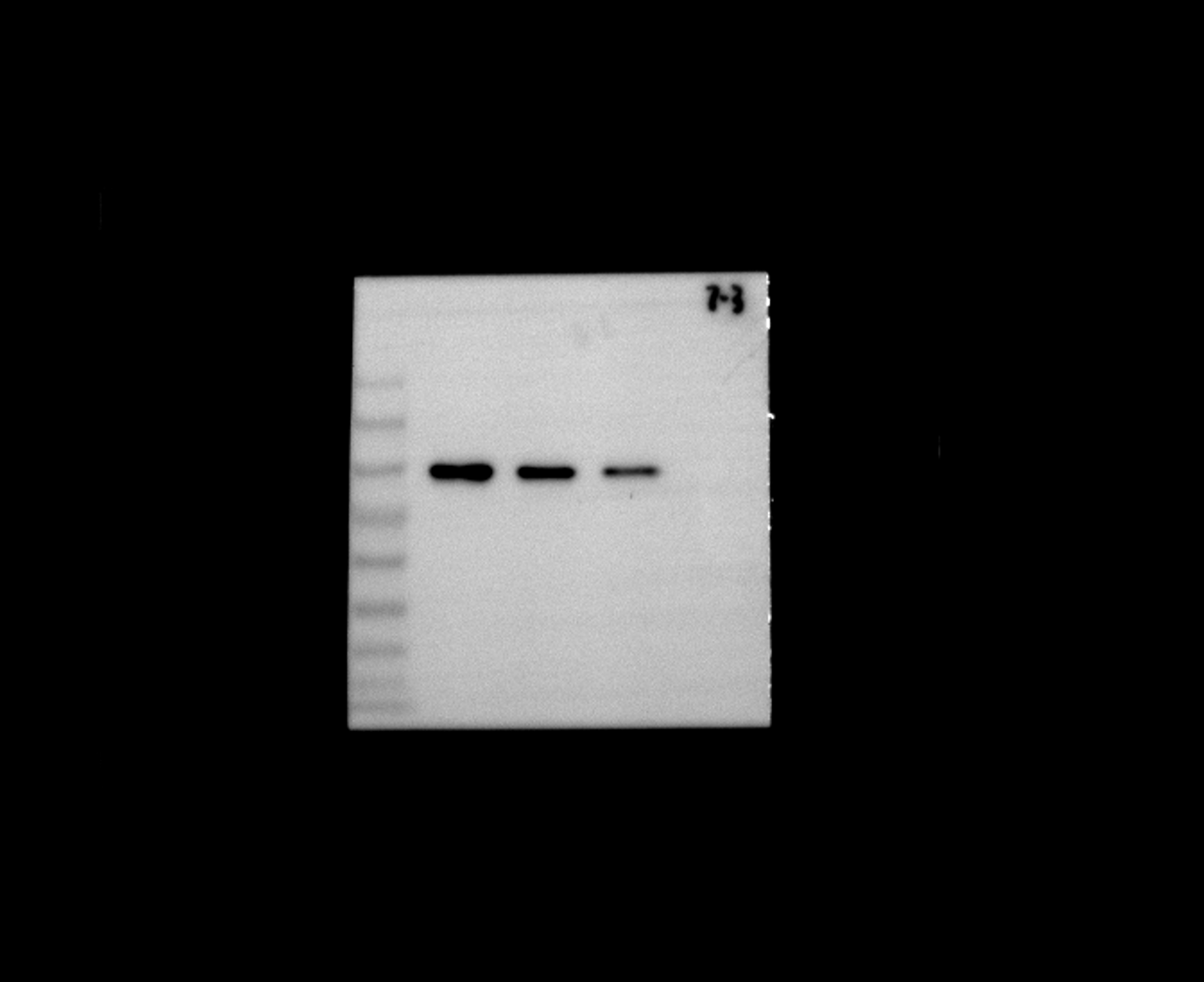

Supplement: Supplementary file 1 [file DataSheet1.zip › Supplementary Material/Fig7-WB/p-STAT3.Tif]

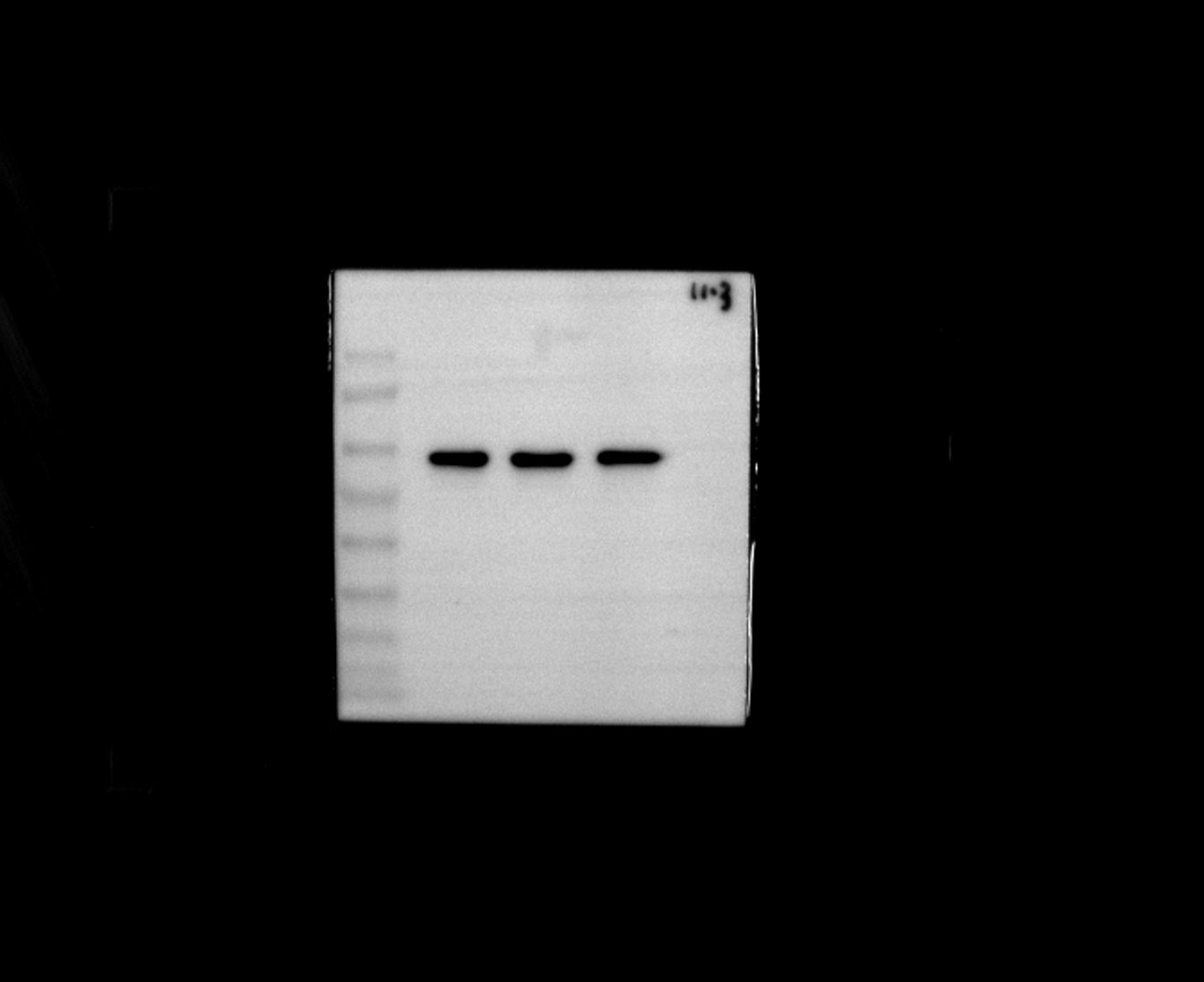

Supplement: Supplementary file 1 [file DataSheet1.zip › Supplementary Material/Fig7-WB/STAT3.Tif]
